# Supplementary material for: Costs associated with retinopathy of prematurity: a systematic review and meta-analysis
Source: BMJ Open. 2022 Nov 23;12(11):e057864. doi: 10.1136/bmjopen-2021-057864 (PMC9693652; doi:10.1136/bmjopen-2021-057864)
Supplement: Supplementary data [file bmjopen-2021-057864supp001.pdf]

Online-Only Supplements

# Costs associated with Retinopathy of prematurity: A Systematic Review and Meta-analysis


## Authors

Hanna Gyllensten[1,2], Associate professor

Jhangir Humayun[1,2], B.Sc.

Ulrika Sjöbom[1,3], M.Sc.

Ann Hellström[3], Professor

Chatarina Löfqvist[1,2,3], Associate professor

### Author affiliations

[1] Institute of Health and Care Sciences, Sahlgrenska Academy, University of Gothenburg, Box 457, SE-405 30 Gothenburg, Sweden.

[2] Centre for Person-Centred Care (GPCC), University of Gothenburg, Box 457, SE-405 30 Gothenburg, Sweden.

[3] Department of Clinical Neuroscience, Institute of Neuroscience and Physiology, Sahlgrenska Academy, University of Gothenburg, Gothenburg, Sweden.


## Content


Authors ........ 1

  Author affiliations ........ 1

eTable 1. Search terms ........ 2

eTable 2. Data extraction sheet ........ 2

  Data extraction ........ 2

  Quality assessment (according to instrument developed by Evers et al[1]) ........ 2

eTable 3. Checklist for the quality appraisal of included papers (from Evers et al[1]) ........ 2

eFigure 1. Flow diagram shows the study selection process, following the PRISMA guidelines.[21] ........ 4

eTable 4. Excluded articles* ........ 5

eFigure 2. Cost model ........ 6

  Preterm birth ........ 7

  ROP screening ........ 7

  Lifetime (treatment and follow-up) ........ 7

References ........ 8

## eTable 1. Search strategy[a]

| Database | Search string |
|---|---|
| Pubmed | (((((((Retinopathy) AND Prematur*) OR ((Terry) AND Syndrom*) OR ("ROP"[Title/Abstract]) OR "Retinopathy of Prematurity"[Mesh])) AND (("Economics"[Mesh]) OR ((economic*[Title/Abstract] OR cost[Title/Abstract] OR costs[Title/Abstract] OR costly[Title/Abstract] OR costing[Title/Abstract] OR price[Title/Abstract] OR prices[Title/Abstract] OR pricing[Title/Abstract] OR pharmacoeconomic*[Title/Abstract])))))) |
| Scopus | ( TITLE-ABS-KEY ( "Retinopath*" ) AND TITLE-ABS-KEY ( "Prematur*" ) ) OR ( TITLE-ABS-KEY ( "Retrolental" ) AND TITLE-ABS-KEY ( "Fibroplas*" ) ) OR ( TITLE-ABS-KEY ( "Terry" ) AND TITLE-ABS-KEY ( "Syndrom*" ) ) AND ( TITLE-ABS-KEY ( economic* OR cost OR cos OR costly OR costing OR price OR prices OR pricing OR pharmacoeconomic* ) ) |

[a] No filters or limitations were used in the searches of databases.

## eTable 2. Data extraction sheet

**Data extraction**

- Reviewer
- Reference (APA)
- Aim/Objective
- Study design
- When was it conducted
- Setting including country and hospital name/database
- How is ROP severity defined
- Total study participants
- Patients with ROP (N)
- Patient group description
- Controls (N)
- Control group description
- Average cost of screening (total per infant/per visit/per eye)
- What costs are measured
- How are the costs measured
- Average Cost for infants with diagnosed sight-threatening ROP
- What costs are measured
- How are the costs measured
- Costs from which year (if adjusted, which year)
- Perspective: cost analysis
- Time horizon of cost analysis
- Funding
- Limitations: Confounders and biases reported
- Conclusions (by author)

**Quality assessment (according to instrument developed by Evers et al[1])**

1. Is the study population clearly described?
2. Are competing alternatives clearly described?
3. Is a well-defined research question posed in answerable form?
4. Is the economic study design appropriate to the stated objective?
5. Is the chosen time horizon appropriate in order to include relevant costs and consequences?
6. Is the actual perspective chosen appropriate?
7. Are all important and relevant costs for each alternative identified?
8. Are all costs measured appropriately in physical units?
9. Are costs valued appropriately?
10. Are all important and relevant outcomes for each alternative identified?
11. Are all outcomes measured appropriately?
12. Are outcomes valued appropriately?
13. Is an incremental analysis of costs and outcomes of alternatives performed?
14. Are all future costs and outcomes discounted appropriately?
15. Are all important variables, whose values are uncertain, appropriately subjected to sensitivity analysis?
16. Do the conclusions follow from the data reported?
17. Does the study discuss the generalizability of the results to other settings and patient/client groups?
18. Does the article indicate that there is no potential conflict of interest of study researcher(s) and funder(s)?
19. Are ethical and distributional issues discussed appropriately

**eTable 3. Checklist for the quality appraisal of included papers (from Evers et al[1])**

| First authors / Checklist items[a] | Black[2] | Brown[3] | Castillo-Requilme[4]; Javitt[5]; Lee[6]; Rothchild[7]; Wongwai[8] | Dave[9] | Dunbar[10] | Isaac[11] | Kamholz[12]; Jackson[13] | Kelkar (2017a)[14]; Kelkar (2017b)[15] | Mohammadi[16] | Moitry[17] | Van den Akker-van Merle[18] | Yanowitch[19] | Zin[20] | **Total** |
|---|---|---|---|---|---|---|---|---|---|---|---|---|---|---|
| 1 | + | + | + | + | + | + | - | + | - | + | + | + | + | **16** |
| 2 | + | + | + | + | + | + | + | + | + | + | + | + | + | **19** |
| 3 | + | + | + | + | + | + | + | + | + | + | + | + | + | **19** |
| 4 | + | + | + | + | + | + | + | + | + | + | + | + | + | **19** |
| 5 | + | + | + | + | + | + | + | + | + | + | + | + | + | **19** |
| 6 | + | + | + | + | + | + | + | + | - | + | + | + | + | **18** |
| 7 | + | + | + | + | + | + | + | + | + | + | + | + | + | **19** |
| 8 | + | + | + | + | + | + | + | + | + | + | + | + | + | **19** |
| 9 | + | + | + | + | + | + | + | + | + | + | + | + | + | **19** |
| 10 | + | + | + | + | + | + | + | + | + | + | + | + | + | **18** |
| 11 | + | + | + | + | + | + | + | + | + | + | + | + | + | **19** |
| 12 | + | + | + | + | + | + | + | + | + | + | + | + | + | **19** |
| 13 | + | - | + | + | + | + | + | - | - | - | + | + | + | **14** |
| 14 | - | - | + | - | + | - | + | - | - | + | + | + | - | **11** |
| 15 | + | - | + | - | - | - | + | - | - | + | - | - | + | **10** |
| 16 | + | + | + | + | + | + | + | + | + | + | + | + | + | **19** |
| 17 | + | + | + | + | + | + | + | + | + | + | + | + | + | **19** |
| 18 | + | + | + | + | - | + | + | - | + | + | - | + | + | **15** |

| | | | | | | | | | | | | | | |
|---|---|---|---|---|---|---|---|---|---|---|---|---|---|---|
| 19 | + | + | + | + | + | + | + | - | + | + | + | + | + | **17** |
| **Total** | **18** | **16** | **19** | **17** | **17** | **17** | **18** | **14** | **14** | **18** | **17** | **17** | **18** | |

[a] Item numbering (also in eTable 2): 1. Is the study population clearly described?; 2. Are competing alternatives clearly described?; 3. Is a well-defined research question posed in answerable form?; 4. Is the economic study design appropriate to the stated objective?; 5. Is the chosen time horizon appropriate in order to include relevant costs and consequences?; 6. Is the actual perspective chosen appropriate?; 7. Are all important and relevant costs for each alternative identified?; 8. Are all costs measured appropriately in physical units?; 9. Are costs valued appropriately?; 10. Are all important and relevant outcomes for each alternative identified?; 11. Are all outcomes measured appropriately?; 12. Are outcomes valued appropriately?; 13. Is an incremental analysis of costs and outcomes of alternatives performed?; 14. Are all future costs and outcomes discounted appropriately?; 15. Are all important variables, whose values are uncertain, appropriately subjected to sensitivity analysis?; 16. Do the conclusions follow from the data reported?; 17. Does the study discuss the generalizability of the results to other settings and patient/client groups?; 18. Does the article indicate that there is no potential conflict of interest of study researcher(s) and funder(s)?; 19. Are ethical and distributional issues discussed appropriately?

**eFigure 1. Flow diagram shows the study selection process, following the PRISMA guidelines.[21]**

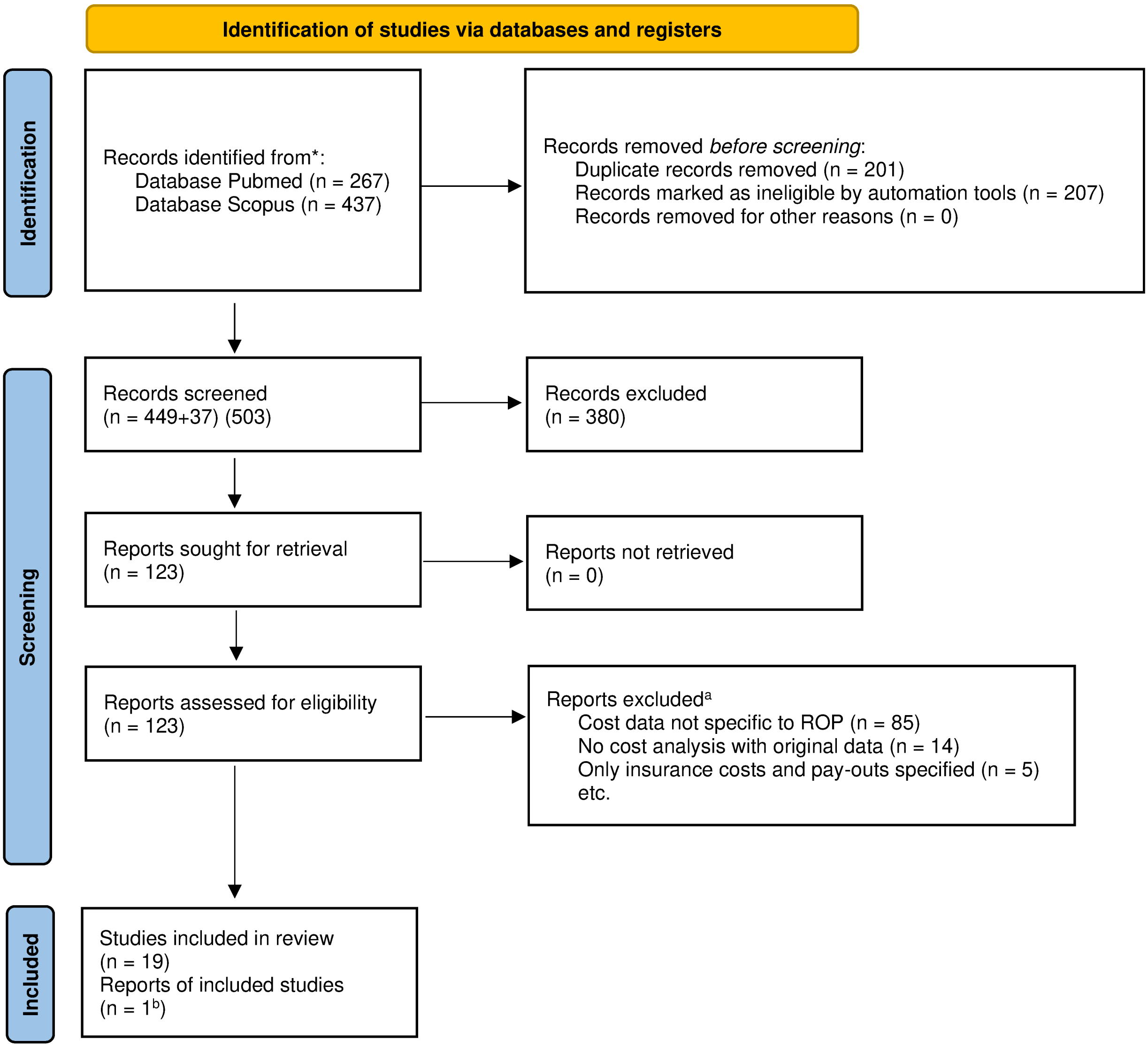


8

[a] For detailed reasons for exclusion of studies that might appear to meet the inclusion criteria, but which were excluded, see also eTable 4.

[b] One author[8] was contacted and clarified the currency of reported results. Another author[16] was unsuccessfully contacted to clarify cost perspective.

Abbreviations: ROP = Retinopathy of prematurity.

**eTable 4. Excluded articles[a]**

| Study | Reason for exclusion |
|---|---|
| Cross 1973. Cost of preventing retrolental fibroplasia?[22] | No original cost data. |
| Boncz et al., 2013. [Health-economic analysis of diseases related to disturbed neonatal adaptation: A cost of illness study].[23] | Only insurance payouts. |
| Yo et al., 2018. Retinopathy of prematurity: the high cost of screening regional and remote infants.[24] | Transport costs but no screening costs. |
| Scholz and Greiner, 2019. An exclusive human milk diet for very low birth weight newborns-A cost-effectiveness and EVPI study for Germany.[25] | No ROP specific costs. |
| Zupancic et al., 2020. Evaluation of the economic impact of modified screening criteria for retinopathy of prematurity from the Postnatal Growth and ROP (G-ROP) study.[26] | No original cost data. |

[a] In this table are listed studies that might appear to meet the inclusion criteria, but which were excluded, and why they were excluded.

Abbreviations: ROP = Retinopathy of prematurity.

## eFigure 2. Cost model

This figure presents our preliminary suggestions for a conceptual model for costs associated with retinopathy of prematurity (ROP), with some additional comments we believe are relevant. Abbreviations: GA=gestational age; ROP=retinopathy of prematurity; VEGF=vascular endothelial growth factor.

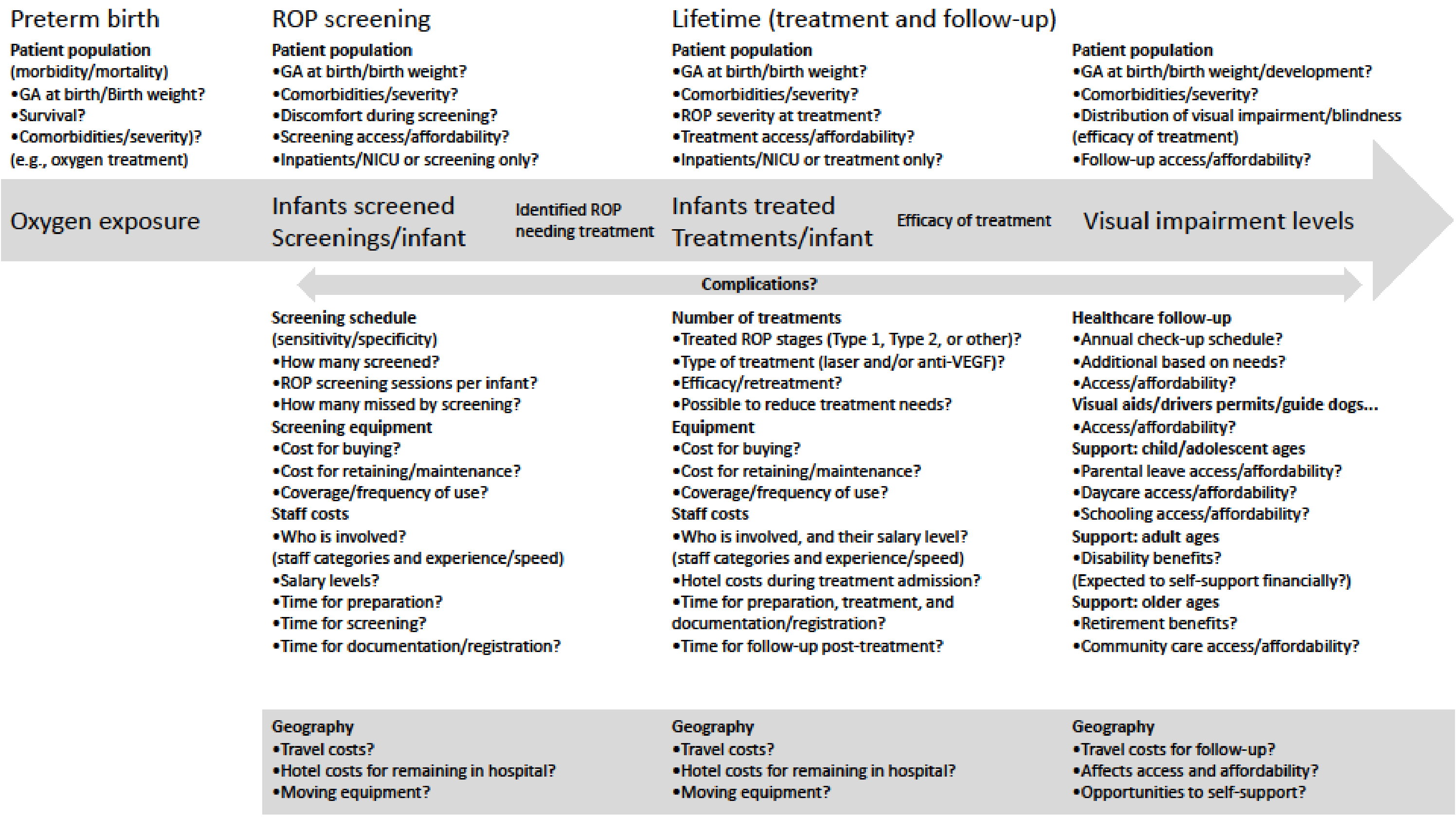

**Preterm birth**

It should be noted that these costs are part of a larger picture of understanding the economic impact of prematurity, which is essential knowledge in predicting the costs and consequences of introducing new interventions that affect gestational age at birth or morbidity and mortality among preterm infants. Thus, the model here is only one part and should be complemented by factors related to, e.g., bronchopulmonary dysplasia and other lung diseases, as well as other neuropsychiatric conditions. The listed items add to the previously published compartmental model of the global burden of ROP,[27] which also accounts for e.g., availability and coverage of screening programs.

**ROP screening**

Some evidence suggests that screening can be reduced even as infants are still identified with high sensitivity and specificity.[5] Reduced screening can be achieved through either changing the frequency of screening or limiting who is actually screened. Based on register findings in Sweden, infants born after gestational week 30 are no longer routinely screened for ROP.[28] Similarly, a study from the Netherlands found no severe ROP among infants born ≥30 gestational weeks.[29] This pattern differs from the situation in many other parts of the world. However, infants born at lower gestational age are more likely to develop ROP and severe ROP.[30]

Costs for screening in the studies included staff salaries/time, equipment and maintenance, supplies, and staff training. Although the identified studies do not detail the cost components and their associated costs, it can be expected that the reported costs of screening are to some extent underestimated. In time-and-motion studies conducted in our local hospital during a process of developing services (unpublished results), the times spent for preparatory work and documentation of screening results were 7–15 minutes and 7–12 minutes, respectively. This range included the time needed to identify infants who should be screened from those born at the facility, but excluded the time used for the actual screening. The figures can be compared to numbers provided in, e.g., Wongwai et al.,[8] citing 10 minutes used for screening by the ophthalmologist and 60 minutes for the nurse. According to Jackson et al.,[13] an average five examinations were necessary for determining if one infant would require treatment for ROP, which is in line with experiences in our hospital.

Regardless of the setting, there will also be transportation costs associated with screening. In this review, we excluded transportation costs, which are highly specific to each setting. For example, an Australian study reported flights for ROP screening to average 36–75 minutes depending on the healthcare center.[24] Transportation can thus include the time and expenses to the families coming into the hospital (or to visit a telemedicine center), or moving within the hospital if the infant remains hospitalized, but they can also reflect the cost of a specialized physician and assistant nurse or other staff category moving within or between hospitals to conduct screening. In addition to being an important cost component to consider in evaluations, the transportation aspect and hotel costs for staying in the hospital can directly affect screening. Our group has clinical experience of parents selecting not to attend planned screening visits after leaving the hospital, so that travel costs also become an issue related to increasing screening adherence and motivating attendance.

**Lifetime (treatment and follow-up)**

Treatment costs in individual studies included, e.g., staff salaries/time, equipment and maintenance, supplies, and staff training. Few studies reported detailed data on cost components, but Wongwai et al.,[8] for example, reported post-screening resource use of 60 minutes for an expert ophthalmologist, which we interpret to be the cost for treatment. Although case-mix and survival of extremely preterm infants were not detailed in the included studies, it can be expected that these factors will affect how many infants need treatment for ROP. For example, among infants born ≤30 gestational weeks in Sweden, 32% had any stage ROP and 6% were treated for ROP,[28] but among infants born at <24 gestational weeks, the corresponding figures were 92% and 43%.[31] Moreover, the available treatment options would affect costs, with studies suggesting, e.g., more retreatments with the more recent anti-vascular endothelial growth factor (VEGF) therapy.[28] Surgical intervention, or vitrectomy, could also apply to more severe cases,[32] in particular in countries with low access to screening. Although the costs of vitrectomy itself appear to be low,[33] there are likely other costs associated with these severe ROP cases, such as those linked to follow-up and complications.[34]

The argument regarding transportation costs is highly relevant for the treatment of ROP. The clinical reality of many countries is that patients

must be flown to the treatment site, or undergo multiple relocations by ambulance between local hospitals and specialized units providing the treatment.

At least in countries with high access to healthcare, it can be expected that children with ROP, and particularly those with severe forms requiring treatment, will have multiple follow-ups during childhood, adolescence, and possibly into adulthood. The low number of healthcare visits for follow-up indicated in the included articles differs considerably from the national guidelines in Sweden, recommending annual follow-up of ROP until adulthood and, after that, according to need. In a recent publication reporting on a model for predicting visual outcomes after ROP treatment,[35] follow-up every 6 months was even indicated for some patient groups.

Although costs for blindness can be expected to be similar regardless of the cause of blindness, data are available on approximate cost levels for different levels of visual impairment.[36] Thus, tapping into models for measuring costs of visual impairment can add to understanding of the long-term consequences of ROP.

## References

1. Evers S, Goossens M, de Vet H, van Tulder M, Ament A. Criteria list for assessment of methodological quality of economic evaluations: Consensus on Health Economic Criteria. *Int J Technol Assess Health Care*. 2005;21(2):240-245.

2. Black L, Hulsey T, Lee K, Parks DC, Ebeling MD. Incremental Hospital Costs Associated With Comorbidities of Prematurity. *Manag Care*. 2015;24(12):54-60.

3. Brown GC, Brown MM, Sharma S, Tasman W, Brown HC. Cost-effectiveness of treatment for threshold retinopathy of prematurity. *Pediatrics*. 1999;104(4):e47.

4. Castillo-Riquelme MC, Lord J, Moseley MJ, Fielder AR, Haines L. Cost-effectiveness of digital photographic screening for retinopathy of prematurity in the United Kingdom. *Int J Technol Assess Health Care*. 2004;20(2):201-213.

5. Javitt J, Cas RD, Chiang YP. Cost-effectiveness of screening and cryotherapy for threshold retinopathy of prematurity. *Pediatrics*. 1993;91(5):859-866.

6. Lee SK, Norm, C., et al. Evidence for changing guidelines for routine screening for retinopathy of prematurity. *Arch Pediatr Adolesc Med*. 2001;155(3):387-395.

7. Rothschild MI, Russ R, Brennan KA, et al. The Economic Model of Retinopathy of Prematurity (EcROP) Screening and Treatment: Mexico and the United States. *Am J Ophthalmol*. 2016;168:110-121.

8. Wongwai P, Kingkaew P, Asawaphureekorn S, Kolatat T. A store-and-forward telemedicine for retinopathy of prematurity screen: Is it cost-effective in Thailand? *Asian Biomedicine*. 2015;9(5):665-673.

9. Dave HB, Gordillo L, Yang Z, Zhang MS, Hubbard GB 3rd, Olsen TW. The societal burden of blindness secondary to retinopathy of prematurity in Lima, Peru. *Am J Ophthalmol*. 2012;154(4):750-755.

10. Dunbar JA, Hsu V, Christensen M, Black B, Williams P, Beauchamp G. Cost-utility analysis of screening and laser treatment of retinopathy of prematurity. *Journal of AAPOS*. 2009;13(2):186-190.

11. Isaac M, Isaranuwatchai W, Tehrani N. Cost analysis of remote telemedicine screening for retinopathy of prematurity. *Can J Ophthalmol*. 2018;53(2):162-167.

12. Kamholz KL, Cole CH, Gray JE, Zupancic JA. Cost-effectiveness of early treatment for retinopathy of prematurity. *Pediatrics*. 2009;123(1):262-269.

13. Jackson KM, Scott KE, Graff Zivin J, et al. Cost-utility analysis of telemedicine and ophthalmoscopy for retinopathy of prematurity management. *Arch Ophthalmol*. 2008;126(4):493-499.

14. Kelkar J, Agashe S, Kelkar A, Kh, ekar R. Mobile unit for retinopathy of prematurity screening and management at urban Neonatal Intensive Care Units: Outcomes and impact assessment. *Oman J Ophthalmol*. 2017;10(1):13-16.

15. Kelkar J, Kelkar A, Sharma S, Dewani J. A mobile team for screening of retinopathy of prematurity in India: Cost - effectiveness, outcomes, and impact assessment. *Taiwan J Ophthalmol*. 2017;7(3):155-159.

16. Mohammadi S-F, Rahban A, Darabeigi S, et al. Cost-effectiveness analysis of tele-retinopathy of prematurity screening in Iran. *Int J Ophthalmol*. 2021;14(4):560-566. doi:10.18240/ijo.2021.04.13

17. Moitry M, Zarca K, Granier M, et al. Effectiveness and efficiency of tele-expertise for improving access to retinopathy screening among 351 neonates in a secondary care center: An observational, controlled before-after study. *PLoS One*. 2018;13(10):e0206375.

18. van den Akker-van Marle ME, van Sorge AJ, Schalij-Delfos NE. Cost and effects of risk factor guided screening strategies for retinopathy of prematurity for different treatment strategies. *Acta Ophthalmol*. 2015;93(8):706-712.

19. Yanovitch TL, Siatkowski RM, McCaffree M, Corff KE. Retinopathy of prematurity in infants with birth weight>or=1250 grams-incidence, severity, and screening guideline cost-analysis. *J aapos*. 2006;10(2):128-134.

20. Zin AA, Magluta C, Pinto MF, et al. Retinopathy of prematurity screening and treatment cost in Brazil. *Rev Panam Salud Publica*. 2014;36(1):37-43.

21. Page MJ, McKenzie JE, Bossuyt PM, et al. The PRISMA 2020 statement: an updated guideline for reporting systematic reviews. *BMJ*. 2021;372:n71. doi:10.1136/bmj.n71

22. Cross KW. Cost of preventing retrolental fibroplasia? *Lancet*. 1973;2(7835):954-956. doi:10.1016/s0140-6736(73)92610-x

23. Boncz I, Kovács LG, Ertl T, Ágoston I, Molics B, Bódis J. Health-economic analysis of diseases related to disturbed neonatal adaptation: A cost of illness study. *Lege Artis Medicinae*. 2013;23(3-4):193-197.

24. Yu T-Y, Donovan T, Armfield N, Gole GA. Retinopathy of prematurity: the high cost of screening regional and remote infants. *Clin Exp Ophthalmol*. 2018;46(6):645-651. doi:10.1111/ceo.13160

25. Scholz SM, Greiner W. An exclusive human milk diet for very low birth weight newborns-A cost-effectiveness and EVPI study for Germany. *PLoS One*. 2019;14(12):e0226496. doi:10.1371/journal.pone.0226496

26. Zupancic JAF, Ying G-S, de Alba Campomanes A, Tomlinson LA, Binenbaum G, G-ROP Study Group. Evaluation of the economic impact of modified screening criteria for retinopathy of prematurity from the Postnatal Growth and ROP (G-ROP) study. *J Perinatol*. 2020;40(7):1100-1108. doi:10.1038/s41372-020-0605-5

27. Blencowe H, Lawn JE, Vazquez T, Fielder A, Gilbert C. Preterm-associated visual impairment and estimates of retinopathy of prematurity at regional and global levels for 2010. *Pediatr Res*. 2013;74(Suppl 1):35-49. doi:10.1038/pr.2013.205

28. Holmström G, Hellström A, Gränse L, et al. New modifications of Swedish ROP guidelines based on 10-year data from the SWEDROP register. *Br J Ophthalmol*. 2020;104(7):943-949. doi:10.1136/bjophthalmol-2019-314874

29. Trzcionkowska K, Groenendaal F, Andriessen P, et al. Risk Factors for Retinopathy of Prematurity in the Netherlands: A Comparison of Two Cohorts. *Neonatology*. 2021;118(4):462-469. doi:10.1159/000517247

30. Isaza G, Arora S. Incidence and severity of retinopathy of prematurity in extremely premature infants. *Canadian Journal of Ophthalmology*. 2012;47(3):296-300. doi:10.1016/j.jcjo.2012.03.027

31. Lundgren P, Jacobson L, Hård A-L, et al. High rate and large intercentre variability in retreatment of retinopathy of prematurity in infants born <24 gestational weeks. *BMJ Open Ophthalmol*. 2021;6(1):e000695. doi:10.1136/bmjophth-2020-000695

32. Rajan RP, Kannan NB, Sen S, et al. Clinico-demographic profile and outcomes of 25-gauge vitrectomy in advanced stage 5 retinopathy of prematurity. *Graefe's Archive for Clinical and Experimental Ophthalmology*. 2021;259(7):1695-1701. doi:10.1007/s00417-020-05063-2

33. Nicod E, Jackson TL, Grimaccia F, et al. Direct cost of pars plana vitrectomy for the treatment of macular hole, epiretinal membrane and vitreomacular traction: a bottom-up approach. *European Journal of Health Economics*. 2016;17(8):991-999. doi:10.1007/s10198-015-0741-6

34. Zhao D-Y, Zhang Y-J, He Z-J, et al. Clinical analysis of apnea after operation for retinopathy of prematurity. *Journal of Shanghai Jiaotong University (Medical Science)*. 2010;30(2):132-134.

35. Huang C-Y, Kuo R-J, Li C-H, et al. Prediction of visual outcomes by an artificial neural network following intravitreal injection and laser therapy for retinopathy of prematurity. *British Journal of Ophthalmology*. 2020;104(9):1277-1282. doi:10.1136/bjophthalmol-2019-314860

36. Köberlein J, Beifus K, Schaffert C, Finger RP. The economic burden of visual impairment and blindness: a systematic review. *BMJ Open*. 2013;3(11):e003471. doi:10.1136/bmjopen-2013-003471
