# Supplementary material for: Costs associated with retinopathy of prematurity: a systematic review and meta-analysis
Source: BMJ Open. 2022 Nov 23;12(11):e057864. doi: 10.1136/bmjopen-2021-057864 (PMC9693652; doi:10.1136/bmjopen-2021-057864)
Supplement: Author's manuscript [file bmjopen-2021-057864.draft_revisions.pdf]

BMJ Open is committed to open peer review. As part of this commitment we make the peer review history of every article we publish publicly available.

When an article is published we post the peer reviewers' comments and the authors' responses online. We also post the versions of the paper that were used during peer review. These are the versions that the peer review comments apply to.

The versions of the paper that follow are the versions that were submitted during the peer review process. They are not the versions of record or the final published versions. They should not be cited or distributed as the published version of this manuscript.

BMJ Open is an open access journal and the full, final, typeset and author-corrected version of record of the manuscript is available on our site with no access controls, subscription charges or pay-per-view fees (http://bmjopen.bmj.com).

If you have any questions on BMJ Open's open peer review process please email info.bmjopen@bmj.com

Costs Associated with Retinopathy of Prematurity: A Systematic Review and Meta-analysis

Journal:	*BMJ Open*
Manuscript ID	bmjopen-2021-057864
Article Type:	Original research
Date Submitted by the Author:	29-Sep-2021

Complete List of Authors:	Gyllensten, Hanna; University of Gothenburg Institute of Health and Care Sciences; University of Gothenburg Centre for Person-Centred Care<br>Sjöbom, Ulrika; University of Gothenburg Institute of Health and Care Sciences; University of Gothenburg, Department of Clinical Neuroscience<br>Humayun, Jhangir; University of Gothenburg Institute of Health and Care Sciences; University of Gothenburg Centre for Person-Centred Care<br>Hellström, Ann; University of Gothenburg Institute of Neuroscience and Physiology, Neuroscience<br>Löfqvist, Chatarina; University of Gothenburg Institute of Health and Care Sciences, Sahlgrenska Academy; University of Gothenburg Institute of Neuroscience and Physiology, Department of Clinical Neuroscience

Keywords:	HEALTH ECONOMICS, Paediatric ophthalmology < OPHTHALMOLOGY, Medical retina < OPHTHALMOLOGY

I, the Submitting Author has the right to grant and does grant on behalf of all authors of the Work (as defined in the below author licence), an exclusive licence and/or a non-exclusive licence for contributions from authors who are: i) UK Crown employees; ii) where BMJ has agreed a CC-BY licence shall apply, and/or iii) in accordance with the terms applicable for US Federal Government officers or employees acting as part of their official duties; on a worldwide, perpetual, irrevocable, royalty-free basis to BMJ Publishing Group Ltd ("BMJ") its licensees and where the relevant Journal is co-owned by BMJ to the co-owners of the Journal, to publish the Work in this journal and any other BMJ products and to exploit all rights, as set out in our licence.

The Submitting Author accepts and understands that any supply made under these terms is made by BMJ to the Submitting Author unless you are acting as an employee on behalf of your employer or a postgraduate student of an affiliated institution which is paying any applicable article publishing charge ("APC") for Open Access articles. Where the Submitting Author wishes to make the Work available on an Open Access basis (and intends to pay the relevant APC), the terms of reuse of such Open Access shall be governed by a Creative Commons licence – details of these licences and which Creative Commons licence will apply to this Work are set out in our licence referred to above.

Other than as permitted in any relevant BMJ Author's Self Archiving Policies, I confirm this Work has not been accepted for publication elsewhere, is not being considered for publication elsewhere and does not duplicate material already published. I confirm all authors consent to publication of this Work and authorise the granting of this licence.

For peer review only

Title page

Costs Associated with Retinopathy of Prematurity: A Systematic Review and Meta-analysis

Authors

Hanna Gyllensten[1,2] Associate professor

Jhangir Humayun[1,2], B.Sc.

Ulrika Sjöbom[1,3], M.Sc.

Ann Hellström[3] Professor

Chatarina Löfqvist[1,2,3] Associate professor

Author affiliations

[1] Institute of Health and Care Sciences, Sahlgrenska Academy, University of Gothenburg, Box 457, SE-405 30 Gothenburg, Sweden.

[2] University of Gothenburg Centre for Person-Centred Care (GPCC), Sahlgrenska Academy, University of Gothenburg, Box 457, SE-405 30 Gothenburg, Sweden.

[3] Department of Clinical Neuroscience, Institute of Neuroscience and Physiology, Sahlgrenska Academy, University of Gothenburg, Gothenburg, Sweden;

Contact, corresponding author

Hanna Gyllensten, Institute of Health and Care Sciences, Sahlgrenska Academy, University of Gothenburg, Postal Address: Box 457, SE-405 30 Gothenburg, Sweden, E-mail: hanna.gyllensten@gu.se. Phone: +46-(0)70-748 24 12

Keywords

Retinopathy of Prematurity; Costs and Cost Analysis; Systematic Review; Meta-Analysis.

Word count (excluding title page, abstract, references, figures and tables): 2644

For peer review only - http://bmjopen.bmj.com/site/about/guidelines.xhtml

Abstract

Objective: To review and analyze evidence regarding costs for ROP screening, lifetime costs and resource use among infants born preterm who develop ROP, and how these costs have developed over time in different regions.

Design: Systematic literature review and meta-analysis.

Setting: PubMed and Scopus from inception to June 23, 2021.

Participants: Included studies presented the full cost or cost increase associated with ROP screening and treatment. Studies not reporting on cost calculation methods or ROP-specific costs were excluded. Included studies were further searched to identify eligible references and citations. Two independent reviewers assessed studies for inclusion or exclusion. Following pre-determined specifications, they extracted the data from the selected publications, including items from a published checklist for quality assessment, summary, and meta-analysis for treatment costs.

Primary and secondary outcome measures: Main outcomes were costs for ROP screening and the lifetime costs (including laser treatment and follow-up costs) and resource use among people who develop ROP.

Results: In total, 15 studies reported ROP screening costs, and 13 reported lifetime costs (either treatment and/or follow-up costs) for infants with ROP. The range for screening costs (10 studies) was US$5–$253 per visit, or US$324–$1072 per screened child (5 studies). Costs for treatment (11 studies) ranged from US$38 to US$6500 per child. Four studies reported healthcare follow-up costs (lifetime costs ranging from US$64–US$2420, and 10 year-costs of US$1695, respectively), and of these, three also reported lifetime costs for blindness (range US$26,686–US$224,295) using secondary cost data.

For peer review only - http://bmjopen.bmj.com/site/about/guidelines.xhtml

Conclusions: The costs of screening for and treating ROP are small compared to the potential societal costs of resulting blindness. Little evidence is available for predicting the effects of changes in patient population, screening schedule, or ROP treatments.

Registration number in PROSPERO: CRD42020208213

Strengths and limitations of this study

- To our knowledge, this is the first systematic review or meta-analysis of Retinopathy of Prematurity costs.
- PubMed and Scopus were searched systematically, and manual search of reference lists and citations of the identified papers did not identify any additional studies, thus indicating that the database search had good coverage of the topic of investigation.
- The main limitations of this work were the exclusion of grey literature and the lack of analyses of publication bias for the meta-analysis.

For peer review only

Introduction

Improvements in neonatal care have resulted in increased survival among children born preterm,[1] but these infants are at risk of developing preterm-related morbidities such as retinopathy of prematurity (ROP). ROP is characterized by abnormal neurovascular development and, in its worst forms, retinal detachment and blindness.[2] Although preventable, ROP is the leading cause of blindness worldwide,[3] a ranking associated with the survival of infants with extremely low gestational age and birth weight in some parts of the world, and use of unmonitored treatments with 100% oxygen in other regions.[2]

ROP management and treatment economics are still challenging in many health systems because of screening-associated costs, patient-related costs, and medico-legal liability.[4] Thus, there is an urgent need for more concerted efforts to guide healthcare providers in how to use resources efficiently, both in developing economies during a phase of improving survival of preterm infants[5] and in countries with major neonatal morbidities still affecting a large proportion of those who survive.[6]

Here we present an overview of costs associated with ROP screening and treatment, examining the evidence related to costs for ROP screening and lifetime costs (including laser treatment and follow-up costs) and resource use among infants born preterm who develop ROP. We also examine the trajectories of these costs over time in different regions in a meta-analysis.

Methods

This work followed the Preferred Reporting Items for Systematic Review and Meta-Analysis (i.e., PRISMA),[7] with protocol available in PROSPERO (reference CRD42020208213).[8]

For peer review only - http://bmjopen.bmj.com/site/about/guidelines.xhtml

Article search

Pubmed and Scopus were searched (eTable 1, 23 Jun 2021) to identify original research on costs for ROP, including full cost or cost increases associated with ROP, without restricting language, publication date, or country. Articles that did not describe the cost calculation method were excluded, as were those not presenting the costs for the group with ROP separately.

Rayyan QCRI was used for handling duplicates and the selection of studies for inclusion. Two independent reviewers (JH and CL or HG) searched the databases, screened articles for eligibility, extracted data using a pre-specified data extraction sheet (eTable 2), and hand-searched included studies (7 July 2021) to identify eligible references and citations. Conflicting views were resolved by discussion with a third reviewer (CL or HG). The data extraction sheet included items from a published checklist for quality assessment of economic evaluations[9] to assess the risk of bias in included studies.

Analysis

Conventional screening (excluding telemedicine costs), laser treatment, and long-term follow-up costs were reported, respectively, accounting for ROP severity and differences over time and between countries. Identified costs were adjusted to 2020 US dollars (US$) using annual exchange rates[10] and the Organisation for Economic Co-operation and Development inflation factor.[11] After imputation of missing variance, treatment costs were summarized in a forest plot , by year and subgroups using country classification,[12] as cost levels can be expected to differ.

For peer review only - http://bmjopen.bmj.com/site/about/guidelines.xhtml

Patient and public involvement

This project did not include patient or public involvement in developing the research questions, design, conduct, choice of outcome measures, or recruitment.

Results

Of the 503 studies screened after duplicates from the databases were removed, 123 were assessed for eligibility based on full text, and 19 studies were included in the synthesis of results (eFigure 1). Reasons for exclusion were absence of data on costs associated with ROP, lack of original data, or inclusion of data related only to insurance payments or litigation. No additional studies were identified by a hand search of references and a Scopus search of citations of included studies. An overview of all included studies[13–31] is presented in Table 1, including references to secondary cost sources.[32–38] In total, 15 studies covered screening costs and 13 reported lifetime costs (treatment and/or follow-up costs) for infants who developed ROP.

Twelve studies were conducted in high-income economies: seven in the United States, two in Canada, and one each in the United Kingdom, Netherlands, and France. Three studies were conducted in upper-middle income economies: one each in Peru, Thailand, and Brazil. Three studies were conducted in lower-middle income economies: two in India and one in Iran. One study was conducted in both the United States and Mexico (Table 1). All studies reported the economic analyses using either US dollars, euros, or local currency. The patient populations in all studies were infants at risk for ROP, although the studies used different inclusion criteria based on gestational age at birth and birth weight. In addition, the ROP definition for stages and treatment criteria varied with the timing of the study and international guidelines for classification at that time.

For peer review only - http://bmjopen.bmj.com/site/about/guidelines.xhtml

Risk of bias in included studies

The quality assessment indicated a high overall quality of the included studies (eTable 3), with 16 of 19 of them fulfilling at least 16 of the assessed criteria. However, eight studies did not fulfill the criteria for discounting future costs and outcomes or for subjecting results to sensitivity analyses to address the effects of assumptions. Additionally, 14 studies met criteria regarding the reporting of incremental analysis and potential conflicts of interest. Thus, overall, the assessment suggested a low risk of bias in the included papers, and also indicated where lack of reporting on potential conflicts of interest was most problematic. Quality of evidence was rated on a scale from 1 to 5 for individual articles, with articles most commonly based on data from retrospective cohort studies (evidence rating 3; 9 publications). Few of the included articles reported disaggregated cost and resource use data or detailed the included cost components, as is recommended for economic evaluations.[39]

Costs for ROP screening

Studies reporting costs related to screening had different designs: six were retrospective cohort studies using medical chart review or register data,[14,15,19,23,27,29] nine developed economic models,[18,20,22,24–26,28,30,31] and two were public intervention studies related to the introduction of ROP screening programs.[16,17] Although the assessment indicated a low risk of bias, screening costs differed substantially among reporting countries (Figure 1a).

Costs for routine ROP screening, excluding transportation costs, are reported in Table 2. Ten studies reported a mean unit cost per screening of US$137 (range: 5–253). In addition, five studies reported a mean cost per screened child of US$553 (range: 324–1072). Of these, two studies reported comparably low costs[20,22] for staff and equipment, whereas Rothchild et al.[18] reported comparably higher costs in the US setting. One study also included transportation costs,[14] and when these costs were removed, screening cost was comparably

For peer review only - http://bmjopen.bmj.com/site/about/guidelines.xhtml

low. The other studies reported similar costs for screening per child (range: US$324–$602).[24,27,28]

Javitt et al.[31] reported a mean unit cost of US$183 for a first screening and of US$149 for follow-up screening, whereas Lee et al.[29] reported a mean unit cost of US$112 for screening one eye. Finally, two studies from India[16,17] reported screening costs of US$1003 and US$630, respectively, for identifying one child with ROP.

In studies comparing alternative screening or treatment options, no common comparator was identified. The incremental cost reported in Black et al.[21] indicated a savings associated with higher gestational age at birth (Table 1). Jackson et al.[26] used economic modeling to estimate the cost-utility of ROP screening using telemedicine vs. conventional ROP screening. Javitt et al.[31] used modeling to compare weekly, biweekly, or monthly screening.

Costs for ROP treatment

In all, 14 studies reported costs related to the laser treatment of ROP (Figure 1b). Four studies of treatment costs were retrospective cohort studies,[19,23,27,29] eight were modeling studies,[13,18,20,22,24,25,28,30] and two were public intervention studies.[16,17] In addition, two of the included studies[30,31] reported costs for cryotherapy (not included in the analyses below). Eleven studies reported total treatment costs per child, at a mean US$2442 (range: 38–6500). Castillo-Riquelme et al.[28] found unilateral treatment costs up to US$1165 and bilateral treatment costs up to US$1514, based partially on secondary data from Brown et al.[30] Two studies[19,25] cited unit costs of laser treatment of US$4065 and US$5661, respectively. Laser treatment costs, excluding transportation costs, are reported in Table 2. Dave et al.[23] described costs for screening and treatment combined (US$2962) in a cohort of children with blindness.

For peer review only - http://bmjopen.bmj.com/site/about/guidelines.xhtml

Accounting for the low assessed risk of bias but large expected variation based on cost-levels of individual countries, the meta-analysis by country classification (Figures 2-3) estimated the average costs in high-income economies to US$2960 (95% confidence interval [CI]: 2003–3917). Corresponding figures were US$329 (95% CI: 9–649) in upper-middle–income economies and US$3692 (95% CI: 670–6715) in lower-middle–income economies, respectively. Most studies did not report variance of results, making publication bias analysis unfeasible. However, model diagnostics (I^2 and Cochrane Q) indicated high heterogeneity between studies within each country classification, which suggests that the results from the meta-analysis should be interpreted with caution.

Follow-up costs and resource use among infants born preterm and developing ROP

Only four studies reported follow-up costs occurring after screening and treatment, and although the risk of bias was assessed as low, the reported results largely differed between studies. Castillo-Riquelme et al.[28] reported healthcare follow-up costs over 10 years of up to US$1695. Dave et al.[23] reported a lifetime follow-up visit cost of US$64 and a blindness cost of US$146,952. Rothchild et al.[18] reported lifetime follow-up healthcare costs of US$1681 (US) and US$2420 (Mexico), whereas the costs for blindness were estimated to be US$92,460 (US) and US$26,686 (Mexico). Wongwai et al.[20] reported the lifetime costs of blindness to be $224,295. In addition, Black et al.[21] reported the costs per quality-adjusted life-year (QALY) associated with ROP and other comorbidities associated with being born preterm.

Discussion

The studies we identified could be grouped by whether they reported costs for screening, costs for treatment, or costs (and QALYs) during long-term follow-up or even from a lifetime

For peer review only - http://bmjopen.bmj.com/site/about/guidelines.xhtml

perspective. The cost range per ROP screening was US$5–$253 per visit, or US$324–$1072 per screened child. Costs for ROP treatment ranged from US$38–$6500 per child. In addition, four studies reported healthcare follow-up costs, and three reported lifetime costs using secondary data on costs for blindness. Although quality assessment indicated a low risk of bias, comparisons between studies were challenging because of the lack of detailed cost and resource use data.

To our knowledge, this is the first systematic review of ROP costs. Included papers largely followed the quality assessment checklist items of a commonly used tool,[40] thus indicating a low risk of bias. The main limitations of this work were the exclusion of grey literature and the lack of analyses of publication bias for the meta-analysis. Guidance for reliability in systematic reviews of retinal disorder interventions[41] was fulfilled, but the standards for systematic reviews of costs and cost-effectiveness studies were not.[42] Moreover, the search strategy and databases are expected to cover largely English-language literature, but as the reference and citation search yielded no additional studies to include, we expect our findings to represent a good overview of the available evidence.

Cost components for ROP screening included staff salaries/time, equipment and maintenance, supplies, and staff training. Screening costs for ROP were low compared to other associated costs and, with few exceptions, of the same order of magnitude in the included studies. Exceptions were probably attributable to salary differences.

Screening access and schedules vary between countries.[43] With the possible exception of Javitt et al.,[31], the included studies provided little evidence for how case-mix and alternative screening schedules affect costs for screening. Savings are expected, however, and a modeling study using published cost data calculated an annual cost savings from reduced screening of US$3 million in the United States.[44] However, with low screening costs, the main benefit is reduced discomfort for the infants and reduced travel costs (which can be

For peer review only - http://bmjopen.bmj.com/site/about/guidelines.xhtml
For peer review only

substantial[14]). The most considerable potential for savings on screening is probably increasing gestational age. US data indicate that ROP frequency increased over time, particularly in infants born very preterm,[45] and infants of lower gestational age usually both require more screening visits and have more severe ROP.[46] Potential savings have been reported from screening using telemedicine (compared to transporting infants to a specialized hospital),[14] or using bedside screening with mobile equipment instead of moving the infants to a specific screening facility[47]; however, this review did not consider these aspects.

Treatment costs were low compared to the costs for follow-up, with Brazil, Mexico, and Peru having substantially lower treatment costs than the other countries. Both Javitt et al.[31] and Brown et al.[30] reported low costs for the historically used cryo treatment, at approximately 63% of that for laser treatment. For laser treatment, the cost range was US$2304–$6864 per treated child. None of the studies included the more recent anti-vascular endothelial growth factor (VEGF) therapy. Moreover, no study reported costs based on ROP stages, age of treated infants, or plus disease status.[48] Thus, studies provide little guidance on how treatment costs will develop over time as more infants of lower gestational age survive.

Variation among studies in whether one or two eyes were treated made comparisons less relevant, which may reflect the unilateral schedule used in the historically influential Cryo-ROP study.[49] However, Swedish registers indicate that bilateral treatment is common (76% of initial treatments and 97% overall)[46] and that retreatment is more frequent among infants with very low gestational age[50] and those treated exclusively with anti-VEGF.[46]

Cost components for ROP treatment included staff salaries/time, equipment and maintenance, supplies, and staff training. Sometimes anesthesia costs were reported separately or excluded. Transportation was also a considerable cost component in relation to treatment.[19] Other potential costs that were not measured include those for the added time spent in hospital or intensive care, including parental leave, during treatment. Many studies

For peer review only - http://bmjopen.bmj.com/site/about/guidelines.xhtml

reported only total charges, which are expected to be higher than costs to the healthcare provider. However, use of charges as opposed to costs was not an obvious cause of variation here. Two studies from India[16,17] reported high costs compared to other studies of both costs and charges, possibly because of some transportation costs remaining as part of additional components.

Although ROP results in high costs throughout life, this outcome is primarily based on secondary data for blindness. As the leading cause of preventable childhood blindness[51] and probably the leading cause of childhood blindness in middle-income countries,[52] ROP should be associated with much of the estimated costs of blindness. Moreover, it has been argued that costs for blindness do not differ by cause.[53] Little evidence was available on follow-up after successful, or partially successful, treatment of ROP. Dave et al.[23] indicated three healthcare visits over the first 7 years of life, whereas Castillo-Riquelme et al.[28] did not differentiate visits based on treatment or ROP stage. Rothchild et al. included transportation costs, white canes, Braille equipment, and supplies,[18] but disregarded other costs among children retaining sight. Thus, although costs differ by the severity of visual impairment,[54] studies of ROP costs do not tend to report this more detailed level of sight. The current knowledge does not inform potential savings or inform subsidy decisions for ROP treatment developments that can save a little more sight. Taken together, the short follow-up underestimates the total impact of blindness,[55] and not accounting for visual impairment results in underestimating the financial impact of ROP.

There is a need for comprehensive knowledge about the costs of ROP, both during the introduction of new ROP screening programs and in countries with established programs that are now redistributing resources to handle the increasing survival of very preterm infants with high disease burden. In addition to relevant cost components of ROP (eFigure 2), complementary studies of the benefits of various neonatal preventative strategies, including

For peer review only - http://bmjopen.bmj.com/site/about/guidelines.xhtml

oxygen delivery, are warranted because evidence of the costs resulting from conditions such as bronchopulmonary dysplasia is also lacking.[56] Such studies should follow state-of-the-art methods for conduct and reporting of health economic studies.

Conclusions

Although costs of screening and treating ROP are substantial for health systems, they are small compared to the follow-up costs to society of resulting blindness. However, little evidence is available to support predictions about the consequences of changes in the patient population, screening schedule, or treatment regimens for ROP.

For peer review only

FUNDING STATEMENT

HG was financed by the Swedish Research Council (#2016-01131). JH was financed by the University of Gothenburg Centre for Person-Centred Care (GPCC), a teaching assistant program. AH was supported by The Wallenberg Clinical Scholars, The Swedish Research Council (#2020-01092), the Gothenburg County Council (ALF project, #426531), the Gothenburg Medical Society, and De Blindas Vänner, and CL was financed by the GPCC. The funders had no role in the design of the study or writing of the protocol.

Acknowledgments

COMPETING INTERESTS STATEMENT

HG is employed part-time by IQVIA, which is a contract research organization that performs commissioned pharmacoepidemiological studies. Thus, its employees have been and currently are working in collaboration with several pharmaceutical companies. JH reports no competing interests. AH holds stock/stock options in Premalux AB and has received consulting fees from Takeda Inc. CL holds stocks in Premalux AB.

CONTRIBUTIONS

All authors contributed to the design of the study. HG, JH, and CL designed the database search and data extraction methods. JH and CL undertook the literature search, assessed studies for eligibility, and extracted data. In case of disagreement, assessments were made in discussion with HG. AH contributed clinical expertise on preterm infants and morbidity. HG, JH, US, and CL discussed the data and interpreted the results. HG, JH, and CL drafted the manuscript. All authors critically reviewed and approved the final manuscript. HG had full access to all the data in the study and takes responsibility for the integrity of the data and the accuracy of the data analysis.

ACKNOWLEDGEMENTS

Thanks to SF Edit, a professional scientific-editing service, for language editing.

For peer review only - http://bmjopen.bmj.com/site/about/guidelines.xhtml

DATA AVAILABILITY STATEMENT

All data relevant to the study are included in the article. Original data are available in the reviewed publications, which are all cited. Additional data from the data extraction performed are available on reasonable request from the corresponding author, including author template data collection forms, data extracted from included studies, data used for all analyses, analytic code, and any other materials used in the review.

ETHICS APPROVAL STATEMENT

Not applicable.

For peer review only

References

1. Lawn JE, Davidge R, Paul VK, et al. Born too soon: care for the preterm baby. *Reprod Health*. 2013;10 Suppl 1:S5. doi:10.1186/1742-4755-10-S1-S5

2. Hellström A, Smith LEH, Dammann O. Retinopathy of prematurity. *Lancet*. 2013;382(9902):1445-1457. doi:10.1016/S0140-6736(13)60178-6

3. Yonekawa Y, Thomas BJ, Thanos A, et al. THE CUTTING EDGE OF RETINOPATHY OF PREMATURITY CARE: Expanding the Boundaries of Diagnosis and Treatment. *Retina (Philadelphia, Pa)*. 2017;37(12):2208-2225. doi:10.1097/IAE.0000000000001719

4. Moshfeghi DM, Capone A. Economic Barriers in Retinopathy of Prematurity Management. *Ophthalmol Retina*. 2018;2(12):1177-1178. doi:10.1016/j.oret.2018.10.002

5. Dupe S. Ademola-Popoola, Tunji S. Oluleye. Retinopathy of Prematurity (ROP) in a Developing Economy with Improving Health Care | SpringerLink. *Current Ophtalmology Reports*. 2017;5:114-118. doi:https://doi-org.ezproxy.ub.gu.se/10.1007/s40135-017-0129-0

6. Norman M, Hallberg B, Abrahamsson T, et al. Association Between Year of Birth and 1-Year Survival Among Extremely Preterm Infants in Sweden During 2004-2007 and 2014-2016. *JAMA*. 2019;321(12):1188-1199. doi:10.1001/jama.2019.2021

7. Page MJ, McKenzie JE, Bossuyt PM, et al. The PRISMA 2020 statement: an updated guideline for reporting systematic reviews. *BMJ*. 2021;372:n71. doi:10.1136/bmj.n71

8. PROSPERO International prospective register of systematic reviews. NIHR, National INstitute for Health Research. https://www.crd.york.ac.uk/prospero/

9. Evers S, Goossens M, de Vet H, van Tulder M, Ament A. Criteria list for assessment of methodological quality of economic evaluations: Consensus on Health Economic Criteria. *Int J Technol Assess Health Care*. 2005;21(2):240-245.

10. Annual average exchange rates (aggregate). Sveriges Riksbank. Accessed June 8, 2021. https://www.riksbank.se/en-gb/statistics/search-interest--exchange-rates/annual-average-exchange-rates/

11. Prices - Inflation (CPI) - OECD Data. Organisation for Economic Co-operation and Development, OECD. Accessed June 9, 2021. http://data.oecd.org/price/inflation-cpi.htm

12. World Bank Country and Lending Groups – World Bank Data Help Desk. Accessed May 13, 2021. https://datahelpdesk.worldbank.org/knowledgebase/articles/906519-world-bank-country-and-lending-groups

13. Mohammadi S-F, Rahban A, Darabeigi S, et al. Cost-effectiveness analysis of tele-retinopathy of prematurity screening in Iran. *Int J Ophthalmol*. 2021;14(4):560-566. doi:10.18240/ijo.2021.04.13

14. Moitry M, Zarca K, Granier M, et al. Effectiveness and efficiency of tele-expertise for improving access to retinopathy screening among 351 neonates in a secondary care center: An observational, controlled before-after study. *PLoS One*. 2018;13(10):e0206375.

15. Isaac M, Isaranuwatchai W, Tehrani N. Cost analysis of remote telemedicine screening for retinopathy of prematurity. *Can J Ophthalmol*. 2018;53(2):162-167.

16. Kelkar J, Agashe S, Kelkar A, Kh, ekar R. Mobile unit for retinopathy of prematurity screening and management at urban Neonatal Intensive Care Units: Outcomes and impact assessment. *Oman J Ophthalmol*. 2017;10(1):13-16.

17. Kelkar J, Kelkar A, Sharma S, Dewani J. A mobile team for screening of retinopathy of prematurity in India: Cost - effectiveness, outcomes, and impact assessment. *Taiwan J Ophthalmol*. 2017;7(3):155-159.

For peer review only - http://bmjopen.bmj.com/site/about/guidelines.xhtml

18. Rothschild MI, Russ R, Brennan KA, et al. The Economic Model of Retinopathy of Prematurity (EcROP) Screening and Treatment: Mexico and the United States. *Am J Ophthalmol*. 2016;168:110-121.

19. van den Akker-van Marle ME, van Sorge AJ, Schalij-Delfos NE. Cost and effects of risk factor guided screening strategies for retinopathy of prematurity for different treatment strategies. *Acta Ophthalmol*. 2015;93(8):706-712.

20. Wongwai P, Kingkaew P, Asawaphureekorn S, Kolatat T. A store-and-forward telemedicine for retinopathy of prematurity screen: Is it cost-effective in Thailand? *Asian Biomedicine*. 2015;9(5):665-673.

21. Black L, Hulsey T, Lee K, Parks DC, Ebeling MD. Incremental Hospital Costs Associated With Comorbidities of Prematurity. *Manag Care*. 2015;24(12):54-60.

22. Zin AA, Magluta C, Pinto MF, et al. Retinopathy of prematurity screening and treatment cost in Brazil. *Rev Panam Salud Publica*. 2014;36(1):37-43.

23. Dave HB, Gordillo L, Yang Z, Zhang MS, Hubbard GB 3rd, Olsen TW. The societal burden of blindness secondary to retinopathy of prematurity in Lima, Peru. *Am J Ophthalmol*. 2012;154(4):750-755.

24. Dunbar JA, Hsu V, Christensen M, Black B, Williams P, Beauchamp G. Cost-utility analysis of screening and laser treatment of retinopathy of prematurity. *Journal of AAPOS*. 2009;13(2):186-190.

25. Kamholz KL, Cole CH, Gray JE, Zupancic JA. Cost-effectiveness of early treatment for retinopathy of prematurity. *Pediatrics*. 2009;123(1):262-269.

26. Jackson KM, Scott KE, Graff Zivin J, et al. Cost-utility analysis of telemedicine and ophthalmoscopy for retinopathy of prematurity management. *Arch Ophthalmol*. 2008;126(4):493-499.

27. Yanovitch TL, Siatkowski RM, McCaffree M, Corff KE. Retinopathy of prematurity in infants with birth weight>or=1250 grams-incidence, severity, and screening guideline cost-analysis. *J aapos*. 2006;10(2):128-134.

28. Castillo-Riquelme MC, Lord J, Moseley MJ, Fielder AR, Haines L. Cost-effectiveness of digital photographic screening for retinopathy of prematurity in the United Kingdom. *Int J Technol Assess Health Care*. 2004;20(2):201-213.

29. Lee SK, Norm, C., et al. Evidence for changing guidelines for routine screening for retinopathy of prematurity. *Arch Pediatr Adolesc Med*. 2001;155(3):387-395.

30. Brown GC, Brown MM, Sharma S, Tasman W, Brown HC. Cost-effectiveness of treatment for threshold retinopathy of prematurity. *Pediatrics*. 1999;104(4):e47.

31. Javitt J, Cas RD, Chiang YP. Cost-effectiveness of screening and cryotherapy for threshold retinopathy of prematurity. *Pediatrics*. 1993;91(5):859-866.

32. Frick KD, Foster A. The magnitude and cost of global blindness: an increasing problem that can be alleviated. *Am J Ophthalmol*. 2003;135(4):471-476. doi:10.1016/s0002-9394(02)02110-4

33. Christ SL, Lee DJ, Lam BL, Zheng DD, Arheart KL. Assessment of the effect of visual impairment on mortality through multiple health pathways: structural equation modeling. *Invest Ophthalmol Vis Sci*. 2008;49(8):3318-3323. doi:10.1167/iovs.08-1676

34. United States, Central Intelligence Agency, United States, Central Intelligence Agency. *The World Factbook 2009 (CIA's 2008 Edition)*. Potomac Books; 2008.

35. Palmer EA, Flynn JT, Hardy RJ, et al. Incidence and early course of retinopathy of prematurity. The Cryotherapy for Retinopathy of Prematurity Cooperative Group. *Ophthalmology*. 1991;98(11):1628-1640. doi:10.1016/s0161-6420(91)32074-8

For peer review only

For peer review only - http://bmjopen.bmj.com/site/about/guidelines.xhtml

36. Good WV, Hardy RJ, Dobson V, et al. The incidence and course of retinopathy of prematurity: findings from the early treatment for retinopathy of prematurity study. *Pediatrics*. 2005;116(1):15-23. doi:10.1542/peds.2004-1413

37. Cryotherapy for Retinopathy of Prematurity Cooperative Group. Multicenter Trial of Cryotherapy for Retinopathy of Prematurity: ophthalmological outcomes at 10 years. *Arch Ophthalmol*. 2001;119(8):1110-1118. doi:10.1001/archopht.119.8.1110

38. Resnikoff S, Pascolini D, Etya'ale D, et al. Global data on visual impairment in the year 2002. *Bull World Health Organ*. 2004;82(11):844-851. doi:/S0042-96862004001100009

39. Husereau D, Drummond M, Petrou S, et al. Consolidated Health Economic Evaluation Reporting Standards (CHEERS) statement. *Value Health*. 2013;16(2):e1-5. doi:10.1016/j.jval.2013.02.010

40. Watts RD, Li IW. Use of Checklists in Reviews of Health Economic Evaluations, 2010 to 2018. *Value Health*. 2019;22(3):377-382. doi:10.1016/j.jval.2018.10.006

41. Le JT, Qureshi R, Twose C, et al. Evaluation of Systematic Reviews of Interventions for Retina and Vitreous Conditions. *JAMA Ophthalmol*. 2019;137(12):1399-1405. doi:10.1001/jamaophthalmol.2019.4016

42. Mandrik OL, Severens JLH, Bardach A, et al. Critical Appraisal of Systematic Reviews With Costs and Cost-Effectiveness Outcomes: An ISPOR Good Practices Task Force Report. *Value Health*. 2021;24(4):463-472. doi:10.1016/j.jval.2021.01.002

43. Blencowe H, Lawn JE, Vazquez T, Fielder A, Gilbert C. Preterm-associated visual impairment and estimates of retinopathy of prematurity at regional and global levels for 2010. *Pediatr Res*. 2013;74(Suppl 1):35-49. doi:10.1038/pr.2013.205

44. Zupancic JAF, Ying G-S, de Alba Campomanes A, Tomlinson LA, Binenbaum G, G-ROP Study Group. Evaluation of the economic impact of modified screening criteria for retinopathy of prematurity from the Postnatal Growth and ROP (G-ROP) study. *J Perinatol*. 2020;40(7):1100-1108. doi:10.1038/s41372-020-0605-5

45. Aly H, Othman HF, Munster C, Das A, Sears J. The US National Trend for Retinopathy of Prematurity. *Am J Perinatol*. Published online February 16, 2021. doi:10.1055/s-0041-1723830

46. Holmström G, Hellström A, Gränse L, et al. New modifications of Swedish ROP guidelines based on 10-year data from the SWEDROP register. *Br J Ophthalmol*. 2020;104(7):943-949. doi:10.1136/bjophthalmol-2019-314874

47. Kovács G, Somogyvári Z, Maka E, Nagyjánosi L. Bedside ROP screening and telemedicine interpretation integrated to a neonatal transport system: Economic aspects and return on investment analysis. *Early Hum Dev*. 2017;106-107:1-5. doi:10.1016/j.earlhumdev.2017.01.007

48. Agarwal K, Jalali S. Classification of retinopathy of prematurity: from then till now. *Community Eye Health*. 2018;31(101):S4-S7.

49. Nguyen QD, Tawansy K, Hirose T. Recent advances in retinopathy of prematurity. *Int Ophthalmol Clin*. 2001;41(4):129-151. doi:10.1097/00004397-200110000-00013

50. Lundgren P, Jacobson L, Hård A-L, et al. High rate and large intercentre variability in retreatment of retinopathy of prematurity in infants born <24 gestational weeks. *BMJ Open Ophthalmol*. 2021;6(1):e000695. doi:10.1136/bmjophth-2020-000695

51. Shah PK, Prabhu V, Karandikar SS, Ranjan R, Narendran V, Kalpana N. Retinopathy of prematurity: Past, present and future. *World J Clin Pediatr*. 2016;5(1):35-46. doi:10.5409/wjcp.v5.i1.35

For peer review only - http://bmjopen.bmj.com/site/about/guidelines.xhtml

52. Vision impairment and blindness. World Health Organization. Accessed August 26, 2021. https://www.who.int/news-room/fact-sheets/detail/blindness-and-visual-impairment

53. Naguib MM, Soares RR, Anzures R, et al. Regionally Specific Economic Impact of Screening and Treating Retinopathy of Prematurity in Middle-Income Societies in the Philippines. *J Pediatr Ophthalmol Strabismus*. 2019;56(6):388-396. doi:10.3928/01913913-20190925-02

54. Köberlein J, Beifus K, Schaffert C, Finger RP. The economic burden of visual impairment and blindness: a systematic review. *BMJ Open*. 2013;3(11):e003471. doi:10.1136/bmjopen-2013-003471

55. Chakravarthy U, Biundo E, Saka RO, Fasser C, Bourne R, Little J-A. The Economic Impact of Blindness in Europe. *Ophthalmic Epidemiol*. 2017;24(4):239-247. doi:10.1080/09286586.2017.1281426

56. Humayun J, Löfqvist C, Ley D, Hellström A, Gyllensten H. Systematic review of the healthcare cost of bronchopulmonary dysplasia. *BMJ Open*. 2021;11(8):e045729. doi:10.1136/bmjopen-2020-045729

For peer review only

For peer review only - http://bmjopen.bmj.com/site/about/guidelines.xhtml

Tables

Table 1. Overview of Studies Included in This Review.

#	First author (year)	Country (study period) Setting	Study design	ROP definition	Sample size (% of infants with ROP treated)	Inclusion criteria	Mean cost per child with ROP (value year and currency as reported in the original publication)	Cost perspective: cost inclusion
1	Mohammadi (2021) [13]	Iran (2017) Data from Farabi eye hospital	Decision Analytical Model from case series	Threshold ROP	Total: 126 ROP: 126	Randomly selected infants with treatment requiring ROP	**Treatment**: US$1107/infant	Unclear perspective: out-of-pocket charges[a]

For peer review only - http://bmjopen.bmj.com/site/about/guidelines.xhtml

2	Moitry (2018) 14	France (2012 and 2014-2015) Data from two hospitals CHSF and Port-Royal	Retrospective, before-and-after study	Type 1 ROP	Not specified	GA<33 w or BW<1500 g	**Screening**: €37/exam	Health system: direct costs
3	Isaac (2018) 15	Canada (2009–2014) Data from Ontario Ministry of Health and Long-Term Care	Retrospective cohort study (chart review)	Type 1 ROP	Total: 174 ROP: 64 Treated: 3 (5.6%)	BW<1500 g or GA<30 w	**Screening HSN**: C$346/exam (SD: C$306) **Screening RVH**: C$375/exam (SD: C$300)	Health system: direct costs (excluding equipment and maintenance)
4	Kelkar (2017a) 16	India (2009–2011) Mobile ROP screening unit	Public health intervention[b] from case series	ICROP guidelines	Total: 104 ROP: 34 Treated: 5 (15%)	BW<1700 g or GA<34 w	**Screening**: US$240/exam[c]	Health system: direct healthcare costs

For peer review only - http://bmjopen.bmj.com/site/about/guidelines.xhtml

							**Identifying an infant with ROP:** US$735/infant[c] **Treatment**: US$6500/infant	(including salaries and equipment)
5	Kelkar (2017b) [17]	India (2013–2015) Data from 5 NICUs	Public health intervention[b] from case series	ICROP guidelines	Total: 102 ROP: 32 Treated: 4 (15%)	BW<1700 g or GA<34 w	**Screening**: US$199/infant[d] **Identifying an infant with ROP**: US$596/infant[d] **Treatment**: US$4137/infant	Health system: direct costs (including salaries and equipment)
6	Rothschild (2016) [18]	Mexico and US (2014) Data from pediatric eye	Decision Analytical Model from case series	ROP caused blindness (WHO)	Total: 95	BW<1500 g	**US screening**: US$981/infant **Mexico screening:** US$333/infant	Third party payer: charges (including

For peer review only - http://bmjopen.bmj.com/site/about/guidelines.xhtml

		clinics and schools for the blind in Atlanta, Georgia, and Mexico City Blindness costs from the literature[32] and other secondary sources.					**US treatment**: US$4037/infant **Mexico treatment:** US$505/infant **US follow-up**: US$1538/infant **Mexico follow-up**: US$2214/infant **US blindness cost**: US$84586/infant **Mexico blindness cost:** US$24413/infant	labor and equipment) Societal costs: expenses for raising a blind child
7	van der Akker-van Merle	Netherlands (2009) Data from NEDROP study	Retrospective cohort study	ICROP guidelines	Total: 1380 ROP: 29 Treated: 17 (59%)	GA<32 w or BW<1500 g	**Screening**: €109/exam **Treatment**: €2755/infant	Health system: direct costs

For peer review only - http://bmjopen.bmj.com/site/about/guidelines.xhtml

	(2015) [19]	and PRN database						
8	Wongwai (2015) [20]	Thailand (2013) Hypothetical data and cohort Blindness costs using secondary data on annual government subsidies and utilities from the literature[33]	Decision Analytical Model from prospective cohort study	ET-ROP criteria	Total: 100 ROP: 9		**Screening**: THB 142/infant **Treatment**: THB (SE) 1053 (316)/infant **Lifetime cost of blindness**: THB 146,000 **Telemedicine screening**: THB 17,397/QALY (3% disc. rate)	Third party payer: charges (including labor and equipment)
9	Black (2015) [21]	US (2001–2010) Medical University of South Carolina	Retrospective cohort study	ROP stage 4	Total: 4292 ROP: 7 Treated: 7 (100%)	GA: 23–37 w	**Cost increase due to ROP if:** **GA (23 w):** US$19,513	Hospital: direct costs

For peer review only

For peer review only - http://bmjopen.bmj.com/site/about/guidelines.xhtml

							**GA (mean, 34.3 w):** US$23,121 **GA (37 w):** US$41,161	
10	Zin (2014) [22]	Brazil (2004–2006) 6 NICUs in Rio de Janeiro	Decision Analytical Model from case series and expert opinion	ICROP criteria	Total: 869 ROP: 70 Treated: 70 (100%)	BW<1500 g	**Screening**: US$18/infant **Treatment**: US$398/infant	Health system: direct costs (including labor and equipment)
11	Dave (2012) [23]	Peru (2009) Data from local hospital's NICU and from 2002 study[38]	Retrospective cohort study	ROP stage 1–5 with/without plus disease	Total: 1239 ROP: 80		**Screening and treatment**: US$2496/infant **Follow-up** (3 visits): US$54 **ROP caused blindness:** US$123,806/infant	Health system: direct costs (including equipment, facility, labor and supplies)

For peer review only - http://bmjopen.bmj.com/site/about/guidelines.xhtml

For peer review only

		Secondary source for blindness costs[34]						Societal costs: expenses for blindness
12	Dunbar (2009)[24]	US (2004–2006) Medicare and Medicaid reimbursement data from California and Louisiana	Microsimulation model from retrospective cohort study	Type 1 ROP	Total: 515<br>ROP: 58<br>Treated: 58 (100%)	BW<1500 g or GA<28 w	**Screening**: US$93/exam<br>**Screening**: US$316/infant<br>**Treatment w/o anesthesia**: US$1371/infant<br>**Screening and treatment:** US$1565/QALY (3% disc. rate)	Third-party payer (Medicare and Medicaid): charges (excluding anesthesia)

For peer review only - http://bmjopen.bmj.com/site/about/guidelines.xhtml

13	Kamholz (2009) [25]	US (2005) Data from ET-ROP study	Decision Analytical Model from randomized trial and expert opinion	Type 1 ROP	ROP: 357	BW<1250 g or GA<32 w	**Screening**: US$189/exam (US$56–$251); **treatment w/o anesthesia**: US$2423 (US$638–$3223) **Anesthesia**: US$1849 (US$925–$3698)	Third-party payer: charges
14	Jackson (2008) [26]	US (2006) Data from CRYO-ROP and ET-ROP studies	Decision Analytical Model from randomized trial	Type 1 ROP	Refer to published data on 4099 infants (65.8% with ROP[35]) and 6998 infants (68% with ROP[36])	BW<1251g	**Screening**: US$160/exam **Screening and treatment**: US$4410/QALY (3% disc. rate.)	Third-party payer (Medicare): charges

For peer review only - http://bmjopen.bmj.com/site/about/guidelines.xhtml

15	Yanowitch (2006) [27]	US (2001–2004) Data from Dean A. McGee Eye Institute and OUHSC campus	Retrospective cohort study (chart review)	CRYO-ROP and ET-ROP criteria	Total: 259<br>ROP: 11<br>Treated: 1 (9%)	BW 1250–1800 g	**Screening**: US$230/infant<br>**Treatment**: US$2000/infant	Third-party payer: charges
16	Castillo-Riquelme (2004) [28]	UK (1997-1998) Data from published data[37] and local NICU	Decision Analytical Model from case series and expert opinion	ROP stage 3	ROP: 235	GA<32 or BW<1501 g	**Screening**: £49/exam<br>**Screening:** £279/infant<br>**Treatment**: £540/one eye<br>**Treatment:** £702/two eyes<br>**Follow-up** (10 years): £786/infant	Health system: direct costs (including equipment and maintenance)
17	Lee (2001) [29]	Canada (1996-1997)	Retrospective cohort study	Threshold ROP	Total: 16,424	Different criteria at	**Screening**: C$236/infant<br>**Treatment**: C$2655/infant	Health system: direct costs

For peer review only - http://bmjopen.bmj.com/site/about/guidelines.xhtml

		Data from 14 NICU				different NICU		
18	Brown (1999) [30]	US (1998) Database from Pennsylvania	Microsimulation model from randomized trial	Threshold ROP	ROP: 291 Treated: 291 (100%) but only one treated eye per infant	BW<1251 g	**Treatment**: US$1452/infant **Treatment consultation**: US$140/exam **Treatment**: US$678/QALY (3% disc. rate)	Third-party payer: charges
19	Javitt (1993) [31]	US (1989) Medicare reimbursement data	Microsimulation model from retrospective cohort study	Threshold ROP or PNA 24 weeks from CRYO-ROP	Total: 18,220 ROP: 1000 Treated: 1000 (100%)	BW: 500–1249 g	**Screening (1st visit)**: US$84/exam **Screening (subsequent visit)**: US$68/exam **Screening (weekly)**: US$6045/QALY	Third-party payer: charges (excluding equipment and personnel training cost)

For peer review only - http://bmjopen.bmj.com/site/about/guidelines.xhtml

							**Screening (biweekly)**: US$3623/QALY **Screening (monthly)**: US$2488/QALY	

[a] Assumption based on methods description indicating cost data collected through survey to parents.

[b] Studies of the introduction of new screening programs.

[c] Screening costs and costs for identifying an infant with ROP are reduced by 22.6% to account for transport costs.

[d] Screening costs and costs for identifying an infant with ROP are reduced by 0.245% to account for transport costs.

Abbreviations: BW=birth weight; disc.=discount; GA=gestational age; HSN=Health Sciences North in Sudbury, Canada; NICU=neonatal intensive care unit; PNA=postnatal age; QALY=quality-adjusted life years; ROP=retinopathy of prematurity; RVH=Royal Victoria Hospital in Barrie, Canada; US=United States of America; WHO=World Health Organization

For peer review only

For peer review only - http://bmjopen.bmj.com/site/about/guidelines.xhtml

Table 2. Costs for Screening for ROP Among Preterm Infants (in 2020 values)

#	First author (year)	Screening costs		Treatment costs	Evidence rating	Cost inclusion
		**Mean per exam** (US$)	**Mean per infant** (US$)	**Mean per infant** (US$)		
1	Mohammadi (2021) [13]	-	**-**	1169	4	Charges
2	Moitry (2018) [14]	44	**-**	-	3	Direct cost
3	Isaac (2018) [15]	HSN: 342<br>RVH: 371	**-**	-	3	Direct cost **not including equipment**
4	Kelkar (2017a) [16]	253	**-**	6500	4	Direct cost including equipment and labor
5	Kelkar (2017b) [17]	210	**-**	4137	4	Direct cost including equipment and labor
6	Rothschild (2016) [18]		**US: 1072**<br>**Mexico: 362**	US: 4413<br>Mexico: 552	4	Direct cost including equipment and labor

For peer review only - http://bmjopen.bmj.com/site/about/guidelines.xhtml

7	van der Akker-van Merle (2015) [19]	160	-	4064[a]	3	Direct cost
8	Wongwai (2015) [20]	5	-	38	2	Charges including equipment and labor
9	Black (2015) [21]	-	-	-	3	-
10	Zin (2014) [22]	20	-	450	5	Direct cost including equipment and labor
11	Dave (2012) [23]	-	-	-	3	-
12	Dunbar (2009) [24]	119	405	1759	3	Charges
13	Kamholz (2009) [25]	250	-	5661[a]	5	Charges
14	Jackson (2008) [26]	205	-	-	1	Charges
15	Yanowitch (2006) [27]	-	324	2814	3	Charges
16	Castillo-Riquelme (2004) [28]	106	602	Unilateral: 1165	5	Direct cost including

For peer review only - http://bmjopen.bmj.com/site/about/guidelines.xhtml

				Bilateral: 1514		equipment and maintenance
17	Lee (2001) [29]	Unilateral: 112	-	2507	3	Direct cost
18	Brown (1999) [30]	-	-	2527	1	Charges
19	Javitt (1993) [31]	First: 183 Follow-up: 149	-	-	3	Charges

Evidence rating indicates the quality of evidence rating of included studies: 1=e.g., properly powered randomized controlled trials; 2=e.g., prospective cohort studies; 3=e.g., retrospective cohort studies; 4=case series with or without intervention or cross-sectional study; 5=e.g., opinion of respected authorities.

[a] Unit cost per treatment.

Abbreviations: HSN=Health Sciences North in Sudbury, Canada; ROP=retinopathy of prematurity; RVH=Royal Victoria Hospital in Barrie, Canada; US=United States of America

For peer review only - http://bmjopen.bmj.com/site/about/guidelines.xhtml

Figure Titles and Legends

Figure 1. Map of data availability and costs per a) screening visit and b) treatment. The map illustrates reported costs or means of reported costs per country for included studies in US$. In studies presenting only total screening cost per infant or by first/follow-up visits,[18,27,31] the cost level per screening was calculated under the assumption of four screening visits per infant. Where only screening cost per eye was reported,[29] it was duplicated to obtain the cost level per screening. In studies reporting only unit cost per treatment,[19,25] the unit cost was assumed to indicate the cost level of treatment per infant. Where costs were reported separately for unilateral and bilateral treatment,[28] a weighted mean cost was calculated assuming that 75% of treatments were bilateral.

Figure 2. Forest plot of treatment costs, by country categorization. In parentheses, ER of included studies: 1=e.g., properly powered randomized controlled trials; 2=e.g., prospective cohort studies; 3=e.g., retrospective cohort studies; 4=case series with or without intervention or cross-sectional study; 5=e.g., opinion of respected authorities. Abbreviations: CI=confidence interval; ER=Evidence rating; REML=Restricted Maximum Likelihood.

Figure 3. Forest plot of treatment costs, cumulative results by year, and country categorization. In parentheses, ER of included studies: 1=e.g., properly powered randomized controlled trials; 2=e.g., prospective cohort studies; 3=e.g., retrospective cohort studies; 4=case series with or without intervention or cross-sectional study; 5=e.g., opinion of respected authorities. Abbreviations: CI=confidence interval; ER=Evidence rating; REML=Restricted Maximum Likelihood.

For peer review only

Figure 1a) World map - cost per screening

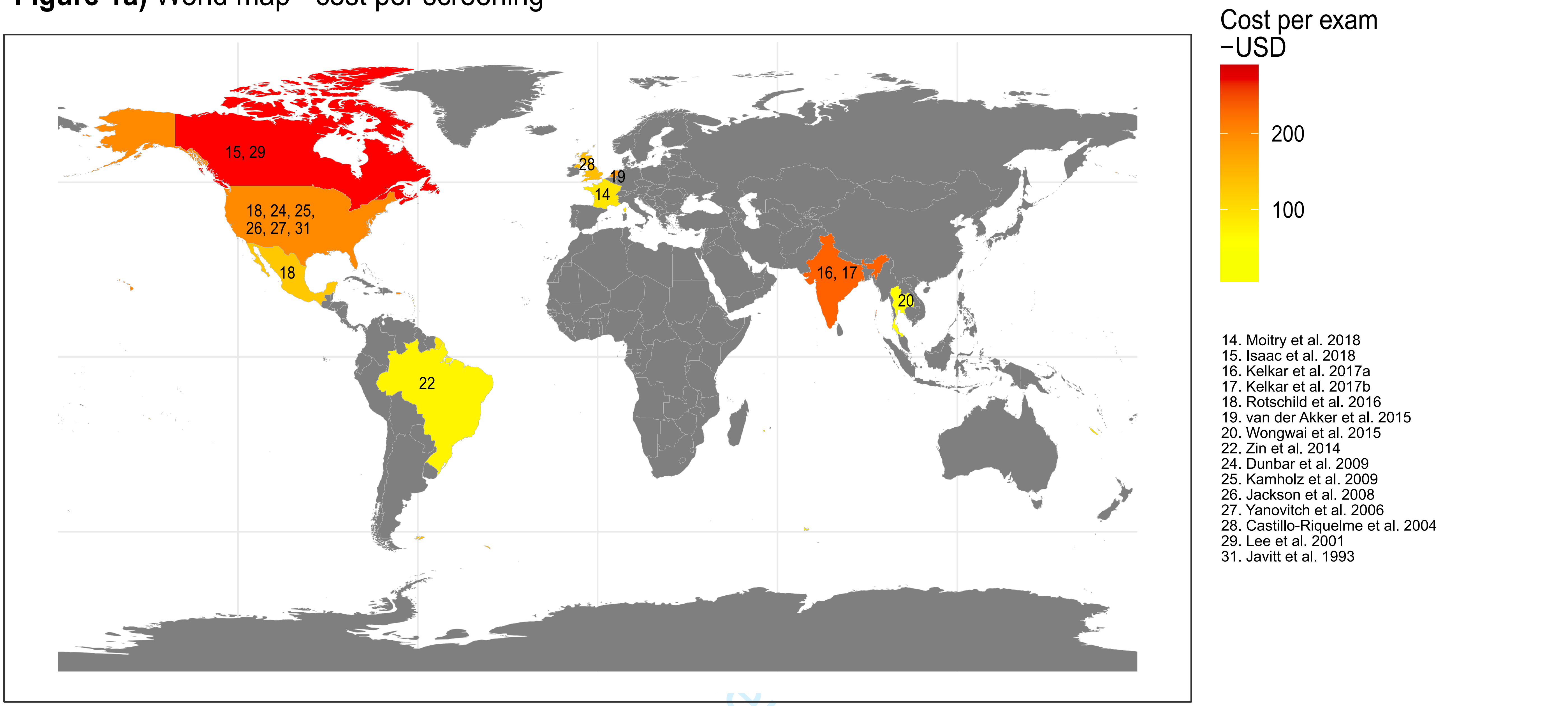

1b) World map −cost per treatment

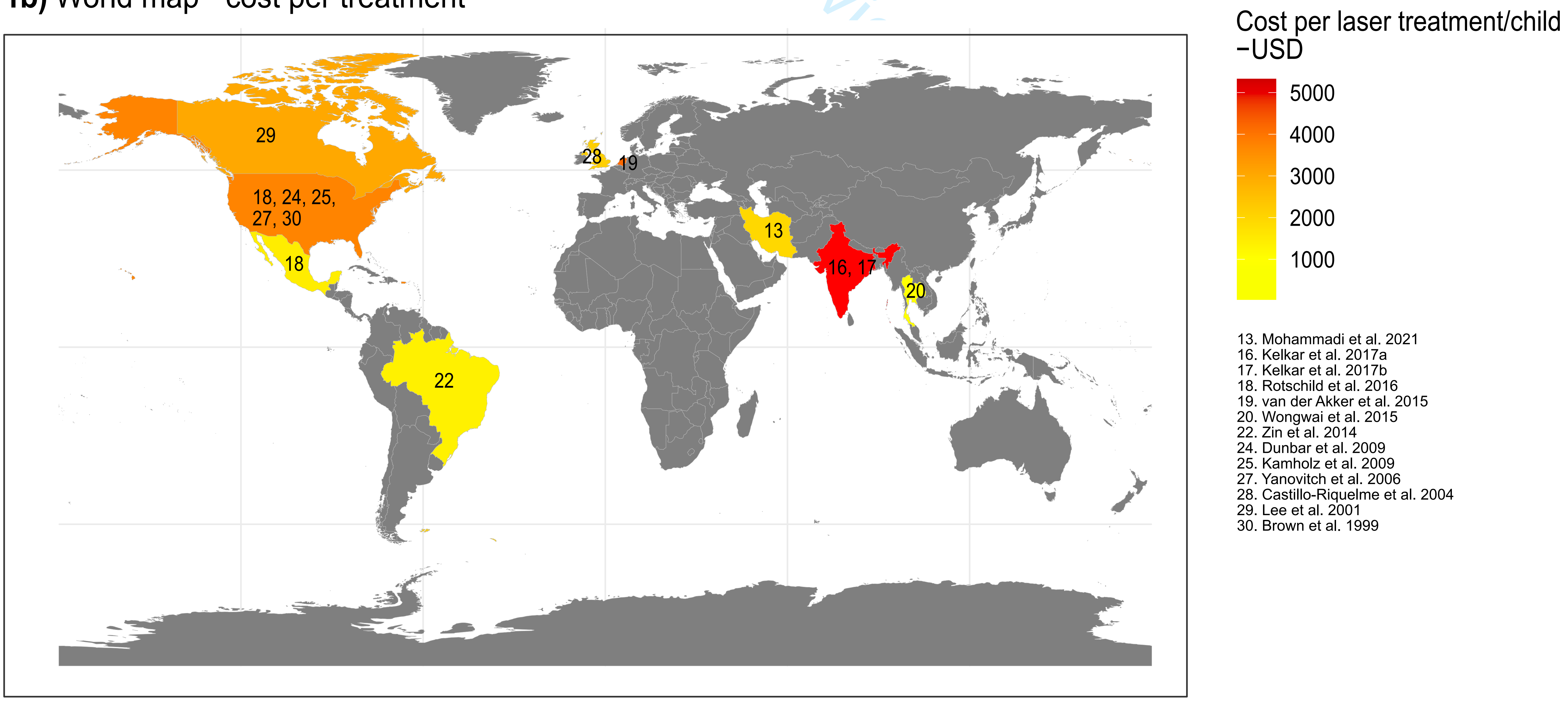

Study	Mean cost (95% CI)
**High-income economies**	
Rothschild et al,[18] US (2016) (ER:4)	4413.00 (2493.65 - 6332.35)
van der Akker-van Merle et al,[19] (2015) (ER:3)	4064.00 (2296.44 - 5831.56)
Dunbar et al,[24] (2009) (ER:3)	1759.00 (993.96 - 2524.04)
Kamholz et al,[25] (2009) (ER:5)	5661.00 (3948.53 - 7373.47)
Yanowitch et al,[27] (2006) (ER:3)	2814.00 (1590.11 - 4037.89)
Castillo-Riquelme et al,[28] (2004) (ER:5)	1426.75 (806.21 - 2047.29)
Lee et al,[29] (2001) (ER:3)	2507.00 (1416.63 - 3597.37)
Brown et al,[30] (1999) (ER:1)	2527.00 (1427.93 - 3626.07)
Heterogeneity: τ^2 = 1.48e+06, I^2 = 82.99%, H^2 = 5.88	2960.35 (2003.36 - 3917.34)
Test of $\theta_i = \theta_j$: Q(7) = 33.77, p = 0.00	
**Lower-middle-income economies**	
Mohammadi et al,[13] (2021) (ER:4)	1169.00 (660.57 - 1677.43)
Kelkar et al,[16] (2017a) (ER:4)	6500.00 (3672.95 - 9327.05)
Kelkar et al,[17] (2017b) (ER:4)	4137.00 (2337.69 - 5936.31)
Heterogeneity: τ^2 = 6.23e+06, I^2 = 90.53%, H^2 = 10.56	3692.43 (669.58 - 6715.28)
Test of $\theta_i = \theta_j$: Q(2) = 21.87, p = 0.00	
**Upper-middle-income economies**	
Rothschild et al,[18] Mexico (2016) (ER:4)	552.00 (311.92 - 792.08)
Wongwai et al,[20] (2015) (ER:2)	38.00 (16.44 - 59.56)
Zin et al,[22] (2014) (ER:5)	450.00 (254.28 - 645.72)
Heterogeneity: τ^2 = 72208.92, I^2 = 92.23%, H^2 = 12.87	329.16 (8.93 - 649.39)
Test of $\theta_i = \theta_j$: Q(2) = 33.95, p = 0.00	
**Overall**	
Heterogeneity: τ^2 = 3.02e+06, I^2 = 98.98%, H^2 = 98.06	2473.47 (1502.80 - 3444.15)
Test of $\theta_i = \theta_j$: Q(13) = 269.57, p = 0.00	
Test of group differences: Q_b(2) = 30.11, p = 0.00	

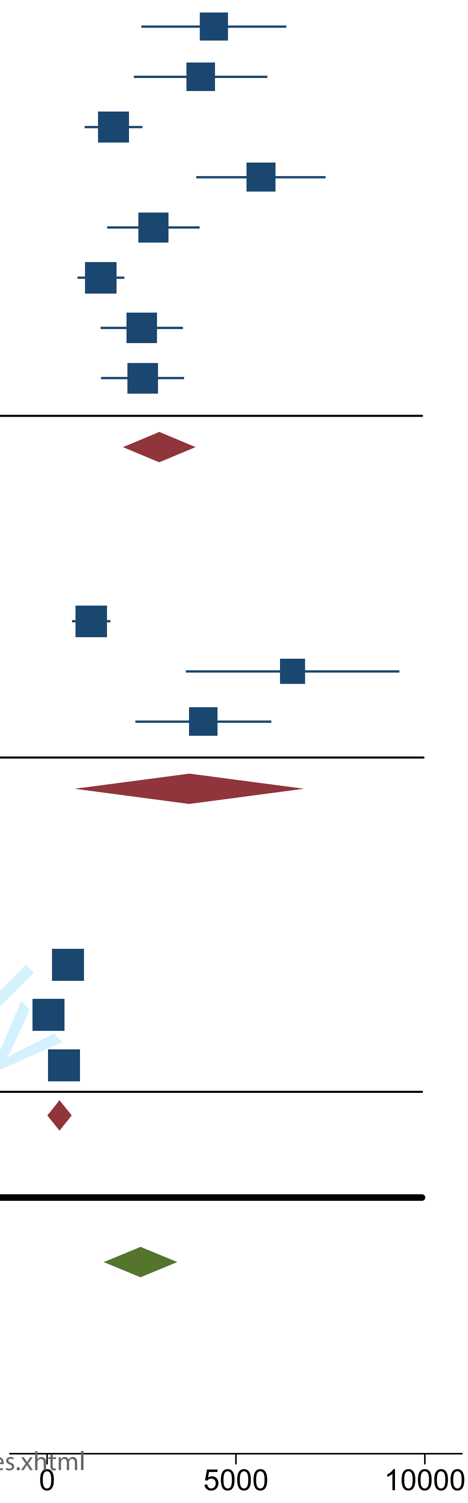

Random-effects REML model

For peer review only

Study	Mean cost (95% CI)
**High-income economies**	
Brown et al,[30] (1999) (ER:1)	2527.00 (1427.93 - 3626.07)
Lee et al,[21] (2001) (ER:3)	2516.92 (1742.86 - 3290.99)
Castillo-Riquelme et al,[28] (2004) (ER:5)	2039.56 (1240.70 - 2838.41)
Yanowitch et al,[27] (2006) (ER:3)	2193.88 (1478.16 - 2909.59)
Dunbar et al,[24] (ER:3)	2056.83 (1508.58 - 2605.09)
Kamholz et al,[25] (2009) (ER:5)	2634.39 (1579.11 - 3689.66)
van der Akker-van Merle et al,[19] (2015) (ER:3)	2798.19 (1805.71 - 3790.68)
Rothschild et al,[18] US (2016) (ER:4)	2960.35 (2003.36 - 3917.34)
**Lower-middle-income economies**	
Kelkar et al,[16] (2017a) (ER:4)	6500.00 (3672.95 - 9327.05)
Kelkar et al,[17] (2017b) (ER:4)	5056.60 (2798.51 - 7314.69)
Mohammadi et al,[13] (2021) (ER:4)	3692.43 (669.58 - 6715.28)
**Upper-middle-income economies**	
Zin et al,[22] (2014) (ER:5)	450.00 (254.28 - 645.72)
Wongwai et al,[20] (2015) (ER:2)	232.05 (-171.03 - 635.12)
Rothschild et al,[18] Mexico (2016) (ER:4)	329.16 (8.93 - 649.39)

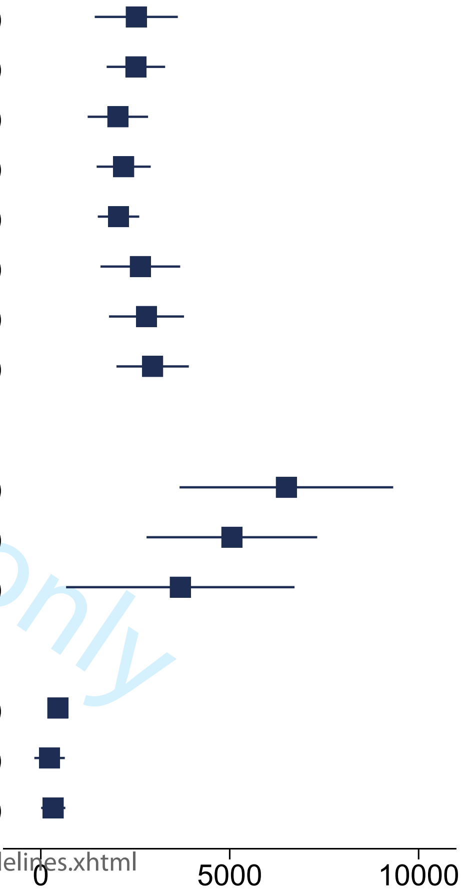

Random-effects REML model

For peer review only
For peer review only - http://bmjopen.bmj.com/site/about/guidelines.xhtml

Online-Only Supplements

Costs associated with Retinopathy of prematurity: A Systematic Review and Meta-analysis

Authors

Hanna Gyllensten[1,2], Associate professor

Jhangir Humayun[1,2], B.Sc.

Ulrika Sjöbom[1,3], M.Sc.

Ann Hellström[3], Professor

Chatarina Löfqvist[1,2,3], Associate professor

Author affiliations

[1] Institute of Health and Care Sciences, Sahlgrenska Academy, University of Gothenburg, Box 457, SE-405 30 Gothenburg, Sweden.

[2] Centre for Person-Centred Care (GPCC), University of Gothenburg, Box 457, SE-405 30 Gothenburg, Sweden.

[3] Department of Clinical Neuroscience, Institute of Neuroscience and Physiology, Sahlgrenska Academy, University of Gothenburg, Gothenburg, Sweden.

Content

Authors 1

Author affiliations 1

eTable 1. Search terms 2

eTable 2. Data extraction sheet 2

Data extraction 2

Quality assessment (according to instrument developed by Evers et al[1]) 2

eTable 3. Checklist for the quality appraisal of included papers (from Evers et al[1]) 2

eFigure 1. Flow diagram shows the study selection process, following the PRISMA guidelines.[21] 4

eTable 4. Excluded articles* 5

eFigure 2. Cost model 6

Preterm birth 7

ROP screening 7

Lifetime (treatment and follow-up) 7

References 8

For peer review only

eTable 1. Search terms

Database	Search string
Pubmed	(((((((Retinopathy) AND Prematur*) OR ((Terry) AND Syndrom*) OR ("ROP"[Title/Abstract]) OR "Retinopathy of Prematurity"[Mesh])) AND (("Economics"[Mesh]) OR ((economic*[Title/Abstract] OR cost[Title/Abstract] OR costs[Title/Abstract] OR costly[Title/Abstract] OR costing[Title/Abstract] OR price[Title/Abstract] OR prices[Title/Abstract] OR pricing[Title/Abstract] OR pharmacoeconomic*[Title/Abstract])))))
Scopus	(TITLE-ABS-KEY ("Retinopath*") AND TITLE-ABS-KEY ("Prematur*")) OR (TITLE-ABS-KEY ("Retrolental") AND TITLE-ABS-KEY ("Fibroplas*")) OR (TITLE-ABS-KEY ("Terry") AND TITLE-ABS-KEY ("Syndrom*")) AND (TITLE-ABS-KEY (economic* OR cost OR cos OR costly OR costing OR price OR prices OR pricing OR pharmacoeconomic*))

eTable 2. Data extraction sheet

Data extraction

- Reviewer
- Reference (APA)
- Aim/Objective
- Study design
- When was it conducted
- Setting including country and hospital name/database
- How is ROP severity defined
- Total study participants
- Patients with ROP (N)
- Patient group description
- Controls (N)
- Control group description
- Average cost of screening (total per infant/per visit/per eye)
- What costs are measured
- How are the costs measured
- Average Cost for infants with diagnosed sight-threatening ROP
- What costs are measured
- How are the costs measured
- Costs from which year (if adjusted, which year)
- Perspective: cost analysis
- Time horizon of cost analysis
- Funding
- Limitations: Confounders and biases reported
- Conclusions (by author)

Quality assessment (according to instrument developed by Evers et al[1])

1. Is the study population clearly described?
2. Are competing alternatives clearly described?
3. Is a well-defined research question posed in answerable form?
4. Is the economic study design appropriate to the stated objective?
5. Is the chosen time horizon appropriate in order to include relevant costs and consequences?
6. Is the actual perspective chosen appropriate?
7. Are all important and relevant costs for each alternative identified?
8. Are all costs measured appropriately in physical units?
9. Are costs valued appropriately?
10. Are all important and relevant outcomes for each alternative identified?
11. Are all outcomes measured appropriately?
12. Are outcomes valued appropriately?
13. Is an incremental analysis of costs and outcomes of alternatives performed?
14. Are all future costs and outcomes discounted appropriately?
15. Are all important variables, whose values are uncertain, appropriately subjected to sensitivity analysis?
16. Do the conclusions follow from the data reported?
17. Does the study discuss the generalizability of the results to other settings and patient/client groups?
18. Does the article indicate that there is no potential conflict of interest of study researcher(s) and funder(s)?
19. Are ethical and distributional issues discussed appropriately?

For peer review only

eTable 3. Checklist for the quality appraisal of included papers (from Evers et al[1])

First authors / Checklist items[a]	Black[2]	Brown[3]	Castillo-Requilme[4]; Javitt[5]; Lee[6]; Rothchild[7]; Wongwai[8]	Dave[9]	Dunbar[10]	Isaac[11]	Kamholz[12]; Jackson[13]	Kelkar (2017a)[14]; Kelkar (2017b)[15]	Mohammadi[16]	Moitry[17]	Van den Akker-van Merle[18]	Yanowitch[19]	Zin[20]	**Total**
1	+	+	+	+	+	+	-	+	-	+	+	+	+	**16**
2	+	+	+	+	+	+	+	+	+	+	+	+	+	**19**
3	+	+	+	+	+	+	+	+	+	+	+	+	+	**19**
4	+	+	+	+	+	+	+	+	+	+	+	+	+	**19**
5	+	+	+	+	+	+	+	+	+	+	+	+	+	**19**
6	+	+	+	+	+	+	+	+	-	+	+	+	+	**18**
7	+	+	+	+	+	+	+	+	+	+	+	+	+	**19**
8	+	+	+	+	+	+	+	+	+	+	+	+	+	**19**
9	+	+	+	+	+	+	+	+	+	+	+	+	+	**19**
10	+	+	+	+	+	+	+	+	+	+	+	+	+	**18**
11	+	+	+	+	+	+	+	+	+	+	+	+	+	**19**
12	+	+	+	+	+	+	+	+	+	+	+	+	+	**19**
13	+	-	+	+	+	+	+	-	-	-	+	+	+	**14**
14	-	-	+	-	+	-	+	-	-	+	+	+	-	**11**
15	+	-	+	-	-	-	+	-	-	+	-	-	+	**10**
16	+	+	+	+	+	+	+	+	+	+	+	+	+	**19**
17	+	+	+	+	+	+	+	+	+	+	+	+	+	**19**
18	+	+	+	+	-	+	+	-	+	+	-	+	+	**15**

For peer review only

19	+	+	+	+	+	+	+	-	+	+	+	+	+	**17**
**Total**	**18**	**16**	**19**	**17**	**17**	**17**	**18**	**14**	**14**	**18**	**17**	**17**	**18**	

[a] Item numbering according to eTable 2.

For peer review only

eFigure 1. Flow diagram shows the study selection process, following the PRISMA guidelines.[21]

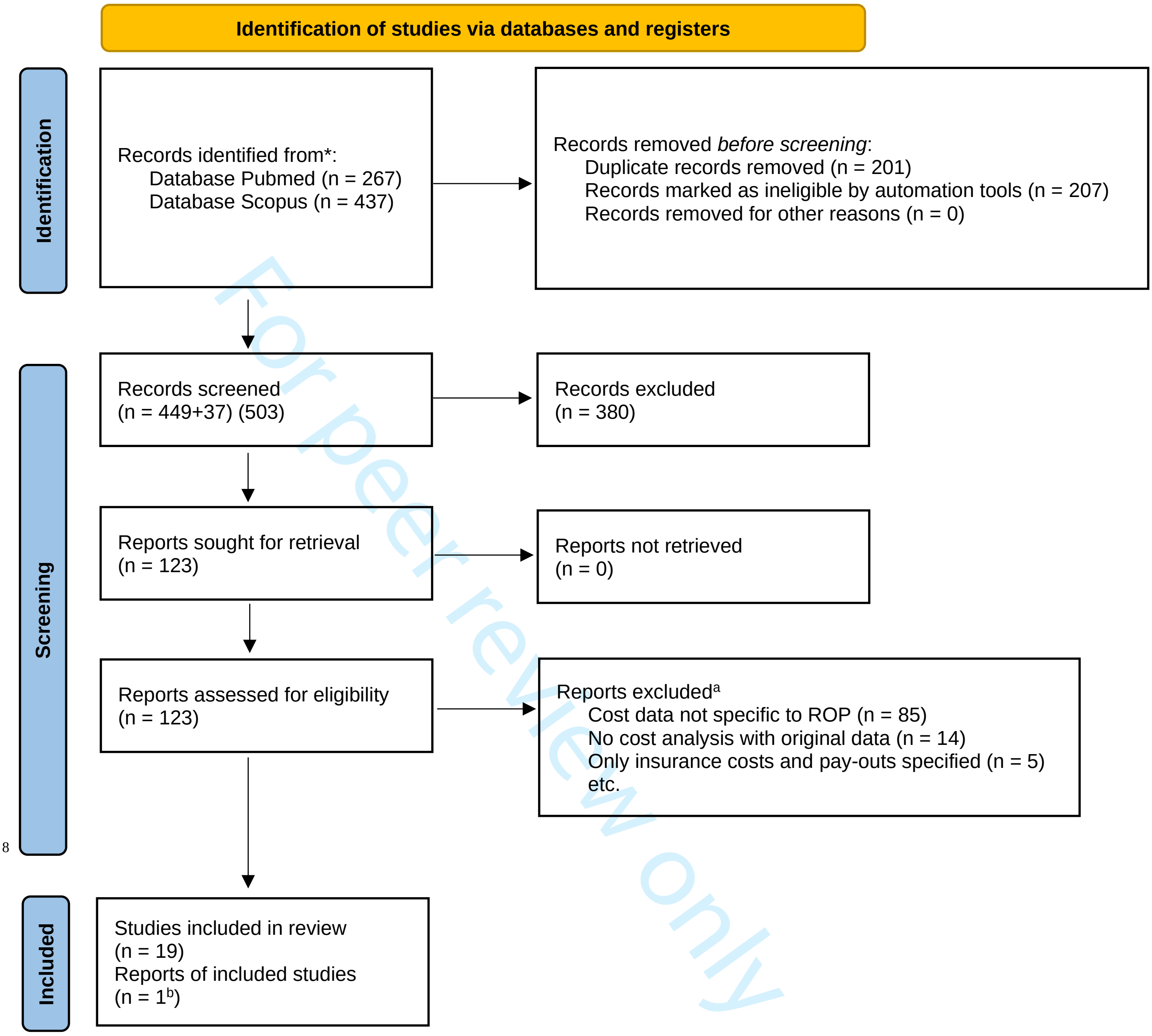

[a] For detailed reasons for exclusion of studies that might appear to meet the inclusion criteria, but which were excluded, see also eTable 4.

[b] One author[8] was contacted and clarified the currency of reported results. Another author[16] was unsuccessfully contacted to clarify cost perspective.

Abbreviations: ROP = Retinopathy of prematurity.

For peer review only

eTable 4. Excluded articles[a]

Study	Reason for exclusion
Cross 1973. Cost of preventing retrolental fibroplasia?[22]	No original cost data.
Boncz et al., 2013. [Health-economic analysis of diseases related to disturbed neonatal adaptation: A cost of illness study].[23]	Only insurance payouts.
Yo et al., 2018. Retinopathy of prematurity: the high cost of screening regional and remote infants.[24]	Transport costs but no screening costs.
Scholz and Greiner, 2019. An exclusive human milk diet for very low birth weight newborns-A cost-effectiveness and EVPI study for Germany.[25]	No ROP specific costs.
Zupancic et al., 2020. Evaluation of the economic impact of modified screening criteria for retinopathy of prematurity from the Postnatal Growth and ROP (G-ROP) study.[26]	No original cost data.

[a] In this table are listed studies that might appear to meet the inclusion criteria, but which were excluded, and why they were excluded.

Abbreviations: ROP = Retinopathy of prematurity.

For peer review only

eFigure 2. Cost model

This figure presents our preliminary suggestions for a conceptual model for costs associated with retinopathy of prematurity (ROP), with some additional comments we believe are relevant. Abbreviations: GA=gestational age; ROP=retinopathy of prematurity; VEGF=vascular endothelial growth factor.

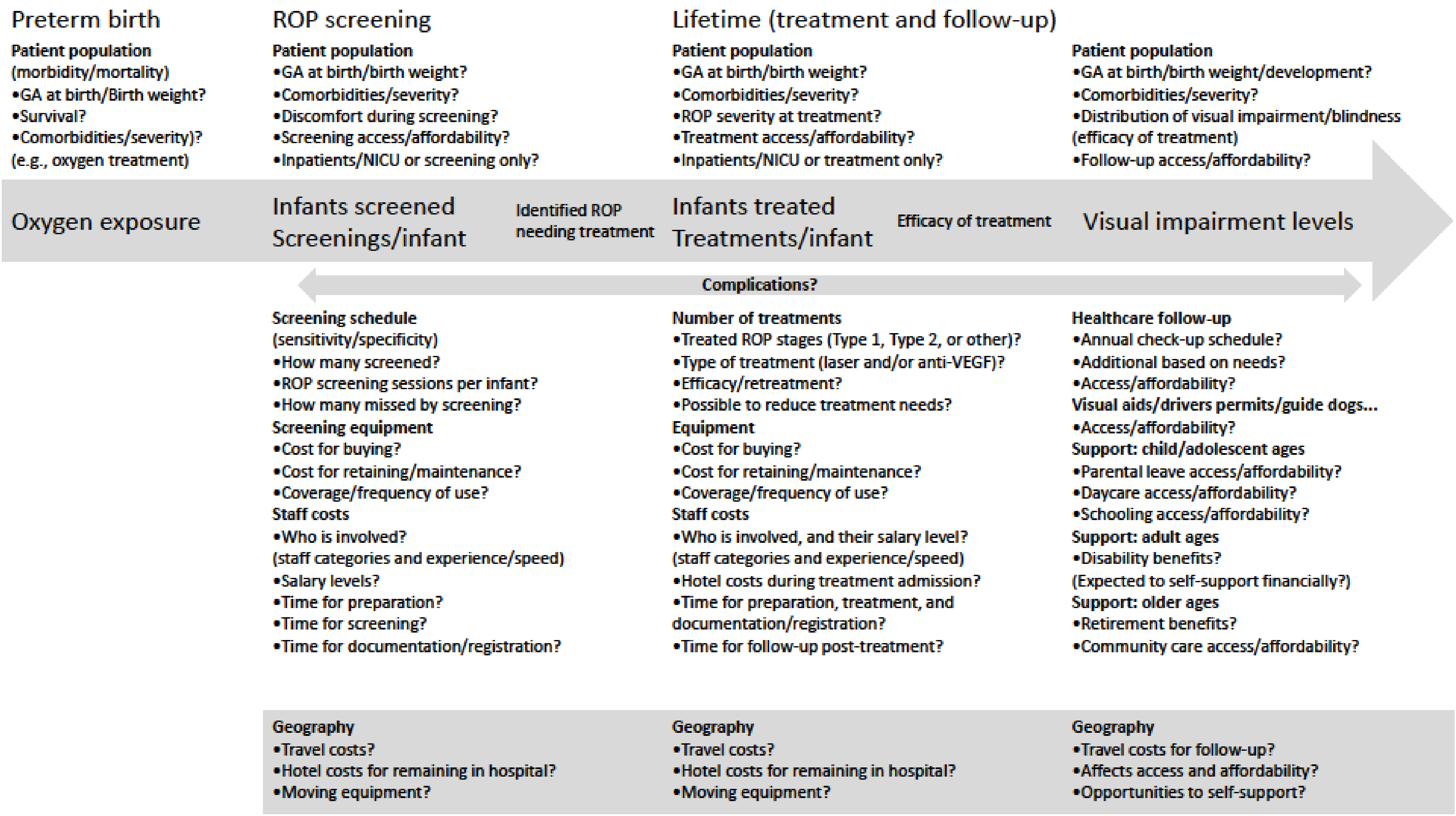

Preterm birth

It should be noted that these costs are part of a larger picture of understanding the economic impact of prematurity, which is essential knowledge in predicting the costs and consequences of introducing new interventions that affect gestational age at birth or morbidity and mortality among preterm infants. Thus, the model here is only one part and should be complemented by factors related to, e.g., bronchopulmonary dysplasia and other lung diseases, as well as other neuropsychiatric conditions. The listed items add to the previously published compartmental model of the global burden of ROP,[27] which also accounts for e.g., availability and coverage of screening programs.

ROP screening

Some evidence suggests that screening can be reduced even as infants are still identified with high sensitivity and specificity.[5] Reduced screening can be achieved through either changing the frequency of screening or limiting who is actually screened. Based on register findings in Sweden, infants born after gestational week 30 are no longer routinely screened for ROP.[28] Similarly, a study from the Netherlands found no severe ROP among infants born ≥30 gestational weeks.[29] This pattern differs from the situation in many other parts of the world. However, infants born at lower gestational age are more likely to develop ROP and severe ROP.[30]

Costs for screening in the studies included staff salaries/time, equipment and maintenance, supplies, and staff training. Although the identified studies do not detail the cost components and their associated costs, it can be expected that the reported costs of screening are to some extent underestimated. In time-and-motion studies conducted in our local hospital during a process of developing services (unpublished results), the times spent for preparatory work and documentation of screening results were 7–15 minutes and 7–12 minutes, respectively. This range included the time needed to identify infants who should be screened from those born at the facility, but excluded the time used for the actual screening. The figures can be compared to numbers provided in, e.g., Wongwai et al.,[8] citing 10 minutes used for screening by the ophthalmologist and 60 minutes for the nurse. According to Jackson et al.,[13] an average five examinations were necessary for determining if one infant would require treatment for ROP, which is in line with experiences in our hospital.

Regardless of the setting, there will also be transportation costs associated with screening. In this review, we excluded transportation costs, which are highly specific to each setting. For example, an Australian study reported flights for ROP screening to average 36–75 minutes depending on the healthcare center.[24] Transportation can thus include the time and expenses to the families coming into the hospital (or to visit a telemedicine center), or moving within the hospital if the infant remains hospitalized, but they can also reflect the cost of a specialized physician and assistant nurse or other staff category moving within or between hospitals to conduct screening. In addition to being an important cost component to consider in evaluations, the transportation aspect and hotel costs for staying in the hospital can directly affect screening. Our group has clinical experience of parents selecting not to attend planned screening visits after leaving the hospital, so that travel costs also become an issue related to increasing screening adherence and motivating attendance.

Lifetime (treatment and follow-up)

Treatment costs in individual studies included, e.g., staff salaries/time, equipment and maintenance, supplies, and staff training. Few studies reported detailed data on cost components, but Wongwai et al.,[8] for example, reported post-screening resource use of 60 minutes for an expert ophthalmologist, which we interpret to be the cost for treatment. Although case-mix and survival of extremely preterm infants were not detailed in the included studies, it can be expected that these factors will affect how many infants need treatment for ROP. For example, among infants born ≤30 gestational weeks in Sweden, 32% had any stage ROP and 6% were treated for ROP,[28] but among infants born at <24 gestational weeks, the corresponding figures were 92% and 43%.[31] Moreover, the available treatment options would affect costs, with studies suggesting, e.g., more retreatments with the more recent anti-vascular endothelial growth factor (VEGF) therapy.[28] Surgical intervention, or vitrectomy, could also apply to more severe cases,[32] in particular in countries with low access to screening. Although the costs of vitrectomy itself appear to be low,[33] there are likely other costs associated with these severe ROP cases, such as those linked to follow-up and complications.[34]

The argument regarding transportation costs is highly relevant for the treatment of ROP. The clinical reality of many countries is that patients

For peer review only

must be flown to the treatment site, or undergo multiple relocations by ambulance between local hospitals and specialized units providing the treatment.

At least in countries with high access to healthcare, it can be expected that children with ROP, and particularly those with severe forms requiring treatment, will have multiple follow-ups during childhood, adolescence, and possibly into adulthood. The low number of healthcare visits for follow-up indicated in the included articles differs considerably from the national guidelines in Sweden, recommending annual follow-up of ROP until adulthood and, after that, according to need. In a recent publication reporting on a model for predicting visual outcomes after ROP treatment,[35] follow-up every 6 months was even indicated for some patient groups.

Although costs for blindness can be expected to be similar regardless of the cause of blindness, data are available on approximate cost levels for different levels of visual impairment.[36] Thus, tapping into models for measuring costs of visual impairment can add to understanding of the long-term consequences of ROP.

For peer review only

References

1. Evers S, Goossens M, de Vet H, van Tulder M, Ament A. Criteria list for assessment of methodological quality of economic evaluations: Consensus on Health Economic Criteria. *Int J Technol Assess Health Care*. 2005;21(2):240-245.

2. Black L, Hulsey T, Lee K, Parks DC, Ebeling MD. Incremental Hospital Costs Associated With Comorbidities of Prematurity. *Manag Care*. 2015;24(12):54-60.

3. Brown GC, Brown MM, Sharma S, Tasman W, Brown HC. Cost-effectiveness of treatment for threshold retinopathy of prematurity. *Pediatrics*. 1999;104(4):e47.

4. Castillo-Riquelme MC, Lord J, Moseley MJ, Fielder AR, Haines L. Cost-effectiveness of digital photographic screening for retinopathy of prematurity in the United Kingdom. *Int J Technol Assess Health Care*. 2004;20(2):201-213.

5. Javitt J, Cas RD, Chiang YP. Cost-effectiveness of screening and cryotherapy for threshold retinopathy of prematurity. *Pediatrics*. 1993;91(5):859-866.

6. Lee SK, Norm, C., et al. Evidence for changing guidelines for routine screening for retinopathy of prematurity. *Arch Pediatr Adolesc Med*. 2001;155(3):387-395.

7. Rothschild MI, Russ R, Brennan KA, et al. The Economic Model of Retinopathy of Prematurity (EcROP) Screening and Treatment: Mexico and the United States. *Am J Ophthalmol*. 2016;168:110-121.

8. Wongwai P, Kingkaew P, Asawaphureekorn S, Kolatat T. A store-and-forward telemedicine for retinopathy of prematurity screen: Is it cost-effective in Thailand? *Asian Biomedicine*. 2015;9(5):665-673.

9. Dave HB, Gordillo L, Yang Z, Zhang MS, Hubbard GB 3rd, Olsen TW. The societal burden of blindness secondary to retinopathy of prematurity in Lima, Peru. *Am J Ophthalmol*. 2012;154(4):750-755.

10. Dunbar JA, Hsu V, Christensen M, Black B, Williams P, Beauchamp G. Cost-utility analysis of screening and laser treatment of retinopathy of prematurity. *Journal of AAPOS*. 2009;13(2):186-190.

11. Isaac M, Isaranuwatchai W, Tehrani N. Cost analysis of remote telemedicine screening for retinopathy of prematurity. *Can J Ophthalmol*. 2018;53(2):162-167.

12. Kamholz KL, Cole CH, Gray JE, Zupancic JA. Cost-effectiveness of early treatment for retinopathy of prematurity. *Pediatrics*. 2009;123(1):262-269.

13. Jackson KM, Scott KE, Graff Zivin J, et al. Cost-utility analysis of telemedicine and ophthalmoscopy for retinopathy of prematurity management. *Arch Ophthalmol*. 2008;126(4):493-499.

14. Kelkar J, Agashe S, Kelkar A, Kh, ekar R. Mobile unit for retinopathy of prematurity screening and management at urban Neonatal Intensive Care Units: Outcomes and impact assessment. *Oman J Ophthalmol*. 2017;10(1):13-16.

15. Kelkar J, Kelkar A, Sharma S, Dewani J. A mobile team for screening of retinopathy of prematurity in India: Cost - effectiveness, outcomes, and impact assessment. *Taiwan J Ophthalmol*. 2017;7(3):155-159.

16. Mohammadi S-F, Rahban A, Darabeigi S, et al. Cost-effectiveness analysis of tele-retinopathy of prematurity screening in Iran. *Int J Ophthalmol*. 2021;14(4):560-566. doi:10.18240/ijo.2021.04.13

17. Moitry M, Zarca K, Granier M, et al. Effectiveness and efficiency of tele-expertise for improving access to retinopathy screening among 351 neonates in a secondary care center: An observational, controlled before-after study. *PLoS One*. 2018;13(10):e0206375.

18. van den Akker-van Marle ME, van Sorge AJ, Schalij-Delfos NE. Cost and effects of risk factor guided screening strategies for retinopathy of prematurity for different treatment strategies. *Acta Ophthalmol*. 2015;93(8):706-712.

19. Yanovitch TL, Siatkowski RM, McCaffree M, Corff KE. Retinopathy of prematurity in infants with birth weight>or=1250 grams-incidence, severity, and screening guideline cost-analysis. *J aapos*. 2006;10(2):128-134.

20. Zin AA, Magluta C, Pinto MF, et al. Retinopathy of prematurity screening and treatment cost in Brazil. *Rev Panam Salud Publica*. 2014;36(1):37-43.

21. Page MJ, McKenzie JE, Bossuyt PM, et al. The PRISMA 2020 statement: an updated guideline for reporting systematic reviews. *BMJ*. 2021;372:n71. doi:10.1136/bmj.n71

22. Cross KW. Cost of preventing retrolental fibroplasia? *Lancet*. 1973;2(7835):954-956. doi:10.1016/s0140-6736(73)92610-x

23. Boncz I, Kovács LG, Ertl T, Ágoston I, Molics B, Bódis J. Health-economic analysis of diseases related to disturbed neonatal adaptation: A cost of illness study. *Lege Artis Medicinae*. 2013;23(3-4):193-197.

24. Yu T-Y, Donovan T, Armfield N, Gole GA. Retinopathy of prematurity: the high cost of screening regional and remote infants. *Clin Exp Ophthalmol*. 2018;46(6):645-651. doi:10.1111/ceo.13160

25. Scholz SM, Greiner W. An exclusive human milk diet for very low birth weight newborns-A cost-effectiveness and EVPI study for Germany. *PLoS One*. 2019;14(12):e0226496. doi:10.1371/journal.pone.0226496

26. Zupancic JAF, Ying G-S, de Alba Campomanes A, Tomlinson LA, Binenbaum G, G-ROP Study Group. Evaluation of the economic impact of modified screening criteria for retinopathy of prematurity from the Postnatal Growth and ROP (G-ROP) study. *J Perinatol*. 2020;40(7):1100-1108. doi:10.1038/s41372-020-0605-5

27. Blencowe H, Lawn JE, Vazquez T, Fielder A, Gilbert C. Preterm-associated visual impairment and estimates of retinopathy of prematurity at regional and global levels for 2010. *Pediatr Res*. 2013;74(Suppl 1):35-49. doi:10.1038/pr.2013.205

28. Holmström G, Hellström A, Gränse L, et al. New modifications of Swedish ROP guidelines based on 10-year data from the SWEDROP register. *Br J Ophthalmol*. 2020;104(7):943-949. doi:10.1136/bjophthalmol-2019-314874

29. Trzcionkowska K, Groenendaal F, Andriessen P, et al. Risk Factors for Retinopathy of Prematurity in the Netherlands: A Comparison of Two Cohorts. *Neonatology*. 2021;118(4):462-469. doi:10.1159/000517247

30. Isaza G, Arora S. Incidence and severity of retinopathy of prematurity in extremely premature infants. *Canadian Journal of Ophthalmology*. 2012;47(3):296-300. doi:10.1016/j.jcjo.2012.03.027

For peer review only

31. Lundgren P, Jacobson L, Hård A-L, et al. High rate and large intercentre variability in retreatment of retinopathy of prematurity in infants born <24 gestational weeks. *BMJ Open Ophthalmol*. 2021;6(1):e000695. doi:10.1136/bmjophth-2020-000695

32. Rajan RP, Kannan NB, Sen S, et al. Clinico-demographic profile and outcomes of 25-gauge vitrectomy in advanced stage 5 retinopathy of prematurity. *Graefe's Archive for Clinical and Experimental Ophthalmology*. 2021;259(7):1695-1701. doi:10.1007/s00417-020-05063-2

33. Nicod E, Jackson TL, Grimaccia F, et al. Direct cost of pars plana vitrectomy for the treatment of macular hole, epiretinal membrane and vitreomacular traction: a bottom-up approach. *European Journal of Health Economics*. 2016;17(8):991-999. doi:10.1007/s10198-015-0741-6

34. Zhao D-Y, Zhang Y-J, He Z-J, et al. Clinical analysis of apnea after operation for retinopathy of prematurity. *Journal of Shanghai Jiaotong University (Medical Science)*. 2010;30(2):132-134.

35. Huang C-Y, Kuo R-J, Li C-H, et al. Prediction of visual outcomes by an artificial neural network following intravitreal injection and laser therapy for retinopathy of prematurity. *British Journal of Ophthalmology*. 2020;104(9):1277-1282. doi:10.1136/bjophthalmol-2019-314860

36. Köberlein J, Beifus K, Schaffert C, Finger RP. The economic burden of visual impairment and blindness: a systematic review. *BMJ Open*. 2013;3(11):e003471. doi:10.1136/bmjopen-2013-003471

For peer review only

PRISMA checklist

Section and Topic	Item #	Checklist item	Location where item is reported
**TITLE**			
Title	1	Identify the report as a systematic review.	Title
**ABSTRACT**			
Abstract	2	See the PRISMA 2020 for Abstracts checklist.	[See below]
**INTRODUCTION**			
Rationale	3	Describe the rationale for the review in the context of existing knowledge.	Page 3
Objectives	4	Provide an explicit statement of the objective(s) or question(s) the review addresses.	Page 3
**METHODS**			
Eligibility criteria	5	Specify the inclusion and exclusion criteria for the review and how studies were grouped for the syntheses.	Page 3
Information sources	6	Specify all databases, registers, websites, organisations, reference lists and other sources searched or consulted to identify studies. Specify the date when each source was last searched or consulted.	Page 3
Search strategy	7	Present the full search strategies for all databases, registers and websites, including any filters and limits used.	eTable 1
Selection process	8	Specify the methods used to decide whether a study met the inclusion criteria of the review, including how many reviewers screened each record and each report retrieved, whether they worked independently, and if applicable, details of automation tools used in the process.	Page 3
Data collection process	9	Specify the methods used to collect data from reports, including how many reviewers collected data from each report, whether they worked independently, any processes for obtaining or confirming data from study investigators, and if applicable, details of automation tools used in the process.	Page 3
Data items	10a	List and define all outcomes for which data were sought. Specify whether all results that were compatible with each outcome domain in each study were sought (e.g. for all measures, time points, analyses), and if not, the methods used to decide which results to collect.	eTable 2
	10b	List and define all other variables for which data were sought (e.g. participant and intervention characteristics, funding sources). Describe any assumptions made about any missing or unclear information.	eTable 2
Study risk of bias assessment	11	Specify the methods used to assess risk of bias in the included studies, including details of the tool(s) used, how many reviewers assessed each study and whether they worked independently, and if applicable, details of automation tools used in the process.	Page 3
Effect measures	12	Specify for each outcome the effect measure(s) (e.g. risk ratio, mean difference) used in the synthesis or presentation of results.	Page 3
Synthesis methods	13a	Describe the processes used to decide which studies were eligible for each synthesis (e.g. tabulating the study intervention characteristics and comparing against the planned groups for each synthesis (item #5)).	Page 3
	13b	Describe any methods required to prepare the data for presentation or synthesis, such as handling of missing summary statistics, or data conversions.	Page 3
	13c	Describe any methods used to tabulate or visually display results of individual studies and syntheses.	Page 3

For peer review only

Section and Topic	Item #	Checklist item	Location where item is reported
	13d	Describe any methods used to synthesize results and provide a rationale for the choice(s). If meta-analysis was performed, describe the model(s), method(s) to identify the presence and extent of statistical heterogeneity, and software package(s) used.	Page 3
	13e	Describe any methods used to explore possible causes of heterogeneity among study results (e.g. subgroup analysis, meta-regression).	Page 3
	13f	Describe any sensitivity analyses conducted to assess robustness of the synthesized results.	Page 3
Reporting bias assessment	14	Describe any methods used to assess risk of bias due to missing results in a synthesis (arising from reporting biases).	Not possible
Certainty assessment	15	Describe any methods used to assess certainty (or confidence) in the body of evidence for an outcome.	Page 4
**RESULTS**			
Study selection	16a	Describe the results of the search and selection process, from the number of records identified in the search to the number of studies included in the review, ideally using a flow diagram.	eFigure 1
	16b	Cite studies that might appear to meet the inclusion criteria, but which were excluded, and explain why they were excluded.	eTable 4
Study characteristics	17	Cite each included study and present its characteristics.	Page 3
Risk of bias in studies	18	Present assessments of risk of bias for each included study.	eTable 3
Results of individual studies	19	For all outcomes, present, for each study: (a) summary statistics for each group (where appropriate) and (b) an effect estimate and its precision (e.g. confidence/credible interval), ideally using structured tables or plots.	Page 4
Results of syntheses	20a	For each synthesis, briefly summarise the characteristics and risk of bias among contributing studies.	Page 4-5
	20b	Present results of all statistical syntheses conducted. If meta-analysis was done, present for each the summary estimate and its precision (e.g. confidence/credible interval) and measures of statistical heterogeneity. If comparing groups, describe the direction of the effect.	Figure 3
	20c	Present results of all investigations of possible causes of heterogeneity among study results.	Figures 1-3
	20d	Present results of all sensitivity analyses conducted to assess the robustness of the synthesized results.	Not applicable
Reporting biases	21	Present assessments of risk of bias due to missing results (arising from reporting biases) for each synthesis assessed.	Not possible
Certainty of evidence	22	Present assessments of certainty (or confidence) in the body of evidence for each outcome assessed.	Table 2 and Figure 3
**DISCUSSION**			
Discussion	23a	Provide a general interpretation of the results in the context of other evidence.	Page 5-6
	23b	Discuss any limitations of the evidence included in the review.	Page 5-6

For peer review only

Section and Topic	Item #	Checklist item	Location where item is reported
	23c	Discuss any limitations of the review processes used.	Page 5
	23d	Discuss implications of the results for practice, policy, and future research.	Page 6
**OTHER INFORMATION**			
Registration and protocol	24a	Provide registration information for the review, including register name and registration number, or state that the review was not registered.	Page 2
	24b	Indicate where the review protocol can be accessed, or state that a protocol was not prepared.	Page 3
	24c	Describe and explain any amendments to information provided at registration or in the protocol.	Not applicable
Support	25	Describe sources of financial or non-financial support for the review, and the role of the funders or sponsors in the review.	Page 7
Competing interests	26	Declare any competing interests of review authors.	Page 7
Availability of data, code and other materials	27	Report which of the following are publicly available and where they can be found: template data collection forms; data extracted from included studies; data used for all analyses; analytic code; any other materials used in the review.	Page 7

For peer review only

PRISMA abstract checklist

Section and Topic	Item #	Checklist item	Reported (Yes/No)
**TITLE**			
Title	1	Identify the report as a systematic review.	Title
**BACKGROUND**			
Objectives	2	Provide an explicit statement of the main objective(s) or question(s) the review addresses.	Objective
**METHODS**			
Eligibility criteria	3	Specify the inclusion and exclusion criteria for the review.	Study selection
Information sources	4	Specify the information sources (e.g. databases, registers) used to identify studies and the date when each was last searched.	Data sources
Risk of bias	5	Specify the methods used to assess risk of bias in the included studies.	Data Extraction and Synthesis
Synthesis of results	6	Specify the methods used to present and synthesise results.	Data Extraction and Synthesis
**RESULTS**			
Included studies	7	Give the total number of included studies and participants and summarise relevant characteristics of studies.	Results
Synthesis of results	8	Present results for main outcomes, preferably indicating the number of included studies and participants for each. If meta-analysis was done, report the summary estimate and confidence/credible interval. If comparing groups, indicate the direction of the effect (i.e. which group is favoured).	Results
**DISCUSSION**			
Limitations of evidence	9	Provide a brief summary of the limitations of the evidence included in the review (e.g. study risk of bias, inconsistency and imprecision).	Conclusions and Relevance
Interpretation	10	Provide a general interpretation of the results and important implications.	Conclusions and Relevance
**OTHER**			
Funding	11	Specify the primary source of funding for the review.	[In funding statement]
Registration	12	Provide the register name and registration number.	Registration

For peer review only

Section and Topic	Item #	Checklist item	Reported (Yes/No)
			number in PROSPERO

For peer review only

BMJ Open

Costs Associated with Retinopathy of Prematurity: A Systematic Review and Meta-analysis

Journal:	*BMJ Open*
Manuscript ID	bmjopen-2021-057864.R1
Article Type:	Original research
Date Submitted by the Author:	05-Sep-2022
Complete List of Authors:	Gyllensten, Hanna; University of Gothenburg Institute of Health and Care Sciences; University of Gothenburg Centre for Person-Centred Care<br>Humayun, Jhangir; University of Gothenburg Institute of Health and Care Sciences; University of Gothenburg Centre for Person-Centred Care<br>Sjöbom, Ulrika; University of Gothenburg Institute of Health and Care Sciences; University of Gothenburg, Department of Clinical Neuroscience<br>Hellström, Ann; University of Gothenburg Institute of Neuroscience and Physiology, Neuroscience<br>Löfqvist, Chatarina; University of Gothenburg Institute of Health and Care Sciences, Sahlgrenska Academy; University of Gothenburg Institute of Neuroscience and Physiology, Department of Clinical Neuroscience
**Primary Subject Heading**:	Ophthalmology
Secondary Subject Heading:	Health economics, Ophthalmology, Paediatrics
Keywords:	HEALTH ECONOMICS, Paediatric ophthalmology < OPHTHALMOLOGY, Medical retina < OPHTHALMOLOGY

SCHOLARONE™
Manuscripts

I, the Submitting Author has the right to grant and does grant on behalf of all authors of the Work (as defined in the below author licence), an exclusive licence and/or a non-exclusive licence for contributions from authors who are: i) UK Crown employees; ii) where BMJ has agreed a CC-BY licence shall apply, and/or iii) in accordance with the terms applicable for US Federal Government officers or employees acting as part of their official duties; on a worldwide, perpetual, irrevocable, royalty-free basis to BMJ Publishing Group Ltd ("BMJ") its licensees and where the relevant Journal is co-owned by BMJ to the co-owners of the Journal, to publish the Work in this journal and any other BMJ products and to exploit all rights, as set out in our licence.

The Submitting Author accepts and understands that any supply made under these terms is made by BMJ to the Submitting Author unless you are acting as an employee on behalf of your employer or a postgraduate student of an affiliated institution which is paying any applicable article publishing charge ("APC") for Open Access articles. Where the Submitting Author wishes to make the Work available on an Open Access basis (and intends to pay the relevant APC), the terms of reuse of such Open Access shall be governed by a Creative Commons licence – details of these licences and which Creative Commons licence will apply to this Work are set out in our licence referred to above.

Other than as permitted in any relevant BMJ Author's Self Archiving Policies, I confirm this Work has not been accepted for publication elsewhere, is not being considered for publication elsewhere and does not duplicate material already published. I confirm all authors consent to publication of this Work and authorise the granting of this licence.

For peer review only

Title page

Costs Associated with Retinopathy of Prematurity: A Systematic Review and Meta-analysis

Authors

Hanna Gyllensten[1,2] Associate professor

Jhangir Humayun[1,2], B.Sc.

Ulrika Sjöbom[1,3], M.Sc.

Ann Hellström[3] Professor

Chatarina Löfqvist[1,2,3] Associate professor

Author affiliations

[1] Institute of Health and Care Sciences, Sahlgrenska Academy, University of Gothenburg, Box 457, SE-405 30 Gothenburg, Sweden.

[2] University of Gothenburg Centre for Person-Centred Care (GPCC), Sahlgrenska Academy, University of Gothenburg, Box 457, SE-405 30 Gothenburg, Sweden.

[3] Department of Clinical Neuroscience, Institute of Neuroscience and Physiology, Sahlgrenska Academy, University of Gothenburg, Gothenburg, Sweden;

Contact, corresponding author

Hanna Gyllensten, Institute of Health and Care Sciences, Sahlgrenska Academy, University of Gothenburg, Postal Address: Box 457, SE-405 30 Gothenburg, Sweden, E-mail: hanna.gyllensten@gu.se. Phone: +46-(0)70-748 24 12

Keywords

Retinopathy of Prematurity; Costs and Cost Analysis; Systematic Review; Meta-Analysis.

Word count (excluding title page, abstract, references, figures and tables): 2644

For peer review only

For peer review only - http://bmjopen.bmj.com/site/about/guidelines.xhtml

Abstract

Background: To review and analyze evidence regarding costs for retinopathy of prematurity (ROP) screening, lifetime costs and resource use among infants born preterm who develop ROP, and how these costs have developed over time in different regions.

Methods: Included studies presented costs for ROP screening and the lifetime costs (including laser treatment and follow-up costs) and resource use among people who develop ROP. Studies not reporting on cost calculation methods or ROP-specific costs were excluded. Included studies were further searched to identify eligible references and citations. PubMed and Scopus from inception to June 23, 2021. Two independent reviewers screened for inclusion and extracted data, including items from a published checklist for quality assessment used for bias assessment, summary, and meta-analysis for treatment costs.

Results: In total, 15 studies reported ROP screening costs, and 13 reported lifetime costs (either treatment and/or follow-up costs) for infants with ROP. The range for screening costs (10 studies) was US$5–$253 per visit, or US$324–$1072 per screened child (5 studies). Costs for treatment (11 studies) ranged from US$38 to US$6500 per child. Four studies reported healthcare follow-up costs (lifetime costs ranging from US$64–US$2420, and 10 year-costs of US$1695, respectively), and of these, three also reported lifetime costs for blindness (range US$26,686–US$224,295) using secondary cost data.

Discussion: Included papers largely followed the quality assessment checklist items, thus indicating a low risk of bias. The costs of screening for and treating ROP are small compared to the societal costs of resulting blindness. However, little evidence is available for predicting the effects of changes in patient population, screening schedule, or ROP treatments.

For peer review only - http://bmjopen.bmj.com/site/about/guidelines.xhtml

Other: Primary sources of funding: the Swedish Research Council and the University of Gothenburg Centre for Person-Centred Care. PROSPERO registration: CRD42020208213

Strengths and limitations of this study

- To our knowledge, this is the first systematic review or meta-analysis of Retinopathy of Prematurity costs.
- PubMed and Scopus were searched systematically, and manual search of reference lists and citations of the identified papers did not identify any additional studies, thus indicating that the database search had good coverage of the topic of investigation.
- The main limitations of this work were the exclusion of grey literature and the lack of analyses of publication bias for the meta-analysis.

For peer review only - http://bmjopen.bmj.com/site/about/guidelines.xhtml

Introduction

Improvements in neonatal care have resulted in increased survival among children born preterm,[1] but these infants are at risk of developing preterm-related morbidities such as retinopathy of prematurity (ROP). ROP is characterized by abnormal neurovascular development and, in its worst forms, retinal detachment and blindness.[2] Although preventable, ROP is the leading cause of blindness in children worldwide,[3] a ranking associated with the survival of infants with extremely low gestational age and birth weight in some parts of the world, and use of unmonitored treatments with 100% oxygen in other regions.[2]

ROP management and treatment economics are still challenging in many health systems because of screening-associated costs, patient-related costs, and medico-legal liability.[4] Thus, there is an urgent need for more concerted efforts to guide healthcare providers in how to use resources efficiently, both in developing economies during a phase of improving survival of preterm infants, such as in many parts of Africa [5], and in countries like Sweden with major neonatal morbidities still affecting a large proportion of those who survive.[6]

Here we present an overview of costs associated with ROP screening and treatment, examining the evidence related to costs for ROP screening and lifetime costs (including laser treatment and follow-up costs) and resource use among infants born preterm who develop ROP. We also examine the trajectories of these costs over time in different regions in a meta-analysis.

Methods

This work followed the Preferred Reporting Items for Systematic Review and Meta-Analysis (i.e., PRISMA),[7] with protocol available in PROSPERO (reference CRD42020208213).[8]

For peer review only

Article search

Pubmed and Scopus were searched (eTable 1, 23 Jun 2021) to identify original research on costs for ROP, including full cost or cost increases associated with ROP, without restricting language, publication date, or country. Papers were thus included if presenting costs for ROP screening or lifetime costs (including laser treatment and follow-up costs) and resource use among people who develop ROP. Lifetime costs can for example include follow-up healthcare costs but also productivity loss due to blindness or other cost components occurring due to visual impairment later in life. Articles that did not describe the cost calculation method were excluded, as were those not presenting the costs for the group with ROP separately.

Rayyan QCRI was used for handling duplicates and the selection of studies for inclusion. Two independent reviewers (JH and CL or HG) searched the databases, screened articles for eligibility, extracted data using a pre-specified data extraction sheet (eTable 2), and hand-searched included studies (7 July 2021) to identify eligible references and citations. Conflicting views were resolved by discussion with a third reviewer (CL or HG).

The data extraction sheet included items (eTable 2) from a published checklist for quality assessment of economic evaluations[9] including a core set of items relevant in assessing the risk of bias in included studies. The 19 checklist items covers design and methods, population and generalizability, as well as ethics and funding, answered as yes or no during the assessment. To aid reading, summary scores indicating the items answered as Yes for each paper were calculated, thus a high summary score indicates that many of the items were covered. Quality of evidence was rated on a scale from 1 to 5 for individual articles, according to: 1=e.g., properly powered randomized controlled trials; 2=e.g., prospective cohort studies; 3=e.g., retrospective cohort studies; 4=case series with or without intervention or cross-sectional study; 5=e.g., opinion of respected authorities.[10]

For peer review only

Analysis

Conventional screening (excluding telemedicine costs), laser treatment, and long-term follow-up costs were reported, respectively, accounting for ROP severity and differences over time and between countries. Identified costs were adjusted to 2020 US dollars (US$) using annual exchange rates[11] and the Organisation for Economic Co-operation and Development inflation factor.[12] After imputation of missing variance based on the percentage variance in studies presenting such information, treatment costs were summarized in a forest plot, by year and subgroups using the World Bank country classification based on gross national income per capita,[13] as cost levels can be expected to differ.

Patient and public involvement

This project did not include patient or public involvement in developing the research questions, design, conduct, choice of outcome measures, or recruitment.

Results

Of the 503 studies screened after duplicates from the databases were removed, 123 were assessed for eligibility based on full text, and 19 studies were included in the synthesis of results (eFigure 1). Reasons for exclusion were absence of data on costs associated with ROP, lack of original data, or inclusion of data related only to insurance payments or litigation. No additional studies were identified by a hand search of references and a Scopus search of citations of included studies. An overview of all included studies[14–32] is presented in Table 1, including references to secondary cost sources.[33–39] In total, 15 studies covered screening costs and 13 reported lifetime costs (treatment and/or follow-up costs) for infants who developed ROP.

For peer review only - http://bmjopen.bmj.com/site/about/guidelines.xhtml

For peer review only

Twelve studies were conducted in high-income economies: seven in the United States, two in Canada, and one each in the United Kingdom, Netherlands, and France. Three studies were conducted in upper-middle income economies: one each in Peru, Thailand, and Brazil. Three studies were conducted in lower-middle income economies: two in India and one in Iran. One study was conducted in both the United States and Mexico (Table 1). All studies reported the economic analyses using either US dollars, euros, or local currency. The patient populations in all studies were infants at risk for ROP, although the studies used different inclusion criteria based on gestational age at birth and birth weight. In addition, the ROP definition for stages and treatment criteria varied with the timing of the study and international guidelines for classification at that time.

Risk of bias in included studies

The quality assessment indicated a high overall quality of the included studies (eTable 3), with 16 of 19 of them fulfilling at least 16 of the assessed criteria. However, eight studies did not fulfill the criteria for discounting future costs and outcomes or for subjecting results to sensitivity analyses to address the effects of assumptions. Additionally, 14 studies met criteria regarding the reporting of incremental analysis and potential conflicts of interest. Thus, overall, the assessment suggested a low risk of bias in the included papers, and also indicated where lack of reporting on potential conflicts of interest was most problematic. Quality of evidence ranged from 1 to 5 for individual articles, with articles most commonly based on data from retrospective cohort studies (evidence rating 3; 9 publications).

Costs for ROP screening

Studies reporting costs related to screening had different designs: six were retrospective cohort studies using medical chart review or register data,[15,16,20,24,28,30] nine developed

For peer review only - http://bmjopen.bmj.com/site/about/guidelines.xhtml

economic models,[19,21,23,25–27,29,31,32] and two were public intervention studies related to the introduction of ROP screening programs.[17,18] Although the assessment indicated a low risk of bias, screening costs differed substantially among reporting countries (Figure 1a).

Costs for routine ROP screening, excluding transportation costs, are reported in Table 2. Ten studies reported a mean unit cost per screening of US$137 (range: 5–253). In addition, five studies reported a mean cost per screened child of US$553 (range: 324–1072). Of these, two studies reported comparably low costs[21,23] for staff and equipment, whereas Rothchild et al.[19] reported comparably higher costs in the US setting. One study also included transportation costs,[15] and when these costs were removed, screening cost was comparably low. The other studies reported similar costs for screening per child (range: US$324–$602).[25,28,29]

Javitt et al.[32] reported a mean unit cost of US$183 for a first screening and of US$149 for follow-up screening, whereas Lee et al.[30] reported a mean unit cost of US$112 for screening one eye. Finally, two studies from India[17,18] reported screening costs of US$1003 and US$630, respectively, for identifying one child with ROP.

In studies comparing alternative screening or treatment options, no common comparator was identified. The incremental cost reported in Black et al.[22] indicated a savings associated with higher gestational age at birth (Table 1). Jackson et al.[27] used economic modeling to estimate the cost-utility of ROP screening using telemedicine vs. conventional ROP screening. Javitt et al.[32] used modeling to compare weekly, biweekly, or monthly screening.

Costs for ROP treatment

In all, 14 studies reported costs related to the laser treatment of ROP (Figure 1b). Four studies of treatment costs were retrospective cohort studies,[20,24,28,30] eight were modeling

For peer review only - http://bmjopen.bmj.com/site/about/guidelines.xhtml

studies,[14,19,21,23,25,26,29,31] and two were public intervention studies.[17,18] In addition, two of the included studies[31,32] reported costs for cryotherapy (not included in the analyses below). Eleven studies reported total treatment costs per child, at a mean US$2442 (range: 38–6500). Castillo-Riquelme et al.[29] found unilateral treatment costs up to US$1165 and bilateral treatment costs up to US$1514, based partially on secondary data from Brown et al.[31] Two studies[20,26] cited unit costs of laser treatment of US$4065 and US$5661, respectively. Laser treatment costs are reported in Table 2. Dave et al.[24] described costs for screening and treatment combined (US$2962) in a cohort of children with blindness.

Accounting for the low assessed risk of bias but large expected variation based on cost-levels of individual countries, the meta-analysis by country classification (Figures 2-3) estimated the average costs in high-income economies to US$2960 (95% confidence interval [CI]: 2003–3917). Corresponding figures were US$329 (95% CI: 9–649) in upper-middle–income economies and US$3692 (95% CI: 670–6715) in lower-middle–income economies, respectively. Most studies did not report variance of results, making publication bias analysis unfeasible. However, model diagnostics (I^2 and Cochrane Q) indicated high heterogeneity between studies within each country classification, which suggests that the results from the meta-analysis should be interpreted with caution.

Follow-up costs and resource use among infants born preterm and developing ROP

Only four studies reported follow-up costs occurring after screening and treatment, and although the risk of bias was assessed as low, the reported results largely differed between studies. Castillo-Riquelme et al.[29] reported healthcare follow-up costs over 10 years of up to US$1695. Dave et al.[24] reported a lifetime follow-up visit cost of US$64 and a blindness cost of US$146,952. Rothchild et al.[19] reported lifetime follow-up healthcare costs of US$1681 (US) and US$2420 (Mexico), whereas the costs for blindness were estimated to be

For peer review only - http://bmjopen.bmj.com/site/about/guidelines.xhtml

US$92,460 (US) and US$26,686 (Mexico). Wongwai et al.[21] reported the lifetime costs of blindness to be $224,295. In addition, Black et al.[22] reported the costs per quality-adjusted life-year (QALY) associated with ROP and other comorbidities associated with being born preterm.

Discussion

The studies we identified could be grouped by whether they reported costs for screening, costs for treatment, or costs (and QALYs) during long-term follow-up or even from a lifetime perspective. The cost range per ROP screening was US$5–$253 per visit, or US$324–$1072 per screened child. Costs for ROP treatment ranged from US$38–$6500 per child. In addition, four studies reported healthcare follow-up costs, and three reported lifetime costs using secondary data on costs for blindness. Although quality assessment indicated a low risk of bias, comparisons between studies were challenging because of the lack of detailed cost and resource use data.

To our knowledge, this is the first systematic review of ROP costs. Included papers largely followed the quality assessment checklist items of a commonly used tool,[40] thus indicating a low risk of bias. However, few of the included articles reported disaggregated cost and resource use data or detailed the included cost components, as is recommended for economic evaluations.[41] The main limitations of this work were the exclusion of grey literature and the lack of analyses of publication bias for the meta-analysis. Guidance for reliability in systematic reviews of retinal disorder interventions[42] was fulfilled, but the standards for systematic reviews of costs and cost-effectiveness studies were not due to the lack of grey literature assessment.[43] Also, since costs were reported purely in a descriptive manner no sensitivity analyses were conducted for alternative categorizations of cost components or country classifications. While not a limitation specific to this analysis but

For peer review only - http://bmjopen.bmj.com/site/about/guidelines.xhtml

rather of the lack of variance information in the included papers, the findings from the meta-analysis of treatment costs needs to be interpreted with caution after variance was imputed. This lack of variance information also made meta-analysis of screening costs unattainable, since no basis for imputation was available. Moreover, the search strategy and databases are expected to cover largely English-language literature and was limited to only two databases, but the reference and citation search yielded no additional studies to include. We thus expect our findings to represent a good overview of the available evidence, and that regardless the reservations associated with the meta-analysis to represent current knowledge about costs related to screening and treatment of ROP.

Cost components for ROP screening included staff salaries/time, equipment and maintenance, supplies, and staff training. Screening costs for ROP were low compared to other associated costs and, with few exceptions, of the same order of magnitude in the included studies. Exceptions were probably attributable to salary differences.

Screening access and schedules vary between countries.[44] With the possible exception of Javitt et al.,[32], the included studies provided little evidence for how case-mix and alternative screening schedules affect costs for screening. Savings are expected, however, and a modeling study using published cost data calculated an annual cost savings from reduced screening of US$3 million in the United States.[45] However, with low screening costs, the main benefit is reduced discomfort for the infants and reduced travel costs (which can be substantial[15]). The most considerable potential for savings on screening is probably increasing gestational age. US data indicate that ROP frequency increased over time, particularly in infants born very preterm,[46] and infants of lower gestational age usually both require more screening visits and have more severe ROP.[47] Potential savings have been reported from screening using telemedicine (compared to transporting infants to a specialized

For peer review only - http://bmjopen.bmj.com/site/about/guidelines.xhtml

hospital),[15] or using bedside screening with mobile equipment instead of moving the infants to a specific screening facility[48]; however, this review did not consider these aspects.

Treatment costs were low compared to the costs for follow-up, with Brazil, Mexico, and Peru having substantially lower treatment costs than the other countries. Both Javitt et al.[32] and Brown et al.[31] reported low costs for the historically used cryo treatment, at approximately 63% of that for laser treatment. For laser treatment, the cost range was US$2304–$6864 per treated child. None of the studies included the more recent anti-vascular endothelial growth factor (VEGF) therapy. Moreover, no study reported costs based on ROP stages, age of treated infants, or plus disease status.[49] Thus, studies provide little guidance on how treatment costs will develop over time as more infants of lower gestational age survive.

Variation among studies in whether one or two eyes were treated made comparisons less relevant, which may reflect the unilateral schedule used in the historically influential Cryo-ROP study.[50] However, Swedish registers indicate that bilateral treatment is common (76% of initial treatments and 97% overall)[47] and that retreatment is more frequent among infants with very low gestational age[51] and those treated exclusively with anti-VEGF.[47]

When examining ROP treatment, cost components included staff salaries/time, equipment and maintenance, supplies, and staff training. Sometimes anesthesia costs were reported separately or excluded. Transportation was also a considerable cost component in relation to treatment.[20] Other potential costs that were not measured include those for the added time spent in hospital or intensive care, including parental leave, during treatment. Many studies reported only total charges, which are expected to be higher than costs to the healthcare provider. However, use of charges as opposed to costs was not an obvious cause of variation here. Two studies from India[17,18] reported high costs compared to other studies of both costs and charges, possibly because of some transportation costs remaining as part of

For peer review only - http://bmjopen.bmj.com/site/about/guidelines.xhtml

additional components. Thus the apparent decrease in costs over time in the lower-middle-income economies seen in the meta-analysis should be interpreted with caution.

Although ROP results in high costs throughout life, this outcome is primarily based on secondary data for blindness. As the leading cause of preventable childhood blindness[52] and probably the leading cause of childhood blindness in middle-income countries,[53] ROP should be associated with much of the estimated costs of blindness. Moreover, it has been argued that costs for blindness do not differ by cause.[54] Little evidence was available on follow-up after successful, or partially successful, treatment of ROP. Dave et al.[24] indicated three healthcare visits over the first 7 years of life, whereas Castillo-Riquelme et al.[29] did not differentiate visits based on treatment or ROP stage. Rothchild et al. included transportation costs, white canes, Braille equipment, and supplies,[19] but disregarded other costs among children retaining sight. Thus, although costs differ by the severity of visual impairment,[55] studies of ROP costs do not tend to report this more detailed level of sight. The current knowledge does not inform potential savings or inform subsidy decisions for ROP treatment developments that can save a little more sight. Taken together, the short follow-up underestimates the total impact of blindness,[56] and not accounting for visual impairment results in underestimating the financial impact of ROP.

There is a need for comprehensive knowledge about the costs of ROP, both during the introduction of new ROP screening programs and in countries with established programs that are now redistributing resources to handle the increasing survival of very preterm infants with high disease burden. In addition to relevant cost components of ROP (eFigure 2), complementary studies of the benefits of various neonatal preventative strategies, including oxygen delivery, are warranted because evidence of the costs resulting from conditions such as bronchopulmonary dysplasia is also lacking.[57] Such studies should follow state-of-the-art methods for conduct and reporting of health economic studies.

For peer review only - http://bmjopen.bmj.com/site/about/guidelines.xhtml

Conclusions

Although costs of screening and treating ROP are substantial for health systems, they are small compared to the follow-up costs to society of resulting blindness. However, little evidence is available to support predictions about the consequences of changes in the patient population, screening schedule, or treatment regimens for ROP.

For peer review only

For peer review only - http://bmjopen.bmj.com/site/about/guidelines.xhtml

FUNDING STATEMENT

HG was financed by the Swedish Research Council (#2016-01131). JH was financed by the University of Gothenburg Centre for Person-Centred Care (GPCC), a teaching assistant program. AH was supported by The Wallenberg Clinical Scholars, The Swedish Research Council (#2020-01092), the Gothenburg County Council (ALF project, #426531), the Gothenburg Medical Society, and De Blindas Vänner, and CL was financed by the GPCC. The funders had no role in the design of the study or writing of the protocol.

COMPETING INTERESTS STATEMENT

HG is employed part-time by IQVIA, which is a contract research organization that performs commissioned pharmacoepidemiological studies. Thus, its employees have been and currently are working in collaboration with several pharmaceutical companies. JH reports no competing interests. AH holds stock/stock options in Premalux AB and has received consulting fees from Takeda Inc. CL holds stocks in Premalux AB.

CONTRIBUTIONS

All authors contributed to the design of the study. HG, JH, and CL designed the database search and data extraction methods. JH and CL undertook the literature search, assessed studies for eligibility, and extracted data. In case of disagreement, assessments were made in discussion with HG. AH contributed clinical expertise on preterm infants and morbidity. HG, JH, US, and CL discussed the data and interpreted the results. HG, JH, and CL drafted the manuscript. All authors critically reviewed and approved the final manuscript. HG had full access to all the data in the study and takes responsibility for the integrity of the data and the accuracy of the data analysis.

ACKNOWLEDGEMENTS

Thanks to SF Edit, a professional scientific-editing service, for language editing.

For peer review only - http://bmjopen.bmj.com/site/about/guidelines.xhtml

DATA AVAILABILITY STATEMENT

All data relevant to the study are included in the article. Original data are available in the reviewed publications, which are all cited. Additional data from the data extraction performed are available on reasonable request from the corresponding author, including author template data collection forms, data extracted from included studies, data used for all analyses, analytic code, and any other materials used in the review.

ETHICS APPROVAL STATEMENT

Not applicable.

For peer review only

For peer review only - http://bmjopen.bmj.com/site/about/guidelines.xhtml

References

1. Lawn JE, Davidge R, Paul VK, et al. Born too soon: care for the preterm baby. *Reprod Health*. 2013;10 Suppl 1:S5. doi:10.1186/1742-4755-10-S1-S5

2. Hellström A, Smith LEH, Dammann O. Retinopathy of prematurity. *Lancet*. 2013;382(9902):1445-1457. doi:10.1016/S0140-6736(13)60178-6

3. Yonekawa Y, Thomas BJ, Thanos A, et al. THE CUTTING EDGE OF RETINOPATHY OF PREMATURITY CARE: Expanding the Boundaries of Diagnosis and Treatment. *Retina (Philadelphia, Pa)*. 2017;37(12):2208-2225. doi:10.1097/IAE.0000000000001719

4. Moshfeghi DM, Capone A. Economic Barriers in Retinopathy of Prematurity Management. *Ophthalmol Retina*. 2018;2(12):1177-1178. doi:10.1016/j.oret.2018.10.002

5. Dupe S. Ademola-Popoola, Tunji S. Oluleye. Retinopathy of Prematurity (ROP) in a Developing Economy with Improving Health Care | SpringerLink. *Current Ophtalmology Reports*. 2017;5:114-118. doi:https://doi-org.ezproxy.ub.gu.se/10.1007/s40135-017-0129-0

6. Norman M, Hallberg B, Abrahamsson T, et al. Association Between Year of Birth and 1-Year Survival Among Extremely Preterm Infants in Sweden During 2004-2007 and 2014-2016. *JAMA*. 2019;321(12):1188-1199. doi:10.1001/jama.2019.2021

7. Page MJ, McKenzie JE, Bossuyt PM, et al. The PRISMA 2020 statement: an updated guideline for reporting systematic reviews. *BMJ*. 2021;372:n71. doi:10.1136/bmj.n71

8. PROSPERO International prospective register of systematic reviews. NIHR, National INstitute for Health Research. https://www.crd.york.ac.uk/prospero/

9. Evers S, Goossens M, de Vet H, van Tulder M, Ament A. Criteria list for assessment of methodological quality of economic evaluations: Consensus on Health Economic Criteria. *Int J Technol Assess Health Care*. 2005;21(2):240-245.

10. Oxford Centre for Evidence-Based Medicine: Levels of Evidence (March 2009). Centre for Evidence-Based Medicine (CEBM), University of Oxford. Accessed August 30, 2022. https://www.cebm.ox.ac.uk/resources/levels-of-evidence/oxford-centre-for-evidence-based-medicine-levels-of-evidence-march-2009

11. Annual average exchange rates (aggregate). Sveriges Riksbank. Accessed June 8, 2021. https://www.riksbank.se/en-gb/statistics/search-interest--exchange-rates/annual-average-exchange-rates/

12. Prices - Inflation (CPI) - OECD Data. Organisation for Economic Co-operation and Development, OECD. Accessed June 9, 2021. http://data.oecd.org/price/inflation-cpi.htm

13. World Bank Country and Lending Groups – World Bank Data Help Desk. Accessed May 13, 2021. https://datahelpdesk.worldbank.org/knowledgebase/articles/906519-world-bank-country-and-lending-groups

14. Mohammadi SF, Rahban A, Darabeigi S, et al. Cost-effectiveness analysis of tele-retinopathy of prematurity screening in Iran. *Int J Ophthalmol*. 2021;14(4):560-566. doi:10.18240/ijo.2021.04.13

15. Moitry M, Zarca K, Granier M, et al. Effectiveness and efficiency of tele-expertise for improving access to retinopathy screening among 351 neonates in a secondary care center: An observational, controlled before-after study. *PLoS One*. 2018;13(10):e0206375.

16. Isaac M, Isaranuwatchai W, Tehrani N. Cost analysis of remote telemedicine screening for retinopathy of prematurity. *Can J Ophthalmol*. 2018;53(2):162-167.

For peer review only - http://bmjopen.bmj.com/site/about/guidelines.xhtml

17. Kelkar J, Agashe S, Kelkar A, Kh, ekar R. Mobile unit for retinopathy of prematurity screening and management at urban Neonatal Intensive Care Units: Outcomes and impact assessment. *Oman J Ophthalmol*. 2017;10(1):13-16.

18. Kelkar J, Kelkar A, Sharma S, Dewani J. A mobile team for screening of retinopathy of prematurity in India: Cost - effectiveness, outcomes, and impact assessment. *Taiwan J Ophthalmol*. 2017;7(3):155-159.

19. Rothschild MI, Russ R, Brennan KA, et al. The Economic Model of Retinopathy of Prematurity (EcROP) Screening and Treatment: Mexico and the United States. *Am J Ophthalmol*. 2016;168:110-121.

20. van den Akker-van Marle ME, van Sorge AJ, Schalij-Delfos NE. Cost and effects of risk factor guided screening strategies for retinopathy of prematurity for different treatment strategies. *Acta Ophthalmol*. 2015;93(8):706-712.

21. Wongwai P, Kingkaew P, Asawaphureekorn S, Kolatat T. A store-and-forward telemedicine for retinopathy of prematurity screen: Is it cost-effective in Thailand? *Asian Biomedicine*. 2015;9(5):665-673.

22. Black L, Hulsey T, Lee K, Parks DC, Ebeling MD. Incremental Hospital Costs Associated With Comorbidities of Prematurity. *Manag Care*. 2015;24(12):54-60.

23. Zin AA, Magluta C, Pinto MF, et al. Retinopathy of prematurity screening and treatment cost in Brazil. *Rev Panam Salud Publica*. 2014;36(1):37-43.

24. Dave HB, Gordillo L, Yang Z, Zhang MS, Hubbard GB 3rd, Olsen TW. The societal burden of blindness secondary to retinopathy of prematurity in Lima, Peru. *Am J Ophthalmol*. 2012;154(4):750-755.

25. Dunbar JA, Hsu V, Christensen M, Black B, Williams P, Beauchamp G. Cost-utility analysis of screening and laser treatment of retinopathy of prematurity. *Journal of AAPOS*. 2009;13(2):186-190.

26. Kamholz KL, Cole CH, Gray JE, Zupancic JA. Cost-effectiveness of early treatment for retinopathy of prematurity. *Pediatrics*. 2009;123(1):262-269.

27. Jackson KM, Scott KE, Graff Zivin J, et al. Cost-utility analysis of telemedicine and ophthalmoscopy for retinopathy of prematurity management. *Arch Ophthalmol*. 2008;126(4):493-499.

28. Yanovitch TL, Siatkowski RM, McCaffree M, Corff KE. Retinopathy of prematurity in infants with birth weight>or=1250 grams-incidence, severity, and screening guideline cost-analysis. *J aapos*. 2006;10(2):128-134.

29. Castillo-Riquelme MC, Lord J, Moseley MJ, Fielder AR, Haines L. Cost-effectiveness of digital photographic screening for retinopathy of prematurity in the United Kingdom. *Int J Technol Assess Health Care*. 2004;20(2):201-213.

30. Lee SK, Norm, C., et al. Evidence for changing guidelines for routine screening for retinopathy of prematurity. *Arch Pediatr Adolesc Med*. 2001;155(3):387-395.

31. Brown GC, Brown MM, Sharma S, Tasman W, Brown HC. Cost-effectiveness of treatment for threshold retinopathy of prematurity. *Pediatrics*. 1999;104(4):e47.

32. Javitt J, Cas RD, Chiang YP. Cost-effectiveness of screening and cryotherapy for threshold retinopathy of prematurity. *Pediatrics*. 1993;91(5):859-866.

33. Frick KD, Foster A. The magnitude and cost of global blindness: an increasing problem that can be alleviated. *Am J Ophthalmol*. 2003;135(4):471-476. doi:10.1016/s0002-9394(02)02110-4

34. Christ SL, Lee DJ, Lam BL, Zheng DD, Arheart KL. Assessment of the effect of visual impairment on mortality through multiple health pathways: structural equation modeling. *Invest Ophthalmol Vis Sci*. 2008;49(8):3318-3323. doi:10.1167/iovs.08-1676

For peer review only

For peer review only - http://bmjopen.bmj.com/site/about/guidelines.xhtml

35. United States, Central Intelligence Agency, United States, Central Intelligence Agency. *The World Factbook 2009 (CIA's 2008 Edition)*. Potomac Books; 2008.

36. Palmer EA, Flynn JT, Hardy RJ, et al. Incidence and early course of retinopathy of prematurity. The Cryotherapy for Retinopathy of Prematurity Cooperative Group. *Ophthalmology*. 1991;98(11):1628-1640. doi:10.1016/s0161-6420(91)32074-8

37. Good WV, Hardy RJ, Dobson V, et al. The incidence and course of retinopathy of prematurity: findings from the early treatment for retinopathy of prematurity study. *Pediatrics*. 2005;116(1):15-23. doi:10.1542/peds.2004-1413

38. Cryotherapy for Retinopathy of Prematurity Cooperative Group. Multicenter Trial of Cryotherapy for Retinopathy of Prematurity: ophthalmological outcomes at 10 years. *Arch Ophthalmol*. 2001;119(8):1110-1118. doi:10.1001/archopht.119.8.1110

39. Resnikoff S, Pascolini D, Etya'ale D, et al. Global data on visual impairment in the year 2002. *Bull World Health Organ*. 2004;82(11):844-851. doi:/S0042-96862004001100009

40. Watts RD, Li IW. Use of Checklists in Reviews of Health Economic Evaluations, 2010 to 2018. *Value Health*. 2019;22(3):377-382. doi:10.1016/j.jval.2018.10.006

41. Husereau D, Drummond M, Petrou S, et al. Consolidated Health Economic Evaluation Reporting Standards (CHEERS) statement. *Value Health*. 2013;16(2):e1-5. doi:10.1016/j.jval.2013.02.010

42. Le JT, Qureshi R, Twose C, et al. Evaluation of Systematic Reviews of Interventions for Retina and Vitreous Conditions. *JAMA Ophthalmol*. 2019;137(12):1399-1405. doi:10.1001/jamaophthalmol.2019.4016

43. Mandrik OL, Severens JLH, Bardach A, et al. Critical Appraisal of Systematic Reviews With Costs and Cost-Effectiveness Outcomes: An ISPOR Good Practices Task Force Report. *Value Health*. 2021;24(4):463-472. doi:10.1016/j.jval.2021.01.002

44. Blencowe H, Lawn JE, Vazquez T, Fielder A, Gilbert C. Preterm-associated visual impairment and estimates of retinopathy of prematurity at regional and global levels for 2010. *Pediatr Res*. 2013;74(Suppl 1):35-49. doi:10.1038/pr.2013.205

45. Zupancic JAF, Ying GS, de Alba Campomanes A, Tomlinson LA, Binenbaum G, G-ROP Study Group. Evaluation of the economic impact of modified screening criteria for retinopathy of prematurity from the Postnatal Growth and ROP (G-ROP) study. *J Perinatol*. 2020;40(7):1100-1108. doi:10.1038/s41372-020-0605-5

46. Aly H, Othman HF, Munster C, Das A, Sears J. The US National Trend for Retinopathy of Prematurity. *Am J Perinatol*. Published online February 16, 2021. doi:10.1055/s-0041-1723830

47. Holmström G, Hellström A, Gränse L, et al. New modifications of Swedish ROP guidelines based on 10-year data from the SWEDROP register. *Br J Ophthalmol*. 2020;104(7):943-949. doi:10.1136/bjophthalmol-2019-314874

48. Kovács G, Somogyvári Z, Maka E, Nagyjánosi L. Bedside ROP screening and telemedicine interpretation integrated to a neonatal transport system: Economic aspects and return on investment analysis. *Early Hum Dev*. 2017;106-107:1-5. doi:10.1016/j.earlhumdev.2017.01.007

49. Agarwal K, Jalali S. Classification of retinopathy of prematurity: from then till now. *Community Eye Health*. 2018;31(101):S4-S7.

50. Nguyen QD, Tawansy K, Hirose T. Recent advances in retinopathy of prematurity. *Int Ophthalmol Clin*. 2001;41(4):129-151. doi:10.1097/00004397-200110000-00013

For peer review only - http://bmjopen.bmj.com/site/about/guidelines.xhtml

51. Lundgren P, Jacobson L, Hård AL, et al. High rate and large intercentre variability in retreatment of retinopathy of prematurity in infants born <24 gestational weeks. *BMJ Open Ophthalmol*. 2021;6(1):e000695. doi:10.1136/bmjophth-2020-000695

52. Shah PK, Prabhu V, Karandikar SS, Ranjan R, Narendran V, Kalpana N. Retinopathy of prematurity: Past, present and future. *World J Clin Pediatr*. 2016;5(1):35-46. doi:10.5409/wjcp.v5.i1.35

53. Vision impairment and blindness. World Health Organization. Accessed August 26, 2021. https://www.who.int/news-room/fact-sheets/detail/blindness-and-visual-impairment

54. Naguib MM, Soares RR, Anzures R, et al. Regionally Specific Economic Impact of Screening and Treating Retinopathy of Prematurity in Middle-Income Societies in the Philippines. *J Pediatr Ophthalmol Strabismus*. 2019;56(6):388-396. doi:10.3928/01913913-20190925-02

55. Köberlein J, Beifus K, Schaffert C, Finger RP. The economic burden of visual impairment and blindness: a systematic review. *BMJ Open*. 2013;3(11):e003471. doi:10.1136/bmjopen-2013-003471

56. Chakravarthy U, Biundo E, Saka RO, Fasser C, Bourne R, Little JA. The Economic Impact of Blindness in Europe. *Ophthalmic Epidemiol*. 2017;24(4):239-247. doi:10.1080/09286586.2017.1281426

57. Humayun J, Löfqvist C, Ley D, Hellström A, Gyllensten H. Systematic review of the healthcare cost of bronchopulmonary dysplasia. *BMJ Open*. 2021;11(8):e045729. doi:10.1136/bmjopen-2020-045729

For peer review only

For peer review only - http://bmjopen.bmj.com/site/about/guidelines.xhtml

Tables

Table 1. Overview of Studies Included in This Review.

#	First author (year)	Country (study period) Setting	Study design	ROP definition	Sample size (% of infants with ROP treated)	Inclusion criteria	Mean cost per child with ROP (value year and currency as reported in the original publication)	Cost perspective: cost inclusion
1	Mohammadi (2021) [14]	Iran (2017) Data from Farabi eye hospital	Decision Analytical Model from case series	Threshold ROP	Total: 126 ROP: 126	Randomly selected infants with treatment requiring ROP	**Treatment**: US$1107/infant	Unclear perspective: out-of-pocket charges[a]

For peer review only

2	Moitry (2018) [15]	France (2012 and 2014-2015) Data from two hospitals CHSF and Port-Royal	Retrospective, before-and-after study	Type 1 ROP	Not specified	GA<33 w or BW<1500 g	**Screening**: €37/exam	Health system: direct costs
3	Isaac (2018) [16]	Canada (2009–2014) Data from Ontario Ministry of Health and Long-Term Care	Retrospective cohort study (chart review)	Type 1 ROP	Total: 174 ROP: 64 Treated: 3 (5.6%)	BW<1500 g or GA<30 w	**Screening HSN**: C$346/exam (SD: C$306) **Screening RVH**: C$375/exam (SD: C$300)	Health system: direct costs (excluding equipment and maintenance)
4	Kelkar (2017a) [17]	India (2009–2011) Mobile ROP screening unit	Public health intervention[b] from case series	ICROP guidelines	Total: 104 ROP: 34 Treated: 5 (15%)	BW<1700 g or GA<34 w	**Screening**: US$240/exam[c]	Health system: direct healthcare costs

For peer review only - http://bmjopen.bmj.com/site/about/guidelines.xhtml

							**Identifying an infant with ROP:** US$735/infant[c] **Treatment**: US$6500/infant	(including salaries and equipment)
5	Kelkar (2017b) [18]	India (2013–2015) Data from 5 NICUs	Public health intervention[b] from case series	ICROP guidelines	Total: 102 ROP: 32 Treated: 4 (15%)	BW<1700 g or GA<34 w	**Screening**: US$199/infant[d] **Identifying an infant with ROP**: US$596/infant[d] **Treatment**: US$4137/infant	Health system: direct costs (including salaries and equipment)
6	Rothschild (2016) [19]	Mexico and US (2014) Data from pediatric eye	Decision Analytical Model from case series	ROP caused blindness (WHO)	Total: 95	BW<1500 g	**US screening**: US$981/infant **Mexico screening:** US$333/infant	Third party payer: charges (including

For peer review only - http://bmjopen.bmj.com/site/about/guidelines.xhtml

		clinics and schools for the blind in Atlanta, Georgia, and Mexico City Blindness costs from the literature[33] and other secondary sources.					**US treatment**: US$4037/infant **Mexico treatment:** US$505/infant **US follow-up**: US$1538/infant **Mexico follow-up**: US$2214/infant **US blindness cost**: US$84586/infant **Mexico blindness cost:** US$24413/infant	labor and equipment) Societal costs: expenses for raising a blind child
7	van der Akker-van Merle	Netherlands (2009) Data from NEDROP study	Retrospective cohort study	ICROP guidelines	Total: 1380 ROP: 29 Treated: 17 (59%)	GA<32 w or BW<1500 g	**Screening**: €109/exam **Treatment**: €2755/infant	Health system: direct costs

For peer review only - http://bmjopen.bmj.com/site/about/guidelines.xhtml

For peer review only

	(2015) [20]	and PRN database						
8	Wongwai (2015) [21]	Thailand (2013) Hypothetical data and cohort Blindness costs using secondary data on annual government subsidies and utilities from the literature[34]	Decision Analytical Model from prospective cohort study	ET-ROP criteria	Total: 100 ROP: 9		**Screening**: THB 142/infant **Treatment**: THB (SE) 1053 (316)/infant **Lifetime cost of blindness**: THB 146,000 **Telemedicine screening**: THB 17,397/QALY (3% disc. rate)	Third party payer: charges (including labor and equipment)
9	Black (2015) [22]	US (2001–2010) Medical University of South Carolina	Retrospective cohort study	ROP stage 4	Total: 4292 ROP: 7 Treated: 7 (100%)	GA: 23–37 w	**Cost increase due to ROP if:** **GA (23 w):** US$19,513	Hospital: direct costs

For peer review only - http://bmjopen.bmj.com/site/about/guidelines.xhtml

							**GA (mean, 34.3 w):** US$23,121 **GA (37 w):** US$41,161	
10	Zin (2014) [23]	Brazil (2004–2006) 6 NICUs in Rio de Janeiro	Decision Analytical Model from case series and expert opinion	ICROP criteria	Total: 869 ROP: 70 Treated: 70 (100%)	BW<1500 g	**Screening**: US$18/infant **Treatment**: US$398/infant	Health system: direct costs (including labor and equipment)
11	Dave (2012) [24]	Peru (2009) Data from local hospital's NICU and from 2002 study[39]	Retrospective cohort study	ROP stage 1–5 with/without plus disease	Total: 1239 ROP: 80		**Screening and treatment**: US$2496/infant **Follow-up** (3 visits): US$54 **ROP caused blindness:** US$123,806/infant	Health system: direct costs (including equipment, facility, labor and supplies)

For peer review only - http://bmjopen.bmj.com/site/about/guidelines.xhtml

		Secondary source for blindness costs[35]						Societal costs: expenses for blindness
12	Dunbar (2009)[25]	US (2004–2006) Medicare and Medicaid reimbursement data from California and Louisiana	Microsimulation model from retrospective cohort study	Type 1 ROP	Total: 515<br>ROP: 58<br>Treated: 58 (100%)	BW<1500 g or GA<28 w	**Screening**: US$93/exam<br>**Screening**: US$316/infant<br>**Treatment w/o anesthesia**: US$1371/infant<br>**Screening and treatment:** US$1565/QALY (3% disc. rate)	Third-party payer (Medicare and Medicaid): charges (excluding anesthesia)

For peer review only - http://bmjopen.bmj.com/site/about/guidelines.xhtml

13	Kamholz (2009) [26]	US (2005) Data from ET-ROP study	Decision Analytical Model from randomized trial and expert opinion	Type 1 ROP	ROP: 357	BW<1250 g or GA<32 w	**Screening**: US$189/exam (US$56–$251); **treatment w/o anesthesia**: US$2423 (US$638–$3223) **Anesthesia**: US$1849 (US$925–$3698)	Third-party payer: charges
14	Jackson (2008) [27]	US (2006) Data from CRYO-ROP and ET-ROP studies	Decision Analytical Model from randomized trial	Type 1 ROP	Refer to published data on 4099 infants (65.8% with ROP[36]) and 6998 infants (68% with ROP[37])	BW<1251g	**Screening**: US$160/exam **Screening and treatment**: US$4410/QALY (3% disc. rate.)	Third-party payer (Medicare): charges

For peer review only - http://bmjopen.bmj.com/site/about/guidelines.xhtml

15	Yanowitch (2006) [28]	US (2001–2004) Data from Dean A. McGee Eye Institute and OUHSC campus	Retrospective cohort study (chart review)	CRYO-ROP and ET-ROP criteria	Total: 259 ROP: 11 Treated: 1 (9%)	BW 1250–1800 g	**Screening**: US$230/infant **Treatment**: US$2000/infant	Third-party payer: charges
16	Castillo-Riquelme (2004) [29]	UK (1997-1998) Data from published data[38] and local NICU	Decision Analytical Model from case series and expert opinion	ROP stage 3	ROP: 235	GA<32 or BW<1501 g	**Screening**: £49/exam **Screening:** £279/infant **Treatment**: £540/one eye **Treatment:** £702/two eyes **Follow-up** (10 years): £786/infant	Health system: direct costs (including equipment and maintenance)
17	Lee (2001) [30]	Canada (1996-1997)	Retrospective cohort study	Threshold ROP	Total: 16,424	Different criteria at	**Screening**: C$236/infant **Treatment**: C$2655/infant	Health system: direct costs

For peer review only - http://bmjopen.bmj.com/site/about/guidelines.xhtml

		Data from 14 NICU				different NICU		
18	Brown (1999) [31]	US (1998) Database from Pennsylvania	Microsimulation model from randomized trial	Threshold ROP	ROP: 291 Treated: 291 (100%) but only one treated eye per infant	BW<1251 g	**Treatment**: US$1452/infant **Treatment consultation**: US$140/exam **Treatment**: US$678/QALY (3% disc. rate)	Third-party payer: charges
19	Javitt (1993) [32]	US (1989) Medicare reimbursement data	Microsimulation model from retrospective cohort study	Threshold ROP or PNA 24 weeks from CRYO-ROP	Total: 18,220 ROP: 1000 Treated: 1000 (100%)	BW: 500–1249 g	**Screening (1st visit)**: US$84/exam **Screening (subsequent visit)**: US$68/exam **Screening (weekly)**: US$6045/QALY	Third-party payer: charges (excluding equipment and personnel training cost)

For peer review only - http://bmjopen.bmj.com/site/about/guidelines.xhtml

							**Screening (biweekly)**: US$3623/QALY **Screening (monthly)**: US$2488/QALY	

[a] Assumption based on methods description indicating cost data collected through survey to parents.

[b] Studies of the introduction of new screening programs.

[c] Screening costs and costs for identifying an infant with ROP are reduced by 22.6% to account for transport costs (i.e., driver and cost of van and fuel to move equipment).

[d] Screening costs and costs for identifying an infant with ROP are reduced by 0.245% to account for transport costs (i.e., fuel to move equipment).

Abbreviations: BW=birth weight; disc.=discount; GA=gestational age; HSN=Health Sciences North in Sudbury, Canada; NICU=neonatal intensive care unit; PNA=postnatal age; QALY=quality-adjusted life years; ROP=retinopathy of prematurity; RVH=Royal Victoria Hospital in Barrie, Canada; US=United States of America; WHO=World Health Organization

For peer review only - http://bmjopen.bmj.com/site/about/guidelines.xhtml

Table 2. Costs for Screening for ROP Among Preterm Infants (in 2020 values)

#	First author (year)	Screening costs		Treatment costs	Evidence rating	Cost inclusion
		Mean per exam (US$)	Mean per infant (US$)	Mean per infant (US$)		
1	Mohammadi (2021) [14]	-	-	1169	4	Charges
2	Moitry (2018) [15]	44	-	-	3	Direct cost
3	Isaac (2018) [16]	HSN: 342<br>RVH: 371	-	-	3	Direct cost **not including equipment**
4	Kelkar (2017a) [17]	253	-	6500	4	Direct cost including equipment and labor
5	Kelkar (2017b) [18]	210	-	4137	4	Direct cost including equipment and labor
6	Rothschild (2016) [19]		US: 1072<br>Mexico: 362	US: 4413<br>Mexico: 552	4	Direct cost including equipment and labor

For peer review only - http://bmjopen.bmj.com/site/about/guidelines.xhtml

7	van der Akker-van Merle (2015) [20]	160	-	4064[a]	3	Direct cost
8	Wongwai (2015) [21]	5	-	38	2	Charges including equipment and labor
9	Black (2015) [22]	-	-	-	3	-
10	Zin (2014) [23]	20	-	450	5	Direct cost including equipment and labor
11	Dave (2012) [24]	-	-	-	3	-
12	Dunbar (2009) [25]	119	405	1759	3	Charges
13	Kamholz (2009) [26]	250	-	5661[a]	5	Charges
14	Jackson (2008) [27]	205	-	-	1	Charges
15	Yanowitch (2006) [28]	-	324	2814	3	Charges
16	Castillo-Riquelme (2004) [29]	106	602	Unilateral: 1165	5	Direct cost including

For peer review only - http://bmjopen.bmj.com/site/about/guidelines.xhtml

				Bilateral: 1514		equipment and maintenance
17	Lee (2001) [30]	Unilateral: 112	-	2507	3	Direct cost
18	Brown (1999) [31]	-	-	2527	1	Charges
19	Javitt (1993) [32]	First: 183 Follow-up: 149	-	-	3	Charges

Evidence rating indicates the quality of evidence rating of included studies: 1=e.g., properly powered randomized controlled trials; 2=e.g., prospective cohort studies; 3=e.g., retrospective cohort studies; 4=case series with or without intervention or cross-sectional study; 5=e.g., opinion of respected authorities.

[a] Unit cost per treatment.

Abbreviations: HSN=Health Sciences North in Sudbury, Canada; ROP=retinopathy of prematurity; RVH=Royal Victoria Hospital in Barrie, Canada; US=United States of America

For peer review only

Figure Titles and Legends

Figure 1. Map of data availability and costs per a) screening visit and b) treatment. The map illustrates reported costs or means of reported costs per country for included studies in US$. In studies presenting only total screening cost per infant or by first/follow-up visits,[19,28,32] the cost level per screening was calculated under the assumption of four screening visits per infant. Where only screening cost per eye was reported,[30] it was duplicated to obtain the cost level per screening. In studies reporting only unit cost per treatment,[20,26] the unit cost was assumed to indicate the cost level of treatment per infant. Where costs were reported separately for unilateral and bilateral treatment,[29] a weighted mean cost was calculated assuming that 75% of treatments were bilateral.

Figure 2. Forest plot of treatment costs, by country categorization. In parentheses, ER of included studies: 1=e.g., properly powered randomized controlled trials; 2=e.g., prospective cohort studies; 3=e.g., retrospective cohort studies; 4=case series with or without intervention or cross-sectional study; 5=e.g., opinion of respected authorities. Abbreviations: CI=confidence interval; ER=Evidence rating; REML=Restricted Maximum Likelihood. Country abbreviated according to ISO code.

Figure 3. Forest plot of treatment costs, cumulative results by year, and country categorization. In parentheses, ER of included studies: 1=e.g., properly powered randomized controlled trials; 2=e.g., prospective cohort studies; 3=e.g., retrospective cohort studies; 4=case series with or without intervention or cross-sectional study; 5=e.g., opinion of respected authorities. Abbreviations: CI=confidence interval; ER=Evidence rating; REML=Restricted Maximum Likelihood. Country abbreviated according to ISO code.

For peer review only - http://bmjopen.bmj.com/site/about/guidelines.xhtml

For peer review only

Figure 1a) World map - cost per screening

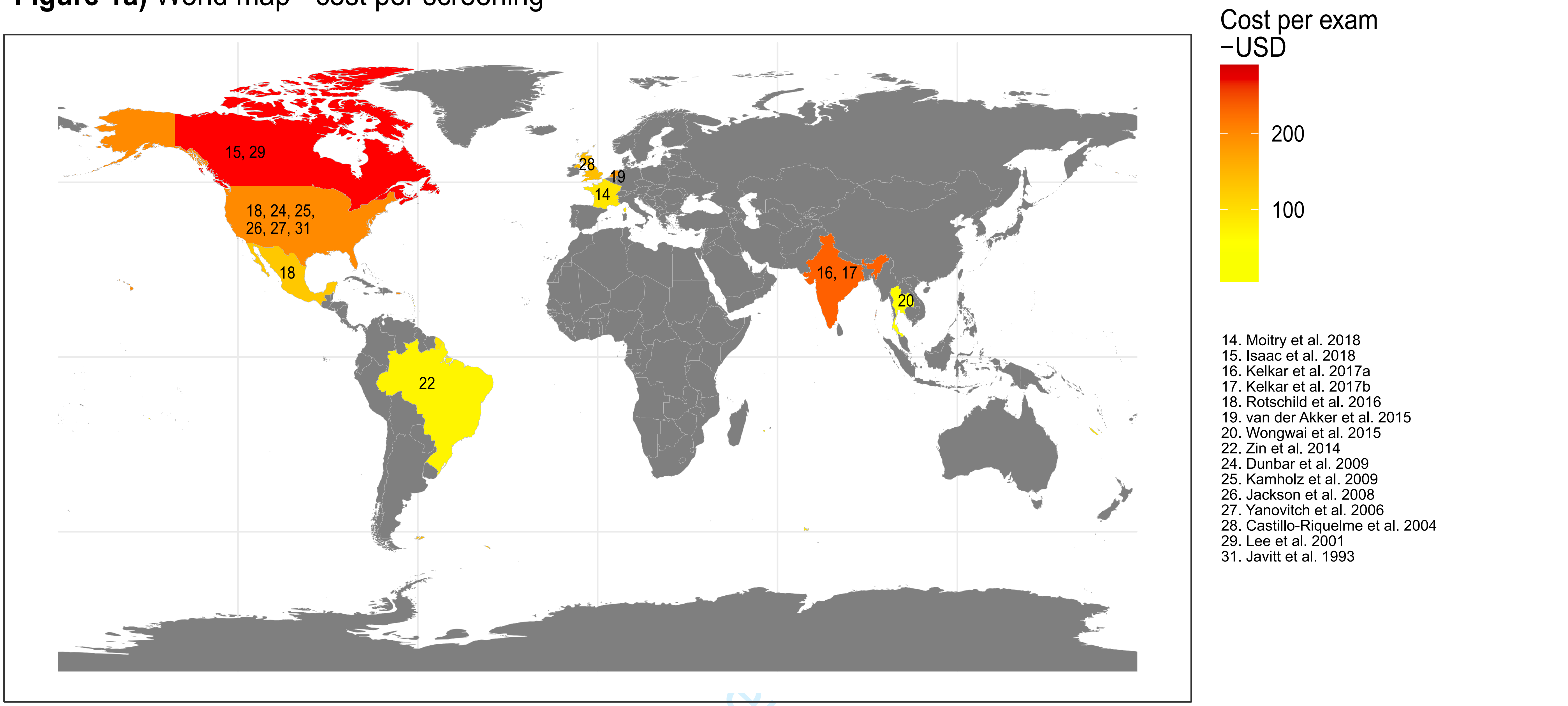

1b) World map −cost per treatment

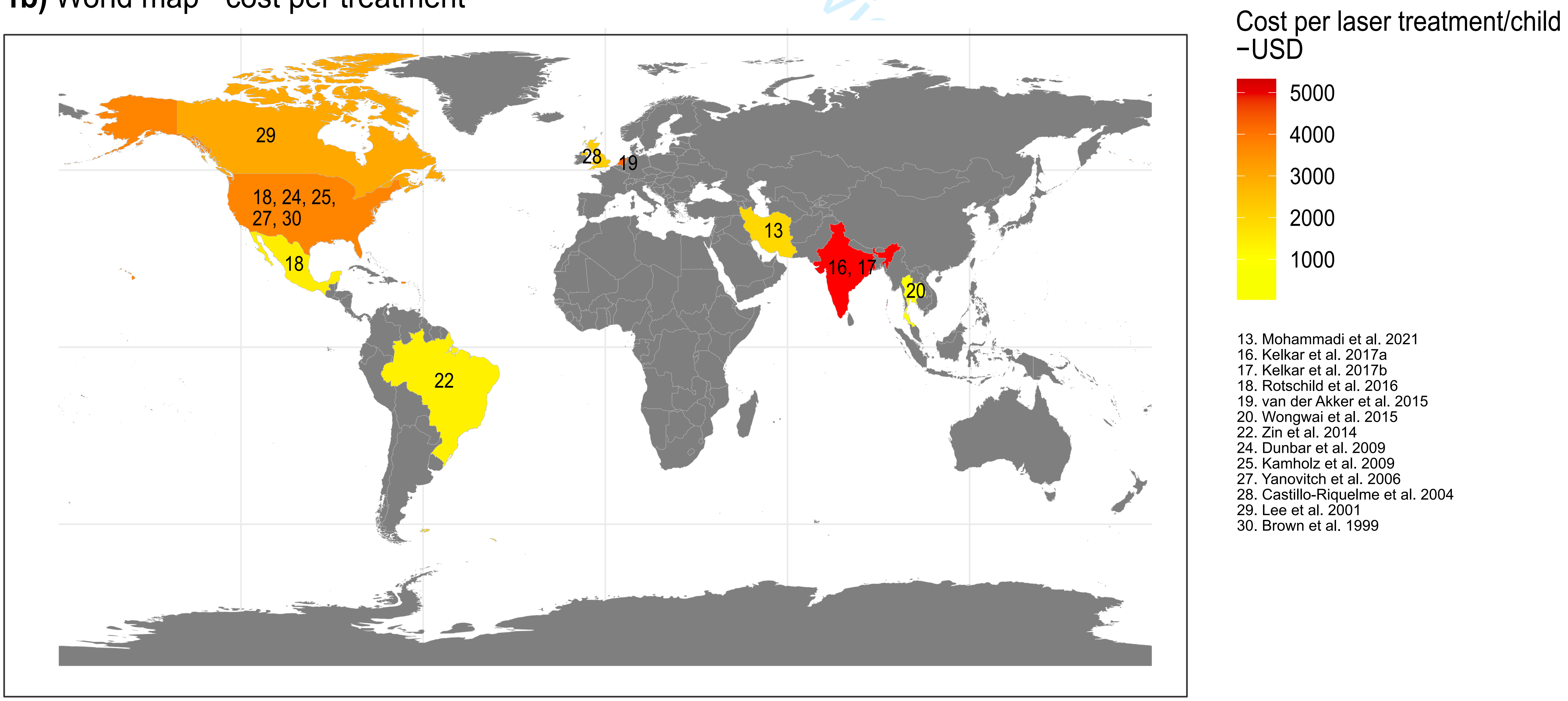

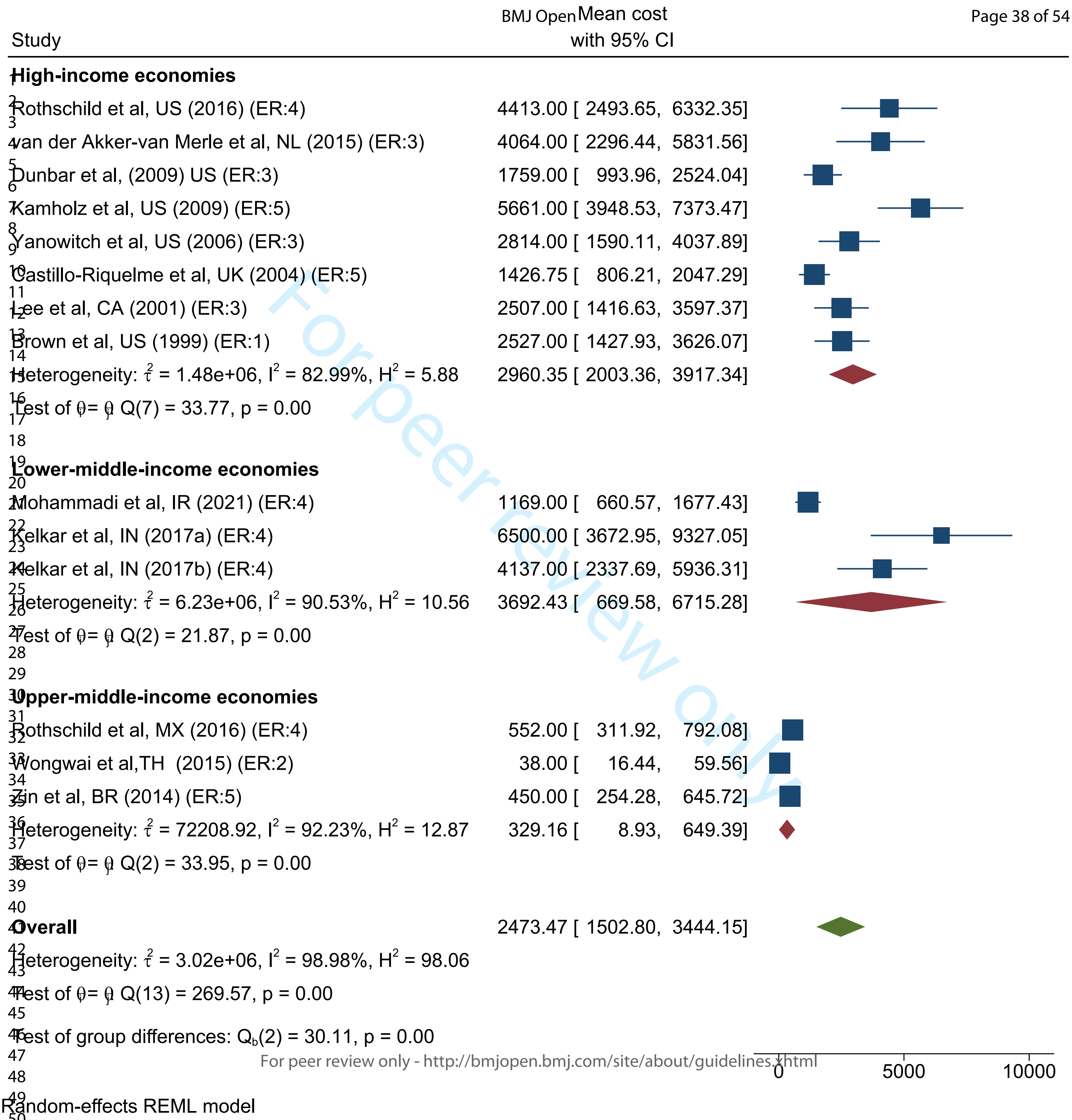
Study
Mean cost with 95% CI
High-income economies
Rothschild et al, US (2016) (ER:4) 4413.00 [2493.65, 6332.35]
van der Akker-van Merle et al, NL (2015) (ER:3) 4064.00 [2296.44, 5831.56]
Dunbar et al, (2009) US (ER:3) 1759.00 [993.96, 2524.04]
Kamholz et al, US (2009) (ER:5) 5661.00 [3948.53, 7373.47]
Yanowitch et al, US (2006) (ER:3) 2814.00 [1590.11, 4037.89]
Castillo-Riquelme et al, UK (2004) (ER:5) 1426.75 [806.21, 2047.29]
Lee et al, CA (2001) (ER:3) 2507.00 [1416.63, 3597.37]
Brown et al, US (1999) (ER:1) 2527.00 [1427.93, 3626.07]
Heterogeneity: τ^2 = 1.48e+06, I^2 = 82.99%, H^2 = 5.88 2960.35 [2003.36, 3917.34]
Test of $\theta_i = \theta_j$: Q(7) = 33.77, p = 0.00
Lower-middle-income economies
Mohammadi et al, IR (2021) (ER:4) 1169.00 [660.57, 1677.43]
Kelkar et al, IN (2017a) (ER:4) 6500.00 [3672.95, 9327.05]
Kelkar et al, IN (2017b) (ER:4) 4137.00 [2337.69, 5936.31]
Heterogeneity: τ^2 = 6.23e+06, I^2 = 90.53%, H^2 = 10.56 3692.43 [669.58, 6715.28]
Test of $\theta_i = \theta_j$: Q(2) = 21.87, p = 0.00
Upper-middle-income economies
Rothschild et al, MX (2016) (ER:4) 552.00 [311.92, 792.08]
Wongwai et al,TH (2015) (ER:2) 38.00 [16.44, 59.56]
Zin et al, BR (2014) (ER:5) 450.00 [254.28, 645.72]
Heterogeneity: τ^2 = 72208.92, I^2 = 92.23%, H^2 = 12.87 329.16 [8.93, 649.39]
Test of $\theta_i = \theta_j$: Q(2) = 33.95, p = 0.00
Overall 2473.47 [1502.80, 3444.15]
Heterogeneity: τ^2 = 3.02e+06, I^2 = 98.98%, H^2 = 98.06
Test of $\theta_i = \theta_j$: Q(13) = 269.57, p = 0.00
Test of group differences: $Q_b(2)$ = 30.11, p = 0.00
0
5000
10000
Random-effects REML model

Study	Mean cost with 95% CI
**High-income economies**	
Brown et al, US (1999) (ER:1)	2527.00 [1427.93, 3626.07]
Lee et al, CA (2001) (ER:3)	2516.92 [1742.86, 3290.99]
Castillo-Riquelme et al, UK (2004) (ER:5)	2039.56 [1240.70, 2838.41]
Yanowitch et al, US (2006) (ER:3)	2193.88 [1478.16, 2909.59]
Dunbar et al, US (2009) (ER:3)	2056.83 [1508.58, 2605.09]
Kamholz et al, US (2009) (ER:5)	2634.39 [1579.11, 3689.66]
van der Akker-van Merle et al, NL (2015) (ER:3)	2798.19 [1805.71, 3790.68]
Rothschild et al, US (2016) (ER:4)	2960.35 [2003.36, 3917.34]
**Lower-middle-income economies**	
Kelkar et al, IN (2017a) (ER:4)	6500.00 [3672.95, 9327.05]
Kelkar et al, IN (2017b) (ER:4)	5056.60 [2798.51, 7314.69]
Mohammadi et al, IR (2021) (ER:4)	3692.43 [669.58, 6715.28]
**Upper-middle-income economies**	
Zin et al, BR (2014) (ER:5)	450.00 [254.28, 645.72]
Wongwai et al,TH (2015) (ER:2)	232.05 [-171.03, 635.12]
Rothschild et al, MX (2016) (ER:4)	329.16 [8.93, 649.39]

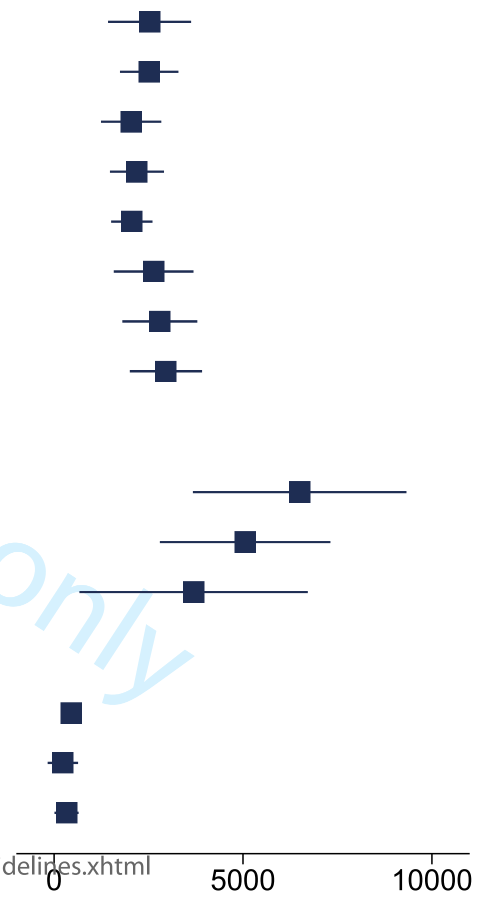

Random-effects REML model

For peer review only

Online-Only Supplements

Costs associated with Retinopathy of prematurity: A Systematic Review and Meta-analysis

Authors

Hanna Gyllensten[1,2], Associate professor

Jhangir Humayun[1,2], B.Sc.

Ulrika Sjöbom[1,3], M.Sc.

Ann Hellström[3], Professor

Chatarina Löfqvist[1,2,3], Associate professor

Author affiliations

[1] Institute of Health and Care Sciences, Sahlgrenska Academy, University of Gothenburg, Box 457, SE-405 30 Gothenburg, Sweden.

[2] Centre for Person-Centred Care (GPCC), University of Gothenburg, Box 457, SE-405 30 Gothenburg, Sweden.

[3] Department of Clinical Neuroscience, Institute of Neuroscience and Physiology, Sahlgrenska Academy, University of Gothenburg, Gothenburg, Sweden.

Content

Authors 1
  Author affiliations 1
eTable 1. Search terms 2
eTable 2. Data extraction sheet 2
  Data extraction 2
  Quality assessment (according to instrument developed by Evers et al[1]) 2
eTable 3. Checklist for the quality appraisal of included papers (from Evers et al[1]) 2
eFigure 1. Flow diagram shows the study selection process, following the PRISMA guidelines.[21] 4
eTable 4. Excluded articles* 5
eFigure 2. Cost model 6
  Preterm birth 7
  ROP screening 7
  Lifetime (treatment and follow-up) 7
References 8

For peer review only

eTable 1. Search strategy[a]

Database	Search string
Pubmed	(((((((Retinopathy) AND Prematur*) OR ((Terry) AND Syndrom*) OR ("ROP"[Title/Abstract]) OR "Retinopathy of Prematurity"[Mesh])) AND (("Economics"[Mesh]) OR ((economic*[Title/Abstract] OR cost[Title/Abstract] OR costs[Title/Abstract] OR costly[Title/Abstract] OR costing[Title/Abstract] OR price[Title/Abstract] OR prices[Title/Abstract] OR pricing[Title/Abstract] OR pharmacoeconomic*[Title/Abstract]))))))
Scopus	(TITLE-ABS-KEY ("Retinopath*") AND TITLE-ABS-KEY ("Prematur*")) OR (TITLE-ABS-KEY ("Retrolental") AND TITLE-ABS-KEY ("Fibroplas*")) OR (TITLE-ABS-KEY ("Terry") AND TITLE-ABS-KEY ("Syndrom*")) AND (TITLE-ABS-KEY (economic* OR cost OR cos OR costly OR costing OR price OR prices OR pricing OR pharmacoeconomic*))

[a] No filters or limitations were used in the searches of databases.

eTable 2. Data extraction sheet

Data extraction

- Reviewer
- Reference (APA)
- Aim/Objective
- Study design
- When was it conducted
- Setting including country and hospital name/database
- How is ROP severity defined
- Total study participants
- Patients with ROP (N)
- Patient group description
- Controls (N)
- Control group description
- Average cost of screening (total per infant/per visit/per eye)
- What costs are measured
- How are the costs measured
- Average Cost for infants with diagnosed sight-threatening ROP
- What costs are measured
- How are the costs measured
- Costs from which year (if adjusted, which year)
- Perspective: cost analysis
- Time horizon of cost analysis
- Funding
- Limitations: Confounders and biases reported
- Conclusions (by author)

Quality assessment (according to instrument developed by Evers et al[1])

1. Is the study population clearly described?
2. Are competing alternatives clearly described?
3. Is a well-defined research question posed in answerable form?
4. Is the economic study design appropriate to the stated objective?
5. Is the chosen time horizon appropriate in order to include relevant costs and consequences?
6. Is the actual perspective chosen appropriate?
7. Are all important and relevant costs for each alternative identified?
8. Are all costs measured appropriately in physical units?
9. Are costs valued appropriately?
10. Are all important and relevant outcomes for each alternative identified?
11. Are all outcomes measured appropriately?
12. Are outcomes valued appropriately?
13. Is an incremental analysis of costs and outcomes of alternatives performed?
14. Are all future costs and outcomes discounted appropriately?
15. Are all important variables, whose values are uncertain, appropriately subjected to sensitivity analysis?
16. Do the conclusions follow from the data reported?
17. Does the study discuss the generalizability of the results to other settings and patient/client groups?
18. Does the article indicate that there is no potential conflict of interest of study researcher(s) and funder(s)?
19. Are ethical and distributional issues discussed appropriately

For peer review only

eTable 3. Checklist for the quality appraisal of included papers (from Evers et al[1])

First authors / Checklist items[a]	Black[2]	Brown[3]	Castillo-Requilme[4]; Javitt[5]; Lee[6]; Rothchild[7]; Wongwai[8]	Dave[9]	Dunbar[10]	Isaac[11]	Kamholz[12]; Jackson[13]	Kelkar (2017a)[14]; Kelkar (2017b)[15]	Mohammadi[16]	Moitry[17]	Van den Akker-van Merle[18]	Yanowitch[19]	Zin[20]	**Total**
1	+	+	+	+	+	+	-	+	-	+	+	+	+	**16**
2	+	+	+	+	+	+	+	+	+	+	+	+	+	**19**
3	+	+	+	+	+	+	+	+	+	+	+	+	+	**19**
4	+	+	+	+	+	+	+	+	+	+	+	+	+	**19**
5	+	+	+	+	+	+	+	+	+	+	+	+	+	**19**
6	+	+	+	+	+	+	+	+	-	+	+	+	+	**18**
7	+	+	+	+	+	+	+	+	+	+	+	+	+	**19**
8	+	+	+	+	+	+	+	+	+	+	+	+	+	**19**
9	+	+	+	+	+	+	+	+	+	+	+	+	+	**19**
10	+	+	+	+	+	+	+	+	+	+	+	+	+	**18**
11	+	+	+	+	+	+	+	+	+	+	+	+	+	**19**
12	+	+	+	+	+	+	+	+	+	+	+	+	+	**19**
13	+	-	+	+	+	+	+	-	-	-	+	+	+	**14**
14	-	-	+	-	+	-	+	-	-	+	+	+	-	**11**
15	+	-	+	-	-	-	+	-	-	+	-	-	+	**10**
16	+	+	+	+	+	+	+	+	+	+	+	+	+	**19**
17	+	+	+	+	+	+	+	+	+	+	+	+	+	**19**
18	+	+	+	+	-	+	+	-	+	+	-	+	+	**15**

For peer review only

19	+	+	+	+	+	+	+	-	+	+	+	+	+	**17**
**Total**	**18**	**16**	**19**	**17**	**17**	**17**	**18**	**14**	**14**	**18**	**17**	**17**	**18**	

[a] Item numbering (also in eTable 2): 1. Is the study population clearly described?; 2. Are competing alternatives clearly described?; 3. Is a well-defined research question posed in answerable form?; 4. Is the economic study design appropriate to the stated objective?; 5. Is the chosen time horizon appropriate in order to include relevant costs and consequences?; 6. Is the actual perspective chosen appropriate?; 7. Are all important and relevant costs for each alternative identified?; 8. Are all costs measured appropriately in physical units?; 9. Are costs valued appropriately?; 10. Are all important and relevant outcomes for each alternative identified?; 11. Are all outcomes measured appropriately?; 12. Are outcomes valued appropriately?; 13. Is an incremental analysis of costs and outcomes of alternatives performed?; 14. Are all future costs and outcomes discounted appropriately?; 15. Are all important variables, whose values are uncertain, appropriately subjected to sensitivity analysis?; 16. Do the conclusions follow from the data reported?; 17. Does the study discuss the generalizability of the results to other settings and patient/client groups?; 18. Does the article indicate that there is no potential conflict of interest of study researcher(s) and funder(s)?; 19. Are ethical and distributional issues discussed appropriately?

For peer review only

eFigure 1. Flow diagram shows the study selection process, following the PRISMA guidelines.[21]

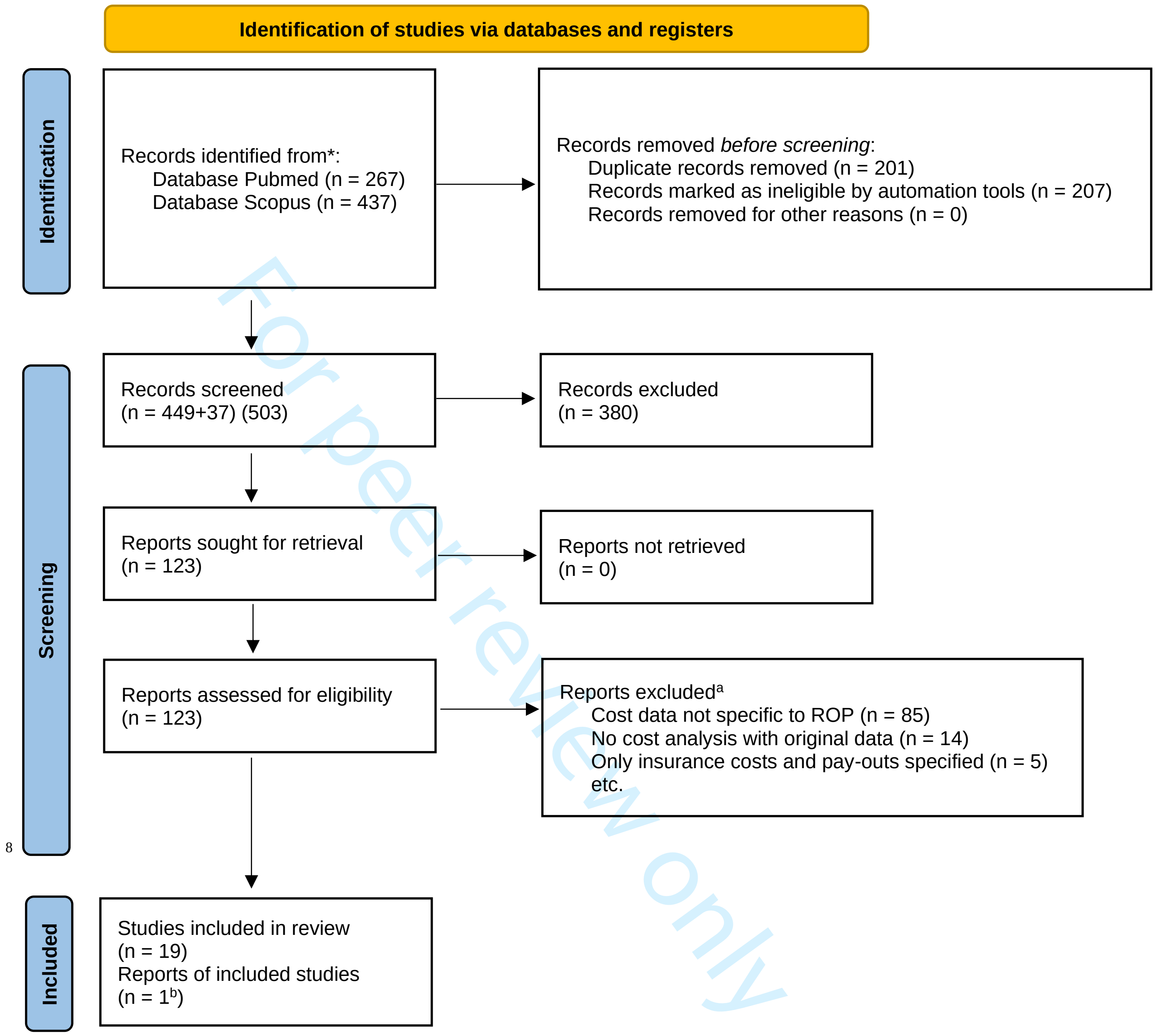

[a] For detailed reasons for exclusion of studies that might appear to meet the inclusion criteria, but which were excluded, see also eTable 4.

[b] One author[8] was contacted and clarified the currency of reported results. Another author[16] was unsuccessfully contacted to clarify cost perspective.

Abbreviations: ROP = Retinopathy of prematurity.

For peer review only

eTable 4. Excluded articles[a]

Study	Reason for exclusion
Cross 1973. Cost of preventing retrolental fibroplasia?[22]	No original cost data.
Boncz et al., 2013. [Health-economic analysis of diseases related to disturbed neonatal adaptation: A cost of illness study].[23]	Only insurance payouts.
Yo et al., 2018. Retinopathy of prematurity: the high cost of screening regional and remote infants.[24]	Transport costs but no screening costs.
Scholz and Greiner, 2019. An exclusive human milk diet for very low birth weight newborns-A cost-effectiveness and EVPI study for Germany.[25]	No ROP specific costs.
Zupancic et al., 2020. Evaluation of the economic impact of modified screening criteria for retinopathy of prematurity from the Postnatal Growth and ROP (G-ROP) study.[26]	No original cost data.

[a] In this table are listed studies that might appear to meet the inclusion criteria, but which were excluded, and why they were excluded.

Abbreviations: ROP = Retinopathy of prematurity.

For peer review only

eFigure 2. Cost model

This figure presents our preliminary suggestions for a conceptual model for costs associated with retinopathy of prematurity (ROP), with some additional comments we believe are relevant. Abbreviations: GA=gestational age; ROP=retinopathy of prematurity; VEGF=vascular endothelial growth factor.

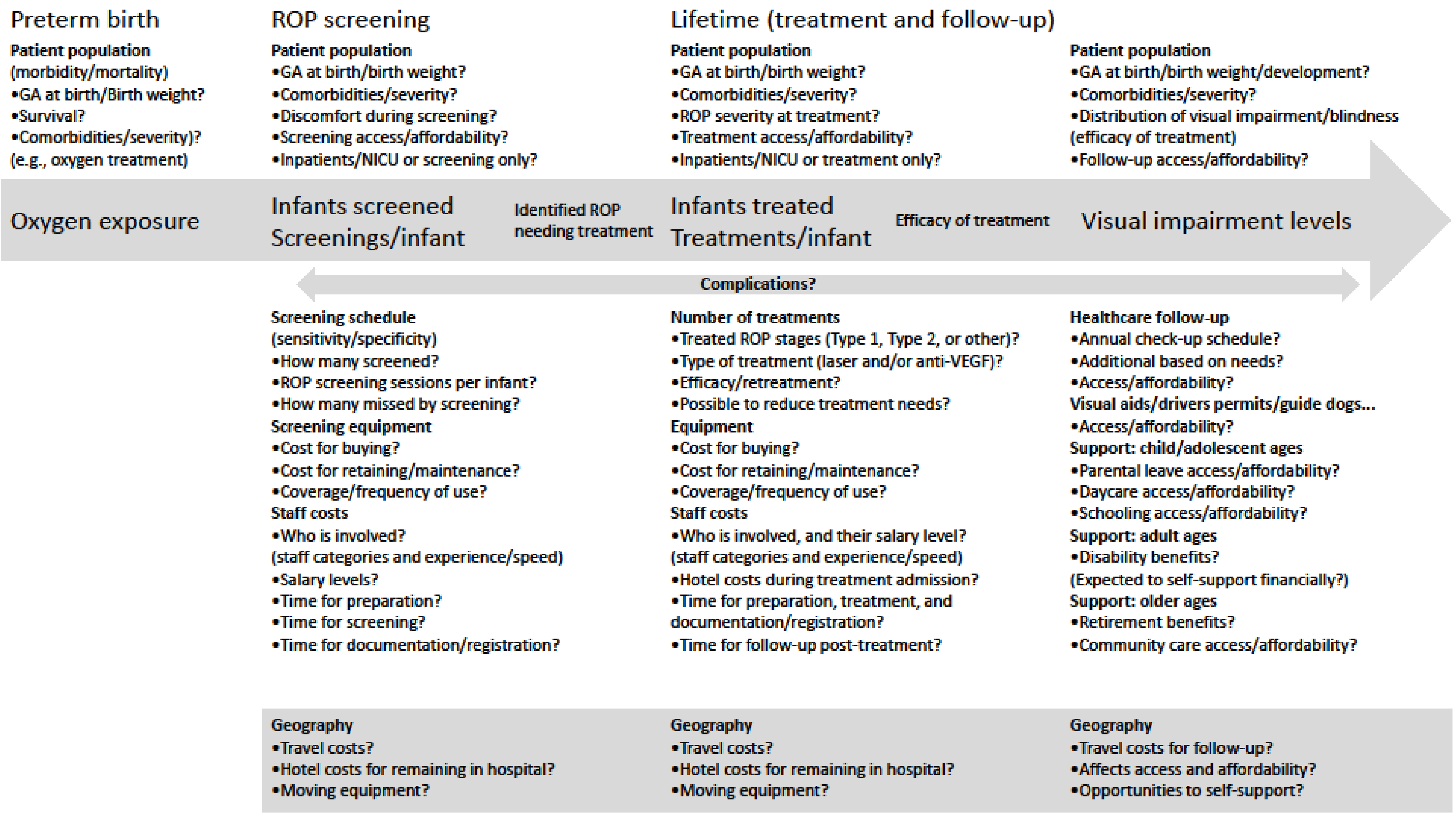

Preterm birth

It should be noted that these costs are part of a larger picture of understanding the economic impact of prematurity, which is essential knowledge in predicting the costs and consequences of introducing new interventions that affect gestational age at birth or morbidity and mortality among preterm infants. Thus, the model here is only one part and should be complemented by factors related to, e.g., bronchopulmonary dysplasia and other lung diseases, as well as other neuropsychiatric conditions. The listed items add to the previously published compartmental model of the global burden of ROP,[27] which also accounts for e.g., availability and coverage of screening programs.

ROP screening

Some evidence suggests that screening can be reduced even as infants are still identified with high sensitivity and specificity.[5] Reduced screening can be achieved through either changing the frequency of screening or limiting who is actually screened. Based on register findings in Sweden, infants born after gestational week 30 are no longer routinely screened for ROP.[28] Similarly, a study from the Netherlands found no severe ROP among infants born ≥30 gestational weeks.[29] This pattern differs from the situation in many other parts of the world. However, infants born at lower gestational age are more likely to develop ROP and severe ROP.[30]

Costs for screening in the studies included staff salaries/time, equipment and maintenance, supplies, and staff training. Although the identified studies do not detail the cost components and their associated costs, it can be expected that the reported costs of screening are to some extent underestimated. In time-and-motion studies conducted in our local hospital during a process of developing services (unpublished results), the times spent for preparatory work and documentation of screening results were 7–15 minutes and 7–12 minutes, respectively. This range included the time needed to identify infants who should be screened from those born at the facility, but excluded the time used for the actual screening. The figures can be compared to numbers provided in, e.g., Wongwai et al.,[8] citing 10 minutes used for screening by the ophthalmologist and 60 minutes for the nurse. According to Jackson et al.,[13] an average five examinations were necessary for determining if one infant would require treatment for ROP, which is in line with experiences in our hospital.

Regardless of the setting, there will also be transportation costs associated with screening. In this review, we excluded transportation costs, which are highly specific to each setting. For example, an Australian study reported flights for ROP screening to average 36–75 minutes depending on the healthcare center.[24] Transportation can thus include the time and expenses to the families coming into the hospital (or to visit a telemedicine center), or moving within the hospital if the infant remains hospitalized, but they can also reflect the cost of a specialized physician and assistant nurse or other staff category moving within or between hospitals to conduct screening. In addition to being an important cost component to consider in evaluations, the transportation aspect and hotel costs for staying in the hospital can directly affect screening. Our group has clinical experience of parents selecting not to attend planned screening visits after leaving the hospital, so that travel costs also become an issue related to increasing screening adherence and motivating attendance.

Lifetime (treatment and follow-up)

Treatment costs in individual studies included, e.g., staff salaries/time, equipment and maintenance, supplies, and staff training. Few studies reported detailed data on cost components, but Wongwai et al.,[8] for example, reported post-screening resource use of 60 minutes for an expert ophthalmologist, which we interpret to be the cost for treatment. Although case-mix and survival of extremely preterm infants were not detailed in the included studies, it can be expected that these factors will affect how many infants need treatment for ROP. For example, among infants born ≤30 gestational weeks in Sweden, 32% had any stage ROP and 6% were treated for ROP,[28] but among infants born at <24 gestational weeks, the corresponding figures were 92% and 43%.[31] Moreover, the available treatment options would affect costs, with studies suggesting, e.g., more retreatments with the more recent anti-vascular endothelial growth factor (VEGF) therapy.[28] Surgical intervention, or vitrectomy, could also apply to more severe cases,[32] in particular in countries with low access to screening. Although the costs of vitrectomy itself appear to be low,[33] there are likely other costs associated with these severe ROP cases, such as those linked to follow-up and complications.[34]

The argument regarding transportation costs is highly relevant for the treatment of ROP. The clinical reality of many countries is that patients

For peer review only

must be flown to the treatment site, or undergo multiple relocations by ambulance between local hospitals and specialized units providing the treatment.

At least in countries with high access to healthcare, it can be expected that children with ROP, and particularly those with severe forms requiring treatment, will have multiple follow-ups during childhood, adolescence, and possibly into adulthood. The low number of healthcare visits for follow-up indicated in the included articles differs considerably from the national guidelines in Sweden, recommending annual follow-up of ROP until adulthood and, after that, according to need. In a recent publication reporting on a model for predicting visual outcomes after ROP treatment,[35] follow-up every 6 months was even indicated for some patient groups.

Although costs for blindness can be expected to be similar regardless of the cause of blindness, data are available on approximate cost levels for different levels of visual impairment.[36] Thus, tapping into models for measuring costs of visual impairment can add to understanding of the long-term consequences of ROP.

References

1. Evers S, Goossens M, de Vet H, van Tulder M, Ament A. Criteria list for assessment of methodological quality of economic evaluations: Consensus on Health Economic Criteria. *Int J Technol Assess Health Care*. 2005;21(2):240-245.
2. Black L, Hulsey T, Lee K, Parks DC, Ebeling MD. Incremental Hospital Costs Associated With Comorbidities of Prematurity. *Manag Care*. 2015;24(12):54-60.
3. Brown GC, Brown MM, Sharma S, Tasman W, Brown HC. Cost-effectiveness of treatment for threshold retinopathy of prematurity. *Pediatrics*. 1999;104(4):e47.
4. Castillo-Riquelme MC, Lord J, Moseley MJ, Fielder AR, Haines L. Cost-effectiveness of digital photographic screening for retinopathy of prematurity in the United Kingdom. *Int J Technol Assess Health Care*. 2004;20(2):201-213.
5. Javitt J, Cas RD, Chiang YP. Cost-effectiveness of screening and cryotherapy for threshold retinopathy of prematurity. *Pediatrics*. 1993;91(5):859-866.
6. Lee SK, Norm, C., et al. Evidence for changing guidelines for routine screening for retinopathy of prematurity. *Arch Pediatr Adolesc Med*. 2001;155(3):387-395.
7. Rothschild MI, Russ R, Brennan KA, et al. The Economic Model of Retinopathy of Prematurity (EcROP) Screening and Treatment: Mexico and the United States. *Am J Ophthalmol*. 2016;168:110-121.
8. Wongwai P, Kingkaew P, Asawaphureekorn S, Kolatat T. A store-and-forward telemedicine for retinopathy of prematurity screen: Is it cost-effective in Thailand? *Asian Biomedicine*. 2015;9(5):665-673.
9. Dave HB, Gordillo L, Yang Z, Zhang MS, Hubbard GB 3rd, Olsen TW. The societal burden of blindness secondary to retinopathy of prematurity in Lima, Peru. *Am J Ophthalmol*. 2012;154(4):750-755.
10. Dunbar JA, Hsu V, Christensen M, Black B, Williams P, Beauchamp G. Cost-utility analysis of screening and laser treatment of retinopathy of prematurity. *Journal of AAPOS*. 2009;13(2):186-190.
11. Isaac M, Isaranuwatchai W, Tehrani N. Cost analysis of remote telemedicine screening for retinopathy of prematurity. *Can J Ophthalmol*. 2018;53(2):162-167.
12. Kamholz KL, Cole CH, Gray JE, Zupancic JA. Cost-effectiveness of early treatment for retinopathy of prematurity. *Pediatrics*. 2009;123(1):262-269.
13. Jackson KM, Scott KE, Graff Zivin J, et al. Cost-utility analysis of telemedicine and ophthalmoscopy for retinopathy of prematurity management. *Arch Ophthalmol*. 2008;126(4):493-499.

For peer review only

14. Kelkar J, Agashe S, Kelkar A, Kh, ekar R. Mobile unit for retinopathy of prematurity screening and management at urban Neonatal Intensive Care Units: Outcomes and impact assessment. *Oman J Ophthalmol*. 2017;10(1):13-16.

15. Kelkar J, Kelkar A, Sharma S, Dewani J. A mobile team for screening of retinopathy of prematurity in India: Cost - effectiveness, outcomes, and impact assessment. *Taiwan J Ophthalmol*. 2017;7(3):155-159.

16. Mohammadi S-F, Rahban A, Darabeigi S, et al. Cost-effectiveness analysis of tele-retinopathy of prematurity screening in Iran. *Int J Ophthalmol*. 2021;14(4):560-566. doi:10.18240/ijo.2021.04.13

17. Moitry M, Zarca K, Granier M, et al. Effectiveness and efficiency of tele-expertise for improving access to retinopathy screening among 351 neonates in a secondary care center: An observational, controlled before-after study. *PLoS One*. 2018;13(10):e0206375.

18. van den Akker-van Marle ME, van Sorge AJ, Schalij-Delfos NE. Cost and effects of risk factor guided screening strategies for retinopathy of prematurity for different treatment strategies. *Acta Ophthalmol*. 2015;93(8):706-712.

19. Yanovitch TL, Siatkowski RM, McCaffree M, Corff KE. Retinopathy of prematurity in infants with birth weight>or=1250 grams-incidence, severity, and screening guideline cost-analysis. *J aapos*. 2006;10(2):128-134.

20. Zin AA, Magluta C, Pinto MF, et al. Retinopathy of prematurity screening and treatment cost in Brazil. *Rev Panam Salud Publica*. 2014;36(1):37-43.

21. Page MJ, McKenzie JE, Bossuyt PM, et al. The PRISMA 2020 statement: an updated guideline for reporting systematic reviews. *BMJ*. 2021;372:n71. doi:10.1136/bmj.n71

22. Cross KW. Cost of preventing retrolental fibroplasia? *Lancet*. 1973;2(7835):954-956. doi:10.1016/s0140-6736(73)92610-x

23. Boncz I, Kovács LG, Ertl T, Ágoston I, Molics B, Bódis J. Health-economic analysis of diseases related to disturbed neonatal adaptation: A cost of illness study. *Lege Artis Medicinae*. 2013;23(3-4):193-197.

24. Yu T-Y, Donovan T, Armfield N, Gole GA. Retinopathy of prematurity: the high cost of screening regional and remote infants. *Clin Exp Ophthalmol*. 2018;46(6):645-651. doi:10.1111/ceo.13160

25. Scholz SM, Greiner W. An exclusive human milk diet for very low birth weight newborns-A cost-effectiveness and EVPI study for Germany. *PLoS One*. 2019;14(12):e0226496. doi:10.1371/journal.pone.0226496

26. Zupancic JAF, Ying G-S, de Alba Campomanes A, Tomlinson LA, Binenbaum G, G-ROP Study Group. Evaluation of the economic impact of modified screening criteria for retinopathy of prematurity from the Postnatal Growth and ROP (G-ROP) study. *J Perinatol*. 2020;40(7):1100-1108. doi:10.1038/s41372-020-0605-5

27. Blencowe H, Lawn JE, Vazquez T, Fielder A, Gilbert C. Preterm-associated visual impairment and estimates of retinopathy of prematurity at regional and global levels for 2010. *Pediatr Res*. 2013;74(Suppl 1):35-49. doi:10.1038/pr.2013.205

28. Holmström G, Hellström A, Gränse L, et al. New modifications of Swedish ROP guidelines based on 10-year data from the SWEDROP register. *Br J Ophthalmol*. 2020;104(7):943-949. doi:10.1136/bjophthalmol-2019-314874

29. Trzcionkowska K, Groenendaal F, Andriessen P, et al. Risk Factors for Retinopathy of Prematurity in the Netherlands: A Comparison of Two Cohorts. *Neonatology*. 2021;118(4):462-469. doi:10.1159/000517247

30. Isaza G, Arora S. Incidence and severity of retinopathy of prematurity in extremely premature infants. *Canadian Journal of Ophthalmology*. 2012;47(3):296-300. doi:10.1016/j.jcjo.2012.03.027

For peer review only

31. Lundgren P, Jacobson L, Hård A-L, et al. High rate and large intercentre variability in retreatment of retinopathy of prematurity in infants born <24 gestational weeks. *BMJ Open Ophthalmol*. 2021;6(1):e000695. doi:10.1136/bmjophth-2020-000695

32. Rajan RP, Kannan NB, Sen S, et al. Clinico-demographic profile and outcomes of 25-gauge vitrectomy in advanced stage 5 retinopathy of prematurity. *Graefe's Archive for Clinical and Experimental Ophthalmology*. 2021;259(7):1695-1701. doi:10.1007/s00417-020-05063-2

33. Nicod E, Jackson TL, Grimaccia F, et al. Direct cost of pars plana vitrectomy for the treatment of macular hole, epiretinal membrane and vitreomacular traction: a bottom-up approach. *European Journal of Health Economics*. 2016;17(8):991-999. doi:10.1007/s10198-015-0741-6

34. Zhao D-Y, Zhang Y-J, He Z-J, et al. Clinical analysis of apnea after operation for retinopathy of prematurity. *Journal of Shanghai Jiaotong University (Medical Science)*. 2010;30(2):132-134.

35. Huang C-Y, Kuo R-J, Li C-H, et al. Prediction of visual outcomes by an artificial neural network following intravitreal injection and laser therapy for retinopathy of prematurity. *British Journal of Ophthalmology*. 2020;104(9):1277-1282. doi:10.1136/bjophthalmol-2019-314860

36. Köberlein J, Beifus K, Schaffert C, Finger RP. The economic burden of visual impairment and blindness: a systematic review. *BMJ Open*. 2013;3(11):e003471. doi:10.1136/bmjopen-2013-003471

For peer review only

PRISMA checklist

Section and Topic	Item #	Checklist item	Location where item is reported
**TITLE**			
Title	1	Identify the report as a systematic review.	Title
**ABSTRACT**			
Abstract	2	See the PRISMA 2020 for Abstracts checklist.	[See below]
**INTRODUCTION**			
Rationale	3	Describe the rationale for the review in the context of existing knowledge.	Page 3
Objectives	4	Provide an explicit statement of the objective(s) or question(s) the review addresses.	Page 3
**METHODS**			
Eligibility criteria	5	Specify the inclusion and exclusion criteria for the review and how studies were grouped for the syntheses.	Page 3
Information sources	6	Specify all databases, registers, websites, organisations, reference lists and other sources searched or consulted to identify studies. Specify the date when each source was last searched or consulted.	Page 3
Search strategy	7	Present the full search strategies for all databases, registers and websites, including any filters and limits used.	eTable 1
Selection process	8	Specify the methods used to decide whether a study met the inclusion criteria of the review, including how many reviewers screened each record and each report retrieved, whether they worked independently, and if applicable, details of automation tools used in the process.	Page 3
Data collection process	9	Specify the methods used to collect data from reports, including how many reviewers collected data from each report, whether they worked independently, any processes for obtaining or confirming data from study investigators, and if applicable, details of automation tools used in the process.	Page 3
Data items	10a	List and define all outcomes for which data were sought. Specify whether all results that were compatible with each outcome domain in each study were sought (e.g. for all measures, time points, analyses), and if not, the methods used to decide which results to collect.	eTable 2
	10b	List and define all other variables for which data were sought (e.g. participant and intervention characteristics, funding sources). Describe any assumptions made about any missing or unclear information.	eTable 2
Study risk of bias assessment	11	Specify the methods used to assess risk of bias in the included studies, including details of the tool(s) used, how many reviewers assessed each study and whether they worked independently, and if applicable, details of automation tools used in the process.	Page 3
Effect measures	12	Specify for each outcome the effect measure(s) (e.g. risk ratio, mean difference) used in the synthesis or presentation of results.	Page 3
Synthesis methods	13a	Describe the processes used to decide which studies were eligible for each synthesis (e.g. tabulating the study intervention characteristics and comparing against the planned groups for each synthesis (item #5)).	Page 3
	13b	Describe any methods required to prepare the data for presentation or synthesis, such as handling of missing summary statistics, or data conversions.	Page 3
	13c	Describe any methods used to tabulate or visually display results of individual studies and syntheses.	Page 3

For peer review only

Section and Topic	Item #	Checklist item	Location where item is reported
	13d	Describe any methods used to synthesize results and provide a rationale for the choice(s). If meta-analysis was performed, describe the model(s), method(s) to identify the presence and extent of statistical heterogeneity, and software package(s) used.	Page 3
	13e	Describe any methods used to explore possible causes of heterogeneity among study results (e.g. subgroup analysis, meta-regression).	Page 3
	13f	Describe any sensitivity analyses conducted to assess robustness of the synthesized results.	Page 3
Reporting bias assessment	14	Describe any methods used to assess risk of bias due to missing results in a synthesis (arising from reporting biases).	Not possible
Certainty assessment	15	Describe any methods used to assess certainty (or confidence) in the body of evidence for an outcome.	Page 4
**RESULTS**			
Study selection	16a	Describe the results of the search and selection process, from the number of records identified in the search to the number of studies included in the review, ideally using a flow diagram.	eFigure 1
	16b	Cite studies that might appear to meet the inclusion criteria, but which were excluded, and explain why they were excluded.	eTable 4
Study characteristics	17	Cite each included study and present its characteristics.	Page 3
Risk of bias in studies	18	Present assessments of risk of bias for each included study.	eTable 3
Results of individual studies	19	For all outcomes, present, for each study: (a) summary statistics for each group (where appropriate) and (b) an effect estimate and its precision (e.g. confidence/credible interval), ideally using structured tables or plots.	Page 4
Results of syntheses	20a	For each synthesis, briefly summarise the characteristics and risk of bias among contributing studies.	Page 4-5
	20b	Present results of all statistical syntheses conducted. If meta-analysis was done, present for each the summary estimate and its precision (e.g. confidence/credible interval) and measures of statistical heterogeneity. If comparing groups, describe the direction of the effect.	Figure 3
	20c	Present results of all investigations of possible causes of heterogeneity among study results.	Figures 1-3
	20d	Present results of all sensitivity analyses conducted to assess the robustness of the synthesized results.	Not applicable
Reporting biases	21	Present assessments of risk of bias due to missing results (arising from reporting biases) for each synthesis assessed.	Not possible
Certainty of evidence	22	Present assessments of certainty (or confidence) in the body of evidence for each outcome assessed.	Table 2 and Figure 3
**DISCUSSION**			
Discussion	23a	Provide a general interpretation of the results in the context of other evidence.	Page 5-6
	23b	Discuss any limitations of the evidence included in the review.	Page 5-6

For peer review only

Section and Topic	Item #	Checklist item	Location where item is reported
	23c	Discuss any limitations of the review processes used.	Page 5
	23d	Discuss implications of the results for practice, policy, and future research.	Page 6
**OTHER INFORMATION**			
Registration and protocol	24a	Provide registration information for the review, including register name and registration number, or state that the review was not registered.	Page 2
	24b	Indicate where the review protocol can be accessed, or state that a protocol was not prepared.	Page 3
	24c	Describe and explain any amendments to information provided at registration or in the protocol.	Not applicable
Support	25	Describe sources of financial or non-financial support for the review, and the role of the funders or sponsors in the review.	Page 7
Competing interests	26	Declare any competing interests of review authors.	Page 7
Availability of data, code and other materials	27	Report which of the following are publicly available and where they can be found: template data collection forms; data extracted from included studies; data used for all analyses; analytic code; any other materials used in the review.	Page 7

For peer review only

PRISMA abstract checklist

Section and Topic	Item #	Checklist item	Reported (Yes/No)
**TITLE**			
Title	1	Identify the report as a systematic review.	Title
**BACKGROUND**			
Objectives	2	Provide an explicit statement of the main objective(s) or question(s) the review addresses.	Objective
**METHODS**			
Eligibility criteria	3	Specify the inclusion and exclusion criteria for the review.	Study selection
Information sources	4	Specify the information sources (e.g. databases, registers) used to identify studies and the date when each was last searched.	Data sources
Risk of bias	5	Specify the methods used to assess risk of bias in the included studies.	Data Extraction and Synthesis
Synthesis of results	6	Specify the methods used to present and synthesise results.	Data Extraction and Synthesis
**RESULTS**			
Included studies	7	Give the total number of included studies and participants and summarise relevant characteristics of studies.	Results
Synthesis of results	8	Present results for main outcomes, preferably indicating the number of included studies and participants for each. If meta-analysis was done, report the summary estimate and confidence/credible interval. If comparing groups, indicate the direction of the effect (i.e. which group is favoured).	Results
**DISCUSSION**			
Limitations of evidence	9	Provide a brief summary of the limitations of the evidence included in the review (e.g. study risk of bias, inconsistency and imprecision).	Conclusions and Relevance
Interpretation	10	Provide a general interpretation of the results and important implications.	Conclusions and Relevance
**OTHER**			
Funding	11	Specify the primary source of funding for the review.	[In funding statement]
Registration	12	Provide the register name and registration number.	Registration

For peer review only

Section and Topic	Item #	Checklist item	Reported (Yes/No)
			number in PROSPERO

For peer review only

BMJ Open

Costs Associated with Retinopathy of Prematurity: A Systematic Review and Meta-analysis

Journal:	*BMJ Open*
Manuscript ID	bmjopen-2021-057864.R2
Article Type:	Original research
Date Submitted by the Author:	03-Nov-2022
Complete List of Authors:	Gyllensten, Hanna; University of Gothenburg Institute of Health and Care Sciences; University of Gothenburg Centre for Person-Centred Care<br>Humayun, Jhangir; University of Gothenburg Institute of Health and Care Sciences; University of Gothenburg Centre for Person-Centred Care<br>Sjöbom, Ulrika; University of Gothenburg Institute of Health and Care Sciences; University of Gothenburg, Department of Clinical Neuroscience<br>Hellström, Ann; University of Gothenburg Institute of Neuroscience and Physiology, Neuroscience<br>Löfqvist, Chatarina; University of Gothenburg Institute of Health and Care Sciences, Sahlgrenska Academy; University of Gothenburg Institute of Neuroscience and Physiology, Department of Clinical Neuroscience
**Primary Subject Heading**:	Ophthalmology
Secondary Subject Heading:	Health economics, Ophthalmology, Paediatrics
Keywords:	HEALTH ECONOMICS, Paediatric ophthalmology < OPHTHALMOLOGY, Medical retina < OPHTHALMOLOGY

SCHOLARONE™
Manuscripts

I, the Submitting Author has the right to grant and does grant on behalf of all authors of the Work (as defined in the below author licence), an exclusive licence and/or a non-exclusive licence for contributions from authors who are: i) UK Crown employees; ii) where BMJ has agreed a CC-BY licence shall apply, and/or iii) in accordance with the terms applicable for US Federal Government officers or employees acting as part of their official duties; on a worldwide, perpetual, irrevocable, royalty-free basis to BMJ Publishing Group Ltd ("BMJ") its licensees and where the relevant Journal is co-owned by BMJ to the co-owners of the Journal, to publish the Work in this journal and any other BMJ products and to exploit all rights, as set out in our licence.

The Submitting Author accepts and understands that any supply made under these terms is made by BMJ to the Submitting Author unless you are acting as an employee on behalf of your employer or a postgraduate student of an affiliated institution which is paying any applicable article publishing charge ("APC") for Open Access articles. Where the Submitting Author wishes to make the Work available on an Open Access basis (and intends to pay the relevant APC), the terms of reuse of such Open Access shall be governed by a Creative Commons licence – details of these licences and which Creative Commons licence will apply to this Work are set out in our licence referred to above.

Other than as permitted in any relevant BMJ Author's Self Archiving Policies, I confirm this Work has not been accepted for publication elsewhere, is not being considered for publication elsewhere and does not duplicate material already published. I confirm all authors consent to publication of this Work and authorise the granting of this licence.

For peer review only

Title page

Costs Associated with Retinopathy of Prematurity: A Systematic Review and Meta-analysis

Authors

Hanna Gyllensten[1,2] Associate professor

Jhangir Humayun[1,2], B.Sc.

Ulrika Sjöbom[1,3], M.Sc.

Ann Hellström[3] Professor

Chatarina Löfqvist[1,2,3] Associate professor

Author affiliations

[1] Institute of Health and Care Sciences, Sahlgrenska Academy, University of Gothenburg, Box 457, SE-405 30 Gothenburg, Sweden.

[2] University of Gothenburg Centre for Person-Centred Care (GPCC), Sahlgrenska Academy, University of Gothenburg, Box 457, SE-405 30 Gothenburg, Sweden.

[3] Department of Clinical Neuroscience, Institute of Neuroscience and Physiology, Sahlgrenska Academy, University of Gothenburg, Gothenburg, Sweden;

Contact, corresponding author

Hanna Gyllensten, Institute of Health and Care Sciences, Sahlgrenska Academy, University of Gothenburg, Postal Address: Box 457, SE-405 30 Gothenburg, Sweden, E-mail: hanna.gyllensten@gu.se. Phone: +46-(0)70-748 24 12

Keywords

Retinopathy of Prematurity; Costs and Cost Analysis; Systematic Review; Meta-Analysis.

Word count (excluding title page, abstract, references, figures and tables): 2644

For peer review only - http://bmjopen.bmj.com/site/about/guidelines.xhtml

Abstract

Objectives To review and analyze evidence regarding costs for retinopathy of prematurity (ROP) screening, lifetime costs and resource use among infants born preterm who develop ROP, and how these costs have developed over time in different regions.

Design Systematic review and meta-analysis

Data sources PubMed and Scopus from inception to June 23, 2021.

Eligibility criteria for selecting studies Included studies presented costs for ROP screening and the lifetime costs (including laser treatment and follow-up costs) and resource use among people who develop ROP. Studies not reporting on cost calculation methods or ROP-specific costs were excluded.

Data extraction and synthesis Two independent reviewers screened for inclusion and extracted data, including items from a published checklist for quality assessment used for bias assessment, summary, and random-effects meta-analysis for treatment costs. Included studies were further searched to identify eligible references and citations.

Results In total, 15 studies reported ROP screening costs, and 13 reported lifetime costs (either treatment and/or follow-up costs) for infants with ROP. The range for screening costs (10 studies) was US$5–$253 per visit, or US$324–$1072 per screened child (5 studies). Costs for treatment (11 studies) ranged from US$38 to US$6500 per child. Four studies reported healthcare follow-up costs (lifetime costs ranging from US$64–US$2420, and 10 year-costs of US$1695, respectively), and of these, three also reported lifetime costs for blindness (range US$26,686–US$224,295) using secondary cost data. Included papers largely followed the quality assessment checklist items, thus indicating a low risk of bias.

Conclusion The costs of screening for and treating ROP are small compared to the societal costs of resulting blindness. However, little evidence is available for predicting the effects of changes in patient population, screening schedule, or ROP treatments.

For peer review only - http://bmjopen.bmj.com/site/about/guidelines.xhtml

PROSPERO registration number CRD42020208213.

Strengths and limitations of this study

- PubMed and Scopus were searched systematically.
- Since manual search of reference lists and citations of the identified papers did not identify additional studies, the database search had good coverage of the topic of investigation.
- The main limitations of this work were the exclusion of grey literature and the lack of analyses of publication bias for the meta-analysis.
- Where lack of variance information in included studies hindered meta-analysis, guidance for synthesis in systematic reviews without meta-analyses were followed.

For peer review only

Introduction

Improvements in neonatal care have resulted in increased survival among children born preterm,[1] but these infants are at risk of developing preterm-related morbidities such as retinopathy of prematurity (ROP). ROP is characterized by abnormal neurovascular development and, in its worst forms, retinal detachment and blindness.[2] Although preventable, ROP is the leading cause of blindness in children worldwide,[3] a ranking associated with the survival of infants with extremely low gestational age and birth weight in some parts of the world, and use of unmonitored treatments with 100% oxygen in other regions.[2]

ROP management and treatment economics are still challenging in many health systems because of screening-associated costs, patient-related costs, and medico-legal liability.[4] Thus, there is an urgent need for more concerted efforts to guide healthcare providers in how to use resources efficiently, both in developing economies during a phase of improving survival of preterm infants, such as in many parts of Africa [5], and in countries like Sweden with major neonatal morbidities still affecting a large proportion of those who survive.[6]

Here we present an overview of costs associated with ROP screening and treatment, examining the evidence related to costs for ROP screening and lifetime costs (including laser treatment and follow-up costs) and resource use among infants born preterm who develop ROP. We also examine the trajectories of these costs over time in different regions in a meta-analysis.

Methods

This work followed the Preferred Reporting Items for Systematic Review and Meta-Analysis (i.e., PRISMA),[7] with protocol available in PROSPERO (reference CRD42020208213).[8]

For peer review only - http://bmjopen.bmj.com/site/about/guidelines.xhtml

Article search

Pubmed and Scopus were searched (eTable 1, 23 Jun 2021) to identify original research on costs for ROP, including full cost or cost increases associated with ROP, without restricting language, publication date, or country. Papers were thus included if presenting costs for ROP screening or lifetime costs (including laser treatment and follow-up costs) and resource use among people who develop ROP. Lifetime costs can for example include follow-up healthcare costs but also productivity loss due to blindness or other cost components occurring due to visual impairment later in life. Articles that did not describe the cost calculation method were excluded, as were those not presenting the costs for the group with ROP separately.

Rayyan QCRI was used for handling duplicates and the selection of studies for inclusion. Two independent reviewers (JH and CL or HG) searched the databases, screened articles for eligibility, extracted data using a pre-specified data extraction sheet (eTable 2), and hand-searched included studies (7 July 2021) to identify eligible references and citations. Conflicting views were resolved by discussion with a third reviewer (CL or HG).

The data extraction sheet included items (eTable 2) from a published checklist for quality assessment of economic evaluations[9] including a core set of items relevant in assessing the risk of bias in included studies. The 19 checklist items covers design and methods, population and generalizability, as well as ethics and funding, answered as yes or no during the assessment. To aid reading, summary scores indicating the items answered as Yes for each paper were calculated, thus a high summary score indicates that many of the items were covered. Quality of evidence was rated on a scale from 1 to 5 for individual articles, according to: 1=e.g., properly powered randomized controlled trials; 2=e.g., prospective cohort studies; 3=e.g., retrospective cohort studies; 4=case series with or without intervention or cross-sectional study; 5=e.g., opinion of respected authorities.[10]

For peer review only - http://bmjopen.bmj.com/site/about/guidelines.xhtml

Analysis

Conventional screening (excluding telemedicine costs), laser treatment, and long-term follow-up costs were reported, respectively, accounting for ROP severity and differences over time and between countries. Identified costs were adjusted to 2020 US dollars (US$) using annual exchange rates[11] and the Organisation for Economic Co-operation and Development inflation factor.[12] After imputation of missing variance based on the percentage variance in studies presenting such information, treatment costs were summarized in a forest plot, by year and subgroups using the World Bank country classification based on gross national income per capita,[13] as cost levels can be expected to differ.

Patient and public involvement

This project did not include patient or public involvement in developing the research questions, design, conduct, choice of outcome measures, or recruitment.

Results

Of the 503 studies screened after duplicates from the databases were removed, 123 were assessed for eligibility based on full text, and 19 studies were included in the synthesis of results (eFigure 1). Reasons for exclusion were absence of data on costs associated with ROP, lack of original data, or inclusion of data related only to insurance payments or litigation. No additional studies were identified by a hand search of references and a Scopus search of citations of included studies. An overview of all included studies[14–32] is presented in Table 1, including references to secondary cost sources.[33–39] In total, 15 studies covered screening costs and 13 reported lifetime costs (treatment and/or follow-up costs) for infants who developed ROP.

For peer review only - http://bmjopen.bmj.com/site/about/guidelines.xhtml

Twelve studies were conducted in high-income economies: seven in the United States, two in Canada, and one each in the United Kingdom, Netherlands, and France. Three studies were conducted in upper-middle income economies: one each in Peru, Thailand, and Brazil. Three studies were conducted in lower-middle income economies: two in India and one in Iran. One study was conducted in both the United States and Mexico (Table 1). All studies reported the economic analyses using either US dollars, euros, or local currency. The patient populations in all studies were infants at risk for ROP, although the studies used different inclusion criteria based on gestational age at birth and birth weight. In addition, the ROP definition for stages and treatment criteria varied with the timing of the study and international guidelines for classification at that time.

Risk of bias in included studies

The quality assessment indicated a high overall quality of the included studies (eTable 3), with 16 of 19 of them fulfilling at least 16 of the assessed criteria. However, eight studies did not fulfill the criteria for discounting future costs and outcomes or for subjecting results to sensitivity analyses to address the effects of assumptions. Additionally, 14 studies met criteria regarding the reporting of incremental analysis and potential conflicts of interest. Thus, overall, the assessment suggested a low risk of bias in the included papers, and also indicated where lack of reporting on potential conflicts of interest was most problematic. Quality of evidence ranged from 1 to 5 for individual articles, with articles most commonly based on data from retrospective cohort studies (evidence rating 3; 9 publications).

Costs for ROP screening

Studies reporting costs related to screening had different designs: six were retrospective cohort studies using medical chart review or register data,[15,16,20,24,28,30] nine developed

For peer review only - http://bmjopen.bmj.com/site/about/guidelines.xhtml

economic models,[19,21,23,25–27,29,31,32] and two were public intervention studies related to the introduction of ROP screening programs.[17,18] Although the assessment indicated a low risk of bias, screening costs differed substantially among reporting countries (Figure 1a).

Costs for routine ROP screening, excluding transportation costs, are reported in Table 2. Ten studies reported a mean unit cost per screening of US$137 (range: 5–253). In addition, five studies reported a mean cost per screened child of US$553 (range: 324–1072). Of these, two studies reported comparably low costs[21,23] for staff and equipment, whereas Rothchild et al.[19] reported comparably higher costs in the US setting. One study also included transportation costs,[15] and when these costs were removed, screening cost was comparably low. The other studies reported similar costs for screening per child (range: US$324–$602).[25,28,29]

Javitt et al.[32] reported a mean unit cost of US$183 for a first screening and of US$149 for follow-up screening, whereas Lee et al.[30] reported a mean unit cost of US$112 for screening one eye. Finally, two studies from India[17,18] reported screening costs of US$1003 and US$630, respectively, for identifying one child with ROP.

In studies comparing alternative screening or treatment options, no common comparator was identified. The incremental cost reported in Black et al.[22] indicated a savings associated with higher gestational age at birth (Table 1). Jackson et al.[27] used economic modeling to estimate the cost-utility of ROP screening using telemedicine vs. conventional ROP screening. Javitt et al.[32] used modeling to compare weekly, biweekly, or monthly screening.

Costs for ROP treatment

In all, 14 studies reported costs related to the laser treatment of ROP (Figure 1b). Four studies of treatment costs were retrospective cohort studies,[20,24,28,30] eight were modeling

For peer review only

For peer review only - http://bmjopen.bmj.com/site/about/guidelines.xhtml

studies,[14,19,21,23,25,26,29,31] and two were public intervention studies.[17,18] In addition, two of the included studies[31,32] reported costs for cryotherapy (not included in the analyses below). Eleven studies reported total treatment costs per child, at a mean US$2442 (range: 38–6500). Castillo-Riquelme et al.[29] found unilateral treatment costs up to US$1165 and bilateral treatment costs up to US$1514, based partially on secondary data from Brown et al.[31] Two studies[20,26] cited unit costs of laser treatment of US$4065 and US$5661, respectively. Laser treatment costs are reported in Table 2. Dave et al.[24] described costs for screening and treatment combined (US$2962) in a cohort of children with blindness.

Accounting for the low assessed risk of bias but large expected variation based on cost-levels of individual countries, the meta-analysis by country classification (Figures 2-3) estimated the average costs in high-income economies to US$2960 (95% confidence interval [CI]: 2003–3917). Corresponding figures were US$329 (95% CI: 9–649) in upper-middle–income economies and US$3692 (95% CI: 670–6715) in lower-middle–income economies, respectively. Most studies did not report variance of results, making publication bias analysis unfeasible. However, model diagnostics (I^2 and Cochrane Q) indicated high heterogeneity between studies within each country classification, which suggests that the results from the meta-analysis should be interpreted with caution.

Follow-up costs and resource use among infants born preterm and developing ROP

Only four studies reported follow-up costs occurring after screening and treatment, and although the risk of bias was assessed as low, the reported results largely differed between studies. Castillo-Riquelme et al.[29] reported healthcare follow-up costs over 10 years of up to US$1695. Dave et al.[24] reported a lifetime follow-up visit cost of US$64 and a blindness cost of US$146,952. Rothchild et al.[19] reported lifetime follow-up healthcare costs of US$1681 (US) and US$2420 (Mexico), whereas the costs for blindness were estimated to be

For peer review only - http://bmjopen.bmj.com/site/about/guidelines.xhtml

US$92,460 (US) and US$26,686 (Mexico). Wongwai et al.[21] reported the lifetime costs of blindness to be $224,295. In addition, Black et al.[22] reported the costs per quality-adjusted life-year (QALY) associated with ROP and other comorbidities associated with being born preterm.

Discussion

The studies we identified could be grouped by whether they reported costs for screening, costs for treatment, or costs (and QALYs) during long-term follow-up or even from a lifetime perspective. The cost range per ROP screening was US$5–$253 per visit, or US$324–$1072 per screened child. Costs for ROP treatment ranged from US$38–$6500 per child. In addition, four studies reported healthcare follow-up costs, and three reported lifetime costs using secondary data on costs for blindness. Although quality assessment indicated a low risk of bias, comparisons between studies were challenging because of the lack of detailed cost and resource use data.

To our knowledge, this is the first systematic review of ROP costs. Included papers largely followed the quality assessment checklist items of a commonly used tool,[40] thus indicating a low risk of bias. However, few of the included articles reported disaggregated cost and resource use data or detailed the included cost components, as is recommended for economic evaluations.[41] The main limitations of this work were the exclusion of grey literature and the lack of analyses of publication bias for the meta-analysis. Guidance for reliability in systematic reviews of retinal disorder interventions[42] was fulfilled, but the standards for systematic reviews of costs and cost-effectiveness studies were not due to the lack of grey literature assessment.[43] Also, since costs were reported purely in a descriptive manner no sensitivity analyses were conducted for alternative categorizations of cost components or country classifications. While not a limitation specific to this analysis but

For peer review only - http://bmjopen.bmj.com/site/about/guidelines.xhtml

rather of the lack of variance information in the included papers, the findings from the meta-analysis of treatment costs needs to be interpreted with caution after variance was imputed. This lack of variance information also made meta-analysis of screening costs unattainable, since no basis for imputation was available. Moreover, the search strategy and databases are expected to cover largely English-language literature and was limited to only two databases, but the reference and citation search yielded no additional studies to include. We thus expect our findings to represent a good overview of the available evidence, and that regardless the reservations associated with the meta-analysis to represent current knowledge about costs related to screening and treatment of ROP.

Cost components for ROP screening included staff salaries/time, equipment and maintenance, supplies, and staff training. Screening costs for ROP were low compared to other associated costs and, with few exceptions, of the same order of magnitude in the included studies. Exceptions were probably attributable to salary differences.

Screening access and schedules vary between countries.[44] With the possible exception of Javitt et al.,[32], the included studies provided little evidence for how case-mix and alternative screening schedules affect costs for screening. Savings are expected, however, and a modeling study using published cost data calculated an annual cost savings from reduced screening of US$3 million in the United States.[45] However, with low screening costs, the main benefit is reduced discomfort for the infants and reduced travel costs (which can be substantial[15]). The most considerable potential for savings on screening is probably increasing gestational age. US data indicate that ROP frequency increased over time, particularly in infants born very preterm,[46] and infants of lower gestational age usually both require more screening visits and have more severe ROP.[47] Potential savings have been reported from screening using telemedicine (compared to transporting infants to a specialized

For peer review only - http://bmjopen.bmj.com/site/about/guidelines.xhtml

hospital),[15] or using bedside screening with mobile equipment instead of moving the infants to a specific screening facility[48]; however, this review did not consider these aspects.

Treatment costs were low compared to the costs for follow-up, with Brazil, Mexico, and Peru having substantially lower treatment costs than the other countries. Both Javitt et al.[32] and Brown et al.[31] reported low costs for the historically used cryo treatment, at approximately 63% of that for laser treatment. For laser treatment, the cost range was US$2304–$6864 per treated child. None of the studies included the more recent anti-vascular endothelial growth factor (VEGF) therapy. Moreover, no study reported costs based on ROP stages, age of treated infants, or plus disease status.[49] Thus, studies provide little guidance on how treatment costs will develop over time as more infants of lower gestational age survive.

Variation among studies in whether one or two eyes were treated made comparisons less relevant, which may reflect the unilateral schedule used in the historically influential Cryo-ROP study.[50] However, Swedish registers indicate that bilateral treatment is common (76% of initial treatments and 97% overall)[47] and that retreatment is more frequent among infants with very low gestational age[51] and those treated exclusively with anti-VEGF.[47]

When examining ROP treatment, cost components included staff salaries/time, equipment and maintenance, supplies, and staff training. Sometimes anesthesia costs were reported separately or excluded. Transportation was also a considerable cost component in relation to treatment.[20] Other potential costs that were not measured include those for the added time spent in hospital or intensive care, including parental leave, during treatment. Many studies reported only total charges, which are expected to be higher than costs to the healthcare provider. However, use of charges as opposed to costs was not an obvious cause of variation here. Two studies from India[17,18] reported high costs compared to other studies of both costs and charges, possibly because of some transportation costs remaining as part of

For peer review only - http://bmjopen.bmj.com/site/about/guidelines.xhtml

additional components. Thus the apparent decrease in costs over time in the lower-middle-income economies seen in the meta-analysis should be interpreted with caution.

Although ROP results in high costs throughout life, this outcome is primarily based on secondary data for blindness. As the leading cause of preventable childhood blindness[52] and probably the leading cause of childhood blindness in middle-income countries,[53] ROP should be associated with much of the estimated costs of blindness. Moreover, it has been argued that costs for blindness do not differ by cause.[54] Little evidence was available on follow-up after successful, or partially successful, treatment of ROP. Dave et al.[24] indicated three healthcare visits over the first 7 years of life, whereas Castillo-Riquelme et al.[29] did not differentiate visits based on treatment or ROP stage. Rothchild et al. included transportation costs, white canes, Braille equipment, and supplies,[19] but disregarded other costs among children retaining sight. Thus, although costs differ by the severity of visual impairment,[55] studies of ROP costs do not tend to report this more detailed level of sight. The current knowledge does not inform potential savings or inform subsidy decisions for ROP treatment developments that can save a little more sight. Taken together, the short follow-up underestimates the total impact of blindness,[56] and not accounting for visual impairment results in underestimating the financial impact of ROP.

There is a need for comprehensive knowledge about the costs of ROP, both during the introduction of new ROP screening programs and in countries with established programs that are now redistributing resources to handle the increasing survival of very preterm infants with high disease burden. In addition to relevant cost components of ROP (eFigure 2), complementary studies of the benefits of various neonatal preventative strategies, including oxygen delivery, are warranted because evidence of the costs resulting from conditions such as bronchopulmonary dysplasia is also lacking.[57] Such studies should follow state-of-the-art methods for conduct and reporting of health economic studies.

For peer review only

Conclusions

Although costs of screening and treating ROP are substantial for health systems, they are small compared to the follow-up costs to society of resulting blindness. However, little evidence is available to support predictions about the consequences of changes in the patient population, screening schedule, or treatment regimens for ROP.

For peer review only

For peer review only - http://bmjopen.bmj.com/site/about/guidelines.xhtml

FUNDING STATEMENT

HG was financed by the Swedish Research Council (#2016-01131). JH was financed by the University of Gothenburg Centre for Person-Centred Care (GPCC), a teaching assistant program. AH was supported by The Wallenberg Clinical Scholars, The Swedish Research Council (#2020-01092), the Gothenburg County Council (ALF project, #426531), the Gothenburg Medical Society, and De Blindas Vänner, and CL was financed by the GPCC. The funders had no role in the design of the study or writing of the protocol.

COMPETING INTERESTS STATEMENT

HG is employed part-time by IQVIA, which is a contract research organization that performs commissioned pharmacoepidemiological studies. Thus, its employees have been and currently are working in collaboration with several pharmaceutical companies. JH reports no competing interests. AH holds stock/stock options in Premalux AB and has received consulting fees from Takeda Inc. CL holds stocks in Premalux AB.

CONTRIBUTIONS

All authors contributed to the design of the study. HG, JH, and CL designed the database search and data extraction methods. JH and CL undertook the literature search, assessed studies for eligibility, and extracted data. In case of disagreement, assessments were made in discussion with HG. AH contributed clinical expertise on preterm infants and morbidity. HG, JH, US, and CL discussed the data and interpreted the results. HG, JH, and CL drafted the manuscript. All authors critically reviewed and approved the final manuscript. HG had full access to all the data in the study and takes responsibility for the integrity of the data and the accuracy of the data analysis.

ACKNOWLEDGEMENTS

Thanks to SF Edit, a professional scientific-editing service, for language editing.

For peer review only - http://bmjopen.bmj.com/site/about/guidelines.xhtml

DATA AVAILABILITY STATEMENT

All data relevant to the study are included in the article. Original data are available in the reviewed publications, which are all cited. Additional data from the data extraction performed are available on reasonable request from the corresponding author, including author template data collection forms, data extracted from included studies, data used for all analyses, analytic code, and any other materials used in the review.

ETHICS APPROVAL STATEMENT

Not applicable.

For peer review only

For peer review only - http://bmjopen.bmj.com/site/about/guidelines.xhtml

References

1. Lawn JE, Davidge R, Paul VK, et al. Born too soon: care for the preterm baby. *Reprod Health*. 2013;10 Suppl 1:S5. doi:10.1186/1742-4755-10-S1-S5

2. Hellström A, Smith LEH, Dammann O. Retinopathy of prematurity. *Lancet*. 2013;382(9902):1445-1457. doi:10.1016/S0140-6736(13)60178-6

3. Yonekawa Y, Thomas BJ, Thanos A, et al. THE CUTTING EDGE OF RETINOPATHY OF PREMATURITY CARE: Expanding the Boundaries of Diagnosis and Treatment. *Retina (Philadelphia, Pa)*. 2017;37(12):2208-2225. doi:10.1097/IAE.0000000000001719

4. Moshfeghi DM, Capone A. Economic Barriers in Retinopathy of Prematurity Management. *Ophthalmol Retina*. 2018;2(12):1177-1178. doi:10.1016/j.oret.2018.10.002

5. Dupe S. Ademola-Popoola, Tunji S. Oluleye. Retinopathy of Prematurity (ROP) in a Developing Economy with Improving Health Care | SpringerLink. *Current Ophtalmology Reports*. 2017;5:114-118. doi:https://doi-org.ezproxy.ub.gu.se/10.1007/s40135-017-0129-0

6. Norman M, Hallberg B, Abrahamsson T, et al. Association Between Year of Birth and 1-Year Survival Among Extremely Preterm Infants in Sweden During 2004-2007 and 2014-2016. *JAMA*. 2019;321(12):1188-1199. doi:10.1001/jama.2019.2021

7. Page MJ, McKenzie JE, Bossuyt PM, et al. The PRISMA 2020 statement: an updated guideline for reporting systematic reviews. *BMJ*. 2021;372:n71. doi:10.1136/bmj.n71

8. PROSPERO International prospective register of systematic reviews. NIHR, National INstitute for Health Research. https://www.crd.york.ac.uk/prospero/

9. Evers S, Goossens M, de Vet H, van Tulder M, Ament A. Criteria list for assessment of methodological quality of economic evaluations: Consensus on Health Economic Criteria. *Int J Technol Assess Health Care*. 2005;21(2):240-245.

10. Oxford Centre for Evidence-Based Medicine: Levels of Evidence (March 2009). Centre for Evidence-Based Medicine (CEBM), University of Oxford. Accessed August 30, 2022. https://www.cebm.ox.ac.uk/resources/levels-of-evidence/oxford-centre-for-evidence-based-medicine-levels-of-evidence-march-2009

11. Annual average exchange rates (aggregate). Sveriges Riksbank. Accessed June 8, 2021. https://www.riksbank.se/en-gb/statistics/search-interest--exchange-rates/annual-average-exchange-rates/

12. Prices - Inflation (CPI) - OECD Data. Organisation for Economic Co-operation and Development, OECD. Accessed June 9, 2021. http://data.oecd.org/price/inflation-cpi.htm

13. World Bank Country and Lending Groups – World Bank Data Help Desk. Accessed May 13, 2021. https://datahelpdesk.worldbank.org/knowledgebase/articles/906519-world-bank-country-and-lending-groups

14. Mohammadi SF, Rahban A, Darabeigi S, et al. Cost-effectiveness analysis of tele-retinopathy of prematurity screening in Iran. *Int J Ophthalmol*. 2021;14(4):560-566. doi:10.18240/ijo.2021.04.13

15. Moitry M, Zarca K, Granier M, et al. Effectiveness and efficiency of tele-expertise for improving access to retinopathy screening among 351 neonates in a secondary care center: An observational, controlled before-after study. *PLoS One*. 2018;13(10):e0206375.

16. Isaac M, Isaranuwatchai W, Tehrani N. Cost analysis of remote telemedicine screening for retinopathy of prematurity. *Can J Ophthalmol*. 2018;53(2):162-167.

For peer review only - http://bmjopen.bmj.com/site/about/guidelines.xhtml

For peer review only

17. Kelkar J, Agashe S, Kelkar A, Kh, ekar R. Mobile unit for retinopathy of prematurity screening and management at urban Neonatal Intensive Care Units: Outcomes and impact assessment. *Oman J Ophthalmol*. 2017;10(1):13-16.

18. Kelkar J, Kelkar A, Sharma S, Dewani J. A mobile team for screening of retinopathy of prematurity in India: Cost - effectiveness, outcomes, and impact assessment. *Taiwan J Ophthalmol*. 2017;7(3):155-159.

19. Rothschild MI, Russ R, Brennan KA, et al. The Economic Model of Retinopathy of Prematurity (EcROP) Screening and Treatment: Mexico and the United States. *Am J Ophthalmol*. 2016;168:110-121.

20. van den Akker-van Marle ME, van Sorge AJ, Schalij-Delfos NE. Cost and effects of risk factor guided screening strategies for retinopathy of prematurity for different treatment strategies. *Acta Ophthalmol*. 2015;93(8):706-712.

21. Wongwai P, Kingkaew P, Asawaphureekorn S, Kolatat T. A store-and-forward telemedicine for retinopathy of prematurity screen: Is it cost-effective in Thailand? *Asian Biomedicine*. 2015;9(5):665-673.

22. Black L, Hulsey T, Lee K, Parks DC, Ebeling MD. Incremental Hospital Costs Associated With Comorbidities of Prematurity. *Manag Care*. 2015;24(12):54-60.

23. Zin AA, Magluta C, Pinto MF, et al. Retinopathy of prematurity screening and treatment cost in Brazil. *Rev Panam Salud Publica*. 2014;36(1):37-43.

24. Dave HB, Gordillo L, Yang Z, Zhang MS, Hubbard GB 3rd, Olsen TW. The societal burden of blindness secondary to retinopathy of prematurity in Lima, Peru. *Am J Ophthalmol*. 2012;154(4):750-755.

25. Dunbar JA, Hsu V, Christensen M, Black B, Williams P, Beauchamp G. Cost-utility analysis of screening and laser treatment of retinopathy of prematurity. *Journal of AAPOS*. 2009;13(2):186-190.

26. Kamholz KL, Cole CH, Gray JE, Zupancic JA. Cost-effectiveness of early treatment for retinopathy of prematurity. *Pediatrics*. 2009;123(1):262-269.

27. Jackson KM, Scott KE, Graff Zivin J, et al. Cost-utility analysis of telemedicine and ophthalmoscopy for retinopathy of prematurity management. *Arch Ophthalmol*. 2008;126(4):493-499.

28. Yanovitch TL, Siatkowski RM, McCaffree M, Corff KE. Retinopathy of prematurity in infants with birth weight>or=1250 grams-incidence, severity, and screening guideline cost-analysis. *J aapos*. 2006;10(2):128-134.

29. Castillo-Riquelme MC, Lord J, Moseley MJ, Fielder AR, Haines L. Cost-effectiveness of digital photographic screening for retinopathy of prematurity in the United Kingdom. *Int J Technol Assess Health Care*. 2004;20(2):201-213.

30. Lee SK, Norm, C., et al. Evidence for changing guidelines for routine screening for retinopathy of prematurity. *Arch Pediatr Adolesc Med*. 2001;155(3):387-395.

31. Brown GC, Brown MM, Sharma S, Tasman W, Brown HC. Cost-effectiveness of treatment for threshold retinopathy of prematurity. *Pediatrics*. 1999;104(4):e47.

32. Javitt J, Cas RD, Chiang YP. Cost-effectiveness of screening and cryotherapy for threshold retinopathy of prematurity. *Pediatrics*. 1993;91(5):859-866.

33. Frick KD, Foster A. The magnitude and cost of global blindness: an increasing problem that can be alleviated. *Am J Ophthalmol*. 2003;135(4):471-476. doi:10.1016/s0002-9394(02)02110-4

34. Christ SL, Lee DJ, Lam BL, Zheng DD, Arheart KL. Assessment of the effect of visual impairment on mortality through multiple health pathways: structural equation modeling. *Invest Ophthalmol Vis Sci*. 2008;49(8):3318-3323. doi:10.1167/iovs.08-1676

For peer review only

For peer review only - http://bmjopen.bmj.com/site/about/guidelines.xhtml

35. United States, Central Intelligence Agency, United States, Central Intelligence Agency. *The World Factbook 2009 (CIA's 2008 Edition)*. Potomac Books; 2008.

36. Palmer EA, Flynn JT, Hardy RJ, et al. Incidence and early course of retinopathy of prematurity. The Cryotherapy for Retinopathy of Prematurity Cooperative Group. *Ophthalmology*. 1991;98(11):1628-1640. doi:10.1016/s0161-6420(91)32074-8

37. Good WV, Hardy RJ, Dobson V, et al. The incidence and course of retinopathy of prematurity: findings from the early treatment for retinopathy of prematurity study. *Pediatrics*. 2005;116(1):15-23. doi:10.1542/peds.2004-1413

38. Cryotherapy for Retinopathy of Prematurity Cooperative Group. Multicenter Trial of Cryotherapy for Retinopathy of Prematurity: ophthalmological outcomes at 10 years. *Arch Ophthalmol*. 2001;119(8):1110-1118. doi:10.1001/archopht.119.8.1110

39. Resnikoff S, Pascolini D, Etya'ale D, et al. Global data on visual impairment in the year 2002. *Bull World Health Organ*. 2004;82(11):844-851. doi:/S0042-96862004001100009

40. Watts RD, Li IW. Use of Checklists in Reviews of Health Economic Evaluations, 2010 to 2018. *Value Health*. 2019;22(3):377-382. doi:10.1016/j.jval.2018.10.006

41. Husereau D, Drummond M, Petrou S, et al. Consolidated Health Economic Evaluation Reporting Standards (CHEERS) statement. *Value Health*. 2013;16(2):e1-5. doi:10.1016/j.jval.2013.02.010

42. Le JT, Qureshi R, Twose C, et al. Evaluation of Systematic Reviews of Interventions for Retina and Vitreous Conditions. *JAMA Ophthalmol*. 2019;137(12):1399-1405. doi:10.1001/jamaophthalmol.2019.4016

43. Mandrik OL, Severens JLH, Bardach A, et al. Critical Appraisal of Systematic Reviews With Costs and Cost-Effectiveness Outcomes: An ISPOR Good Practices Task Force Report. *Value Health*. 2021;24(4):463-472. doi:10.1016/j.jval.2021.01.002

44. Blencowe H, Lawn JE, Vazquez T, Fielder A, Gilbert C. Preterm-associated visual impairment and estimates of retinopathy of prematurity at regional and global levels for 2010. *Pediatr Res*. 2013;74(Suppl 1):35-49. doi:10.1038/pr.2013.205

45. Zupancic JAF, Ying GS, de Alba Campomanes A, Tomlinson LA, Binenbaum G, G-ROP Study Group. Evaluation of the economic impact of modified screening criteria for retinopathy of prematurity from the Postnatal Growth and ROP (G-ROP) study. *J Perinatol*. 2020;40(7):1100-1108. doi:10.1038/s41372-020-0605-5

46. Aly H, Othman HF, Munster C, Das A, Sears J. The US National Trend for Retinopathy of Prematurity. *Am J Perinatol*. Published online February 16, 2021. doi:10.1055/s-0041-1723830

47. Holmström G, Hellström A, Gränse L, et al. New modifications of Swedish ROP guidelines based on 10-year data from the SWEDROP register. *Br J Ophthalmol*. 2020;104(7):943-949. doi:10.1136/bjophthalmol-2019-314874

48. Kovács G, Somogyvári Z, Maka E, Nagyjánosi L. Bedside ROP screening and telemedicine interpretation integrated to a neonatal transport system: Economic aspects and return on investment analysis. *Early Hum Dev*. 2017;106-107:1-5. doi:10.1016/j.earlhumdev.2017.01.007

49. Agarwal K, Jalali S. Classification of retinopathy of prematurity: from then till now. *Community Eye Health*. 2018;31(101):S4-S7.

50. Nguyen QD, Tawansy K, Hirose T. Recent advances in retinopathy of prematurity. *Int Ophthalmol Clin*. 2001;41(4):129-151. doi:10.1097/00004397-200110000-00013

For peer review only - http://bmjopen.bmj.com/site/about/guidelines.xhtml

51. Lundgren P, Jacobson L, Hård AL, et al. High rate and large intercentre variability in retreatment of retinopathy of prematurity in infants born <24 gestational weeks. *BMJ Open Ophthalmol*. 2021;6(1):e000695. doi:10.1136/bmjophth-2020-000695

52. Shah PK, Prabhu V, Karandikar SS, Ranjan R, Narendran V, Kalpana N. Retinopathy of prematurity: Past, present and future. *World J Clin Pediatr*. 2016;5(1):35-46. doi:10.5409/wjcp.v5.i1.35

53. Vision impairment and blindness. World Health Organization. Accessed August 26, 2021. https://www.who.int/news-room/fact-sheets/detail/blindness-and-visual-impairment

54. Naguib MM, Soares RR, Anzures R, et al. Regionally Specific Economic Impact of Screening and Treating Retinopathy of Prematurity in Middle-Income Societies in the Philippines. *J Pediatr Ophthalmol Strabismus*. 2019;56(6):388-396. doi:10.3928/01913913-20190925-02

55. Köberlein J, Beifus K, Schaffert C, Finger RP. The economic burden of visual impairment and blindness: a systematic review. *BMJ Open*. 2013;3(11):e003471. doi:10.1136/bmjopen-2013-003471

56. Chakravarthy U, Biundo E, Saka RO, Fasser C, Bourne R, Little JA. The Economic Impact of Blindness in Europe. *Ophthalmic Epidemiol*. 2017;24(4):239-247. doi:10.1080/09286586.2017.1281426

57. Humayun J, Löfqvist C, Ley D, Hellström A, Gyllensten H. Systematic review of the healthcare cost of bronchopulmonary dysplasia. *BMJ Open*. 2021;11(8):e045729. doi:10.1136/bmjopen-2020-045729

For peer review only

For peer review only - http://bmjopen.bmj.com/site/about/guidelines.xhtml

Tables

Table 1. Overview of Studies Included in This Review.

#	First author (year)	Country (study period) Setting	Study design	ROP definition	Sample size (% of infants with ROP treated)	Inclusion criteria	Mean cost per child with ROP (value year and currency as reported in the original publication)	Cost perspective: cost inclusion
1	Mohammadi (2021) [14]	Iran (2017) Data from Farabi eye hospital	Decision Analytical Model from case series	Threshold ROP	Total: 126 ROP: 126	Randomly selected infants with treatment requiring ROP	**Treatment**: US$1107/infant	Unclear perspective: out-of-pocket charges[a]

For peer review only - http://bmjopen.bmj.com/site/about/guidelines.xhtml

2	Moitry (2018) [15]	France (2012 and 2014-2015) Data from two hospitals CHSF and Port-Royal	Retrospective, before-and-after study	Type 1 ROP	Not specified	GA<33 w or BW<1500 g	**Screening**: €37/exam	Health system: direct costs
3	Isaac (2018) [16]	Canada (2009–2014) Data from Ontario Ministry of Health and Long-Term Care	Retrospective cohort study (chart review)	Type 1 ROP	Total: 174 ROP: 64 Treated: 3 (5.6%)	BW<1500 g or GA<30 w	**Screening HSN**: C$346/exam (SD: C$306) **Screening RVH**: C$375/exam (SD: C$300)	Health system: direct costs (excluding equipment and maintenance)
4	Kelkar (2017a) [17]	India (2009–2011) Mobile ROP screening unit	Public health intervention[b] from case series	ICROP guidelines	Total: 104 ROP: 34 Treated: 5 (15%)	BW<1700 g or GA<34 w	**Screening**: US$240/exam[c]	Health system: direct healthcare costs

For peer review only - http://bmjopen.bmj.com/site/about/guidelines.xhtml

							**Identifying an infant with ROP:** US$735/infant[c] **Treatment**: US$6500/infant	(including salaries and equipment)
5	Kelkar (2017b) [18]	India (2013–2015) Data from 5 NICUs	Public health intervention[b] from case series	ICROP guidelines	Total: 102 ROP: 32 Treated: 4 (15%)	BW<1700 g or GA<34 w	**Screening**: US$199/infant[d] **Identifying an infant with ROP**: US$596/infant[d] **Treatment**: US$4137/infant	Health system: direct costs (including salaries and equipment)
6	Rothschild (2016) [19]	Mexico and US (2014) Data from pediatric eye	Decision Analytical Model from case series	ROP caused blindness (WHO)	Total: 95	BW<1500 g	**US screening**: US$981/infant **Mexico screening:** US$333/infant	Third party payer: charges (including

For peer review only - http://bmjopen.bmj.com/site/about/guidelines.xhtml

		clinics and schools for the blind in Atlanta, Georgia, and Mexico City Blindness costs from the literature[33] and other secondary sources.					**US treatment**: US$4037/infant **Mexico treatment:** US$505/infant **US follow-up**: US$1538/infant **Mexico follow-up**: US$2214/infant **US blindness cost**: US$84586/infant **Mexico blindness cost:** US$24413/infant	labor and equipment) Societal costs: expenses for raising a blind child
7	van der Akker-van Merle	Netherlands (2009) Data from NEDROP study	Retrospective cohort study	ICROP guidelines	Total: 1380 ROP: 29 Treated: 17 (59%)	GA<32 w or BW<1500 g	**Screening**: €109/exam **Treatment**: €2755/infant	Health system: direct costs

For peer review only

	(2015) [20]	and PRN database						
8	Wongwai (2015) [21]	Thailand (2013) Hypothetical data and cohort Blindness costs using secondary data on annual government subsidies and utilities from the literature[34]	Decision Analytical Model from prospective cohort study	ET-ROP criteria	Total: 100 ROP: 9		**Screening**: THB 142/infant **Treatment**: THB (SE) 1053 (316)/infant **Lifetime cost of blindness**: THB 146,000 **Telemedicine screening**: THB 17,397/QALY (3% disc. rate)	Third party payer: charges (including labor and equipment)
9	Black (2015) [22]	US (2001–2010) Medical University of South Carolina	Retrospective cohort study	ROP stage 4	Total: 4292 ROP: 7 Treated: 7 (100%)	GA: 23–37 w	**Cost increase due to ROP if:** **GA (23 w):** US$19,513	Hospital: direct costs

For peer review only - http://bmjopen.bmj.com/site/about/guidelines.xhtml

							**GA (mean, 34.3 w):** US$23,121 **GA (37 w):** US$41,161	
10	Zin (2014) [23]	Brazil (2004–2006) 6 NICUs in Rio de Janeiro	Decision Analytical Model from case series and expert opinion	ICROP criteria	Total: 869 ROP: 70 Treated: 70 (100%)	BW<1500 g	**Screening**: US$18/infant **Treatment**: US$398/infant	Health system: direct costs (including labor and equipment)
11	Dave (2012) [24]	Peru (2009) Data from local hospital's NICU and from 2002 study[39]	Retrospective cohort study	ROP stage 1–5 with/without plus disease	Total: 1239 ROP: 80		**Screening and treatment**: US$2496/infant **Follow-up** (3 visits): US$54 **ROP caused blindness:** US$123,806/infant	Health system: direct costs (including equipment, facility, labor and supplies)

For peer review only - http://bmjopen.bmj.com/site/about/guidelines.xhtml

		Secondary source for blindness costs[35]						Societal costs: expenses for blindness
12	Dunbar (2009)[25]	US (2004–2006) Medicare and Medicaid reimbursement data from California and Louisiana	Microsimulation model from retrospective cohort study	Type 1 ROP	Total: 515 ROP: 58 Treated: 58 (100%)	BW<1500 g or GA<28 w	**Screening**: US$93/exam **Screening**: US$316/infant **Treatment w/o anesthesia**: US$1371/infant **Screening and treatment:** US$1565/QALY (3% disc. rate)	Third-party payer (Medicare and Medicaid): charges (excluding anesthesia)

For peer review only - http://bmjopen.bmj.com/site/about/guidelines.xhtml

13	Kamholz (2009) [26]	US (2005) Data from ET-ROP study	Decision Analytical Model from randomized trial and expert opinion	Type 1 ROP	ROP: 357	BW<1250 g or GA<32 w	**Screening**: US$189/exam (US$56–$251); **treatment w/o anesthesia**: US$2423 (US$638–$3223) **Anesthesia**: US$1849 (US$925–$3698)	Third-party payer: charges
14	Jackson (2008) [27]	US (2006) Data from CRYO-ROP and ET-ROP studies	Decision Analytical Model from randomized trial	Type 1 ROP	Refer to published data on 4099 infants (65.8% with ROP[36]) and 6998 infants (68% with ROP[37])	BW<1251g	**Screening**: US$160/exam **Screening and treatment**: US$4410/QALY (3% disc. rate.)	Third-party payer (Medicare): charges

For peer review only - http://bmjopen.bmj.com/site/about/guidelines.xhtml

15	Yanowitch (2006) [28]	US (2001–2004) Data from Dean A. McGee Eye Institute and OUHSC campus	Retrospective cohort study (chart review)	CRYO-ROP and ET-ROP criteria	Total: 259 ROP: 11 Treated: 1 (9%)	BW 1250–1800 g	**Screening**: US$230/infant **Treatment**: US$2000/infant	Third-party payer: charges
16	Castillo-Riquelme (2004) [29]	UK (1997-1998) Data from published data[38] and local NICU	Decision Analytical Model from case series and expert opinion	ROP stage 3	ROP: 235	GA<32 or BW<1501 g	**Screening**: £49/exam **Screening:** £279/infant **Treatment**: £540/one eye **Treatment:** £702/two eyes **Follow-up** (10 years): £786/infant	Health system: direct costs (including equipment and maintenance)
17	Lee (2001) [30]	Canada (1996-1997)	Retrospective cohort study	Threshold ROP	Total: 16,424	Different criteria at	**Screening**: C$236/infant **Treatment**: C$2655/infant	Health system: direct costs

For peer review only - http://bmjopen.bmj.com/site/about/guidelines.xhtml

		Data from 14 NICU				different NICU		
18	Brown (1999) [31]	US (1998) Database from Pennsylvania	Microsimulation model from randomized trial	Threshold ROP	ROP: 291 Treated: 291 (100%) but only one treated eye per infant	BW<1251 g	**Treatment**: US$1452/infant **Treatment consultation**: US$140/exam **Treatment**: US$678/QALY (3% disc. rate)	Third-party payer: charges
19	Javitt (1993) [32]	US (1989) Medicare reimbursement data	Microsimulation model from retrospective cohort study	Threshold ROP or PNA 24 weeks from CRYO-ROP	Total: 18,220 ROP: 1000 Treated: 1000 (100%)	BW: 500–1249 g	**Screening (1st visit)**: US$84/exam **Screening (subsequent visit)**: US$68/exam **Screening (weekly)**: US$6045/QALY	Third-party payer: charges (excluding equipment and personnel training cost)

For peer review only - http://bmjopen.bmj.com/site/about/guidelines.xhtml

							**Screening (biweekly)**: US$3623/QALY **Screening (monthly)**: US$2488/QALY	

[a] Assumption based on methods description indicating cost data collected through survey to parents.

[b] Studies of the introduction of new screening programs.

[c] Screening costs and costs for identifying an infant with ROP are reduced by 22.6% to account for transport costs (i.e., driver and cost of van and fuel to move equipment).

[d] Screening costs and costs for identifying an infant with ROP are reduced by 0.245% to account for transport costs (i.e., fuel to move equipment).

Abbreviations: BW=birth weight; disc.=discount; GA=gestational age; HSN=Health Sciences North in Sudbury, Canada; NICU=neonatal intensive care unit; PNA=postnatal age; QALY=quality-adjusted life years; ROP=retinopathy of prematurity; RVH=Royal Victoria Hospital in Barrie, Canada; US=United States of America; WHO=World Health Organization

For peer review only - http://bmjopen.bmj.com/site/about/guidelines.xhtml

For peer review only

Table 2. Costs for Screening for ROP Among Preterm Infants (in 2020 values)

#	First author (year)	Screening costs		Treatment costs	Evidence rating	Cost inclusion
		Mean per exam	Mean per infant	Mean per infant		
		(US$)	(US$)	(US$)		
1	Mohammadi (2021) [14]	-	-	1169	4	Charges
2	Moitry (2018) [15]	44	-	-	3	Direct cost
3	Isaac (2018) [16]	HSN: 342<br>RVH: 371	-	-	3	Direct cost **not including equipment**
4	Kelkar (2017a) [17]	253	-	6500	4	Direct cost including equipment and labor
5	Kelkar (2017b) [18]	210	-	4137	4	Direct cost including equipment and labor
6	Rothschild (2016) [19]		US: 1072<br>Mexico: 362	US: 4413<br>Mexico: 552	4	Direct cost including equipment and labor

For peer review only - http://bmjopen.bmj.com/site/about/guidelines.xhtml

7	van der Akker-van Merle (2015) [20]	160	-	4064[a]	3	Direct cost
8	Wongwai (2015) [21]	5	-	38	2	Charges including equipment and labor
9	Black (2015) [22]	-	-	-	3	-
10	Zin (2014) [23]	20	-	450	5	Direct cost including equipment and labor
11	Dave (2012) [24]	-	-	-	3	-
12	Dunbar (2009) [25]	119	405	1759	3	Charges
13	Kamholz (2009) [26]	250	-	5661[a]	5	Charges
14	Jackson (2008) [27]	205	-	-	1	Charges
15	Yanowitch (2006) [28]	-	324	2814	3	Charges
16	Castillo-Riquelme (2004) [29]	106	602	Unilateral: 1165	5	Direct cost including

For peer review only - http://bmjopen.bmj.com/site/about/guidelines.xhtml

				Bilateral: 1514		equipment and maintenance
17	Lee (2001) [30]	Unilateral: 112	-	2507	3	Direct cost
18	Brown (1999) [31]	-	-	2527	1	Charges
19	Javitt (1993) [32]	First: 183 Follow-up: 149	-	-	3	Charges

Evidence rating indicates the quality of evidence rating of included studies: 1=e.g., properly powered randomized controlled trials; 2=e.g., prospective cohort studies; 3=e.g., retrospective cohort studies; 4=case series with or without intervention or cross-sectional study; 5=e.g., opinion of respected authorities.

[a] Unit cost per treatment.

Abbreviations: HSN=Health Sciences North in Sudbury, Canada; ROP=retinopathy of prematurity; RVH=Royal Victoria Hospital in Barrie, Canada; US=United States of America

For peer review only - http://bmjopen.bmj.com/site/about/guidelines.xhtml

Figure Titles and Legends

Figure 1. Map of data availability and costs per a) screening visit and b) treatment. The map illustrates reported costs or means of reported costs per country for included studies in US$. In studies presenting only total screening cost per infant or by first/follow-up visits,[19,28,32] the cost level per screening was calculated under the assumption of four screening visits per infant. Where only screening cost per eye was reported,[30] it was duplicated to obtain the cost level per screening. In studies reporting only unit cost per treatment,[20,26] the unit cost was assumed to indicate the cost level of treatment per infant. Where costs were reported separately for unilateral and bilateral treatment,[29] a weighted mean cost was calculated assuming that 75% of treatments were bilateral.

Figure 2. Forest plot of treatment costs, by country categorization. In parentheses, ER of included studies: 1=e.g., properly powered randomized controlled trials; 2=e.g., prospective cohort studies; 3=e.g., retrospective cohort studies; 4=case series with or without intervention or cross-sectional study; 5=e.g., opinion of respected authorities. Abbreviations: CI=confidence interval; ER=Evidence rating; REML=Restricted Maximum Likelihood. Country abbreviated according to ISO code.

Figure 3. Forest plot of treatment costs, cumulative results by year, and country categorization. In parentheses, ER of included studies: 1=e.g., properly powered randomized controlled trials; 2=e.g., prospective cohort studies; 3=e.g., retrospective cohort studies; 4=case series with or without intervention or cross-sectional study; 5=e.g., opinion of respected authorities. Abbreviations: CI=confidence interval; ER=Evidence rating; REML=Restricted Maximum Likelihood. Country abbreviated according to ISO code.

For peer review only - http://bmjopen.bmj.com/site/about/guidelines.xhtml

Figure 1a) World map - cost per screening

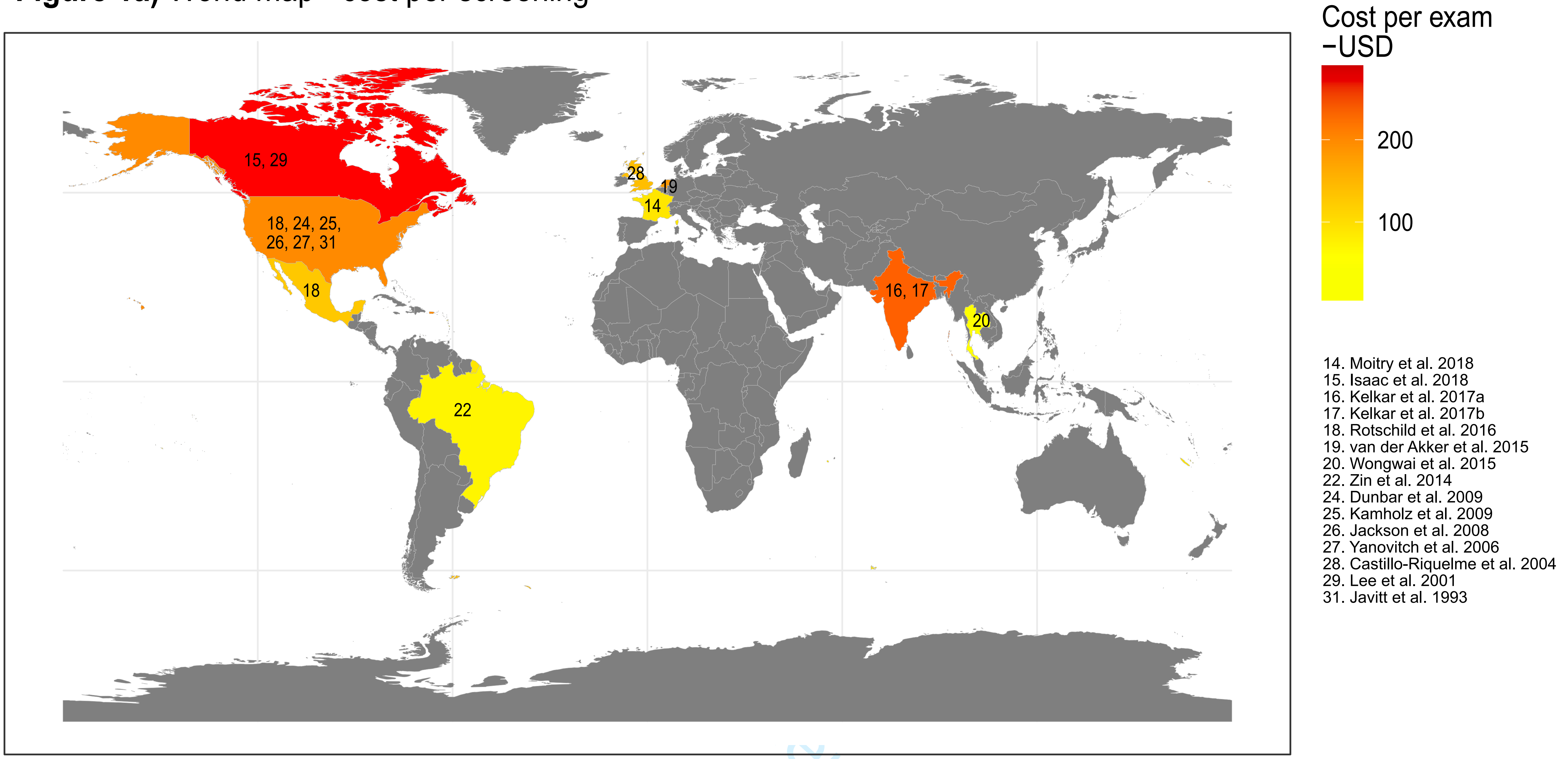

1b) World map −cost per treatment

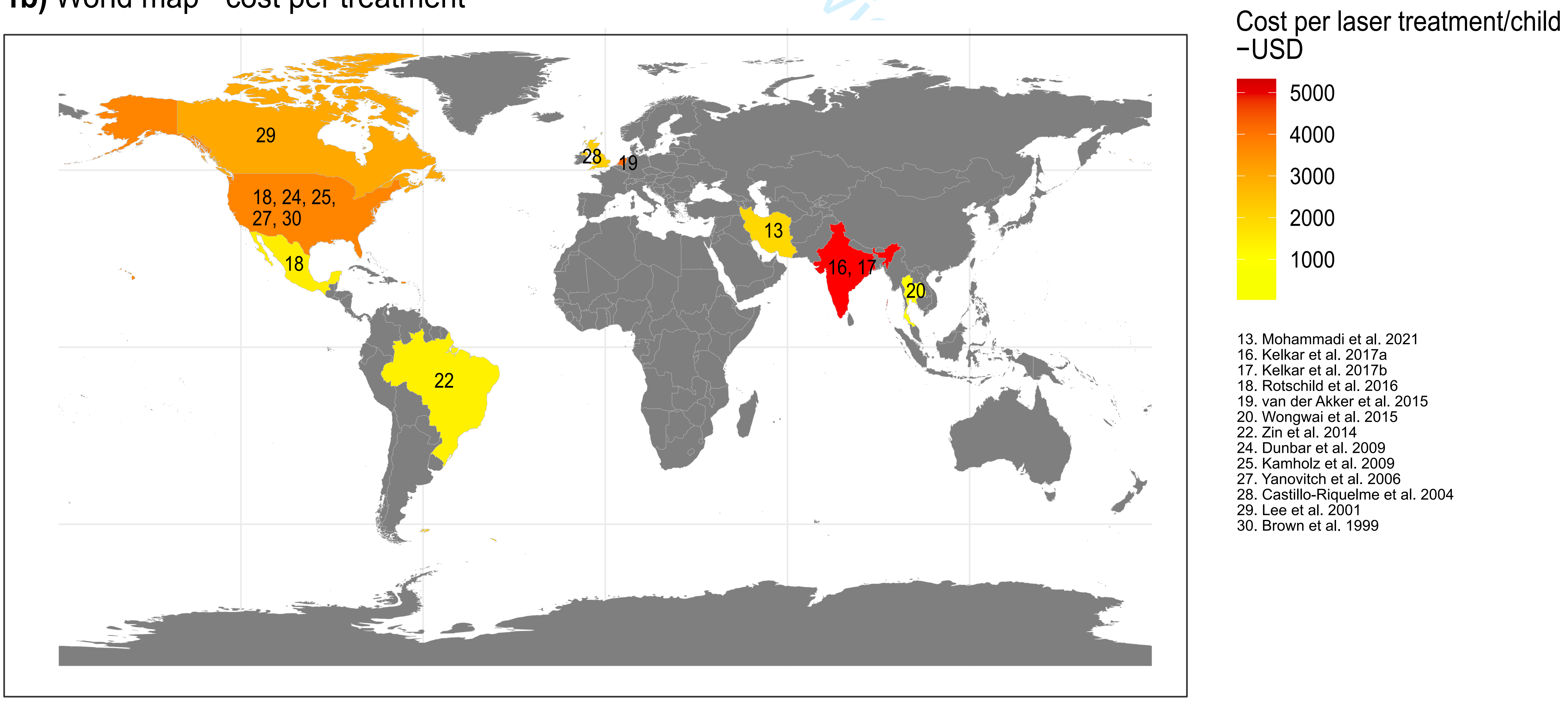

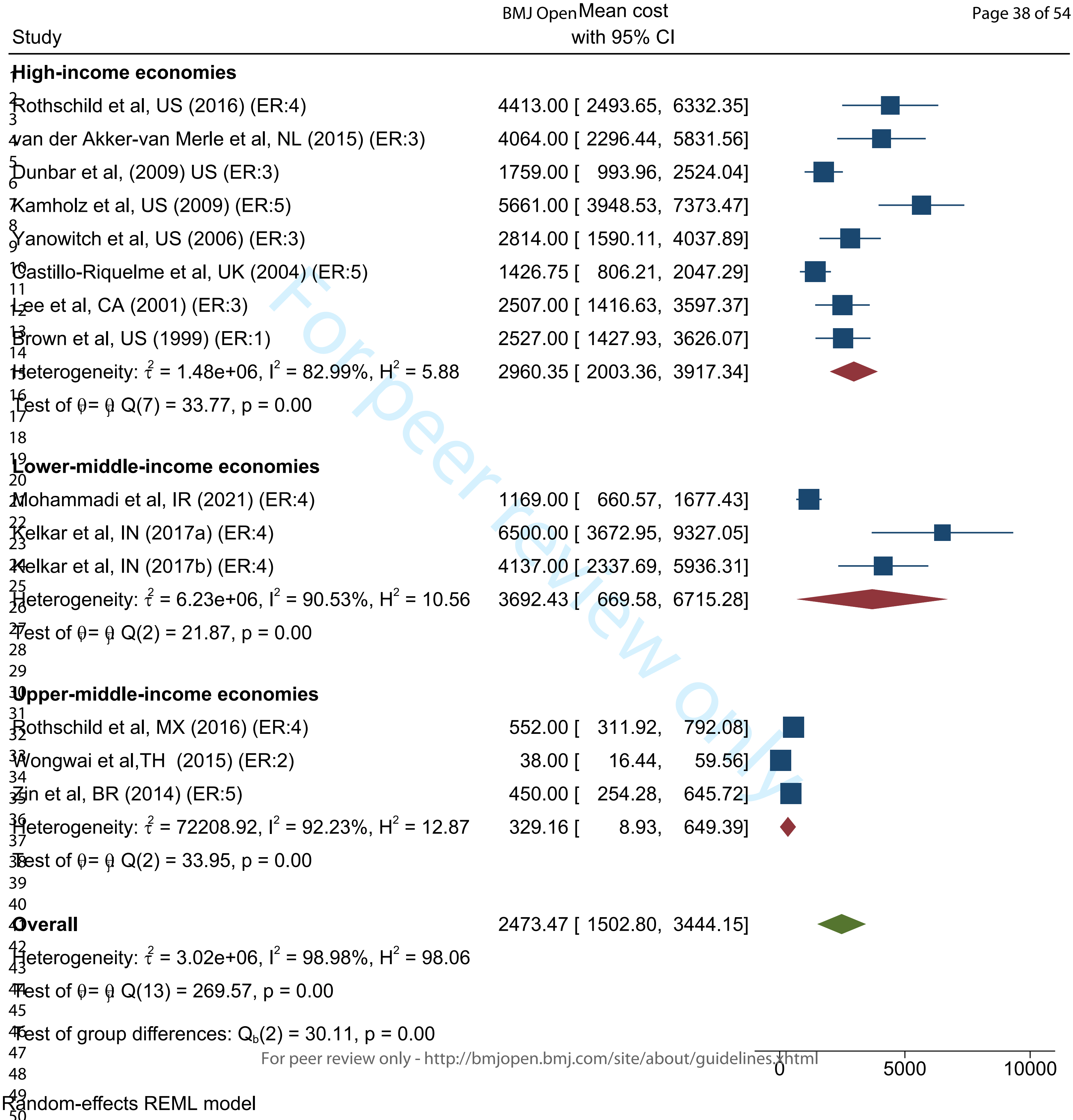
Study
Mean cost with 95% CI
High-income economies
Rothschild et al, US (2016) (ER:4) 4413.00 [2493.65, 6332.35]
van der Akker-van Merle et al, NL (2015) (ER:3) 4064.00 [2296.44, 5831.56]
Dunbar et al, (2009) US (ER:3) 1759.00 [993.96, 2524.04]
Kamholz et al, US (2009) (ER:5) 5661.00 [3948.53, 7373.47]
Yanowitch et al, US (2006) (ER:3) 2814.00 [1590.11, 4037.89]
Castillo-Riquelme et al, UK (2004) (ER:5) 1426.75 [806.21, 2047.29]
Lee et al, CA (2001) (ER:3) 2507.00 [1416.63, 3597.37]
Brown et al, US (1999) (ER:1) 2527.00 [1427.93, 3626.07]
Heterogeneity: τ^2 = 1.48e+06, I^2 = 82.99%, H^2 = 5.88 2960.35 [2003.36, 3917.34]
Test of $\theta_i = \theta_j$: Q(7) = 33.77, p = 0.00
Lower-middle-income economies
Mohammadi et al, IR (2021) (ER:4) 1169.00 [660.57, 1677.43]
Kelkar et al, IN (2017a) (ER:4) 6500.00 [3672.95, 9327.05]
Kelkar et al, IN (2017b) (ER:4) 4137.00 [2337.69, 5936.31]
Heterogeneity: τ^2 = 6.23e+06, I^2 = 90.53%, H^2 = 10.56 3692.43 [669.58, 6715.28]
Test of $\theta_i = \theta_j$: Q(2) = 21.87, p = 0.00
Upper-middle-income economies
Rothschild et al, MX (2016) (ER:4) 552.00 [311.92, 792.08]
Wongwai et al,TH (2015) (ER:2) 38.00 [16.44, 59.56]
Zin et al, BR (2014) (ER:5) 450.00 [254.28, 645.72]
Heterogeneity: τ^2 = 72208.92, I^2 = 92.23%, H^2 = 12.87 329.16 [8.93, 649.39]
Test of $\theta_i = \theta_j$: Q(2) = 33.95, p = 0.00
Overall 2473.47 [1502.80, 3444.15]
Heterogeneity: τ^2 = 3.02e+06, I^2 = 98.98%, H^2 = 98.06
Test of $\theta_i = \theta_j$: Q(13) = 269.57, p = 0.00
Test of group differences: $Q_b(2)$ = 30.11, p = 0.00
0
5000
10000
Random-effects REML model

Study	Mean cost with 95% CI
**High-income economies**	
Brown et al, US (1999) (ER:1)	2527.00 [1427.93, 3626.07]
Lee et al, CA (2001) (ER:3)	2516.92 [1742.86, 3290.99]
Castillo-Riquelme et al, UK (2004) (ER:5)	2039.56 [1240.70, 2838.41]
Yanowitch et al, US (2006) (ER:3)	2193.88 [1478.16, 2909.59]
Dunbar et al, US (2009) (ER:3)	2056.83 [1508.58, 2605.09]
Kamholz et al, US (2009) (ER:5)	2634.39 [1579.11, 3689.66]
van der Akker-van Merle et al, NL (2015) (ER:3)	2798.19 [1805.71, 3790.68]
Rothschild et al, US (2016) (ER:4)	2960.35 [2003.36, 3917.34]
**Lower-middle-income economies**	
Kelkar et al, IN (2017a) (ER:4)	6500.00 [3672.95, 9327.05]
Kelkar et al, IN (2017b) (ER:4)	5056.60 [2798.51, 7314.69]
Mohammadi et al, IR (2021) (ER:4)	3692.43 [669.58, 6715.28]
**Upper-middle-income economies**	
Zin et al, BR (2014) (ER:5)	450.00 [254.28, 645.72]
Wongwai et al,TH (2015) (ER:2)	232.05 [-171.03, 635.12]
Rothschild et al, MX (2016) (ER:4)	329.16 [8.93, 649.39]

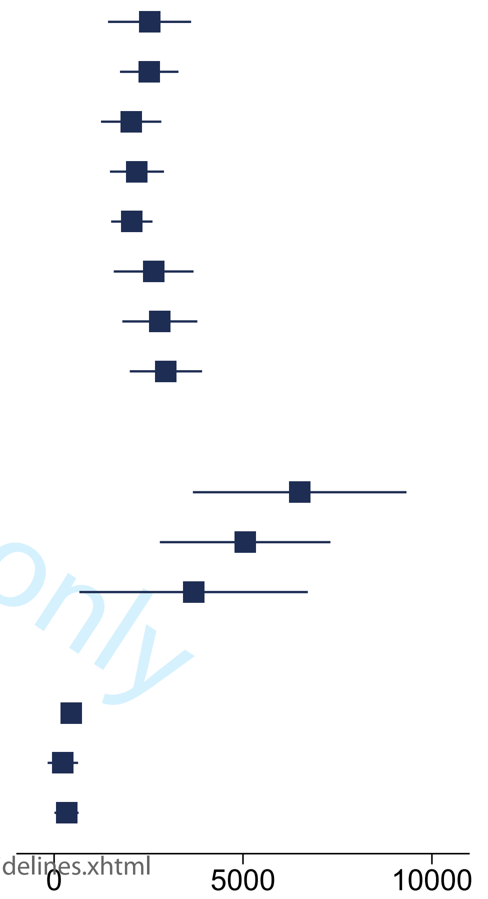

Random-effects REML model

For peer review only

Online-Only Supplements

Costs associated with Retinopathy of prematurity: A Systematic Review and Meta-analysis

Authors

Hanna Gyllensten[1,2], Associate professor

Jhangir Humayun[1,2], B.Sc.

Ulrika Sjöbom[1,3], M.Sc.

Ann Hellström[3], Professor

Chatarina Löfqvist[1,2,3], Associate professor

Author affiliations

[1] Institute of Health and Care Sciences, Sahlgrenska Academy, University of Gothenburg, Box 457, SE-405 30 Gothenburg, Sweden.

[2] Centre for Person-Centred Care (GPCC), University of Gothenburg, Box 457, SE-405 30 Gothenburg, Sweden.

[3] Department of Clinical Neuroscience, Institute of Neuroscience and Physiology, Sahlgrenska Academy, University of Gothenburg, Gothenburg, Sweden.

Content

Authors ... 1

Author affiliations ... 1

eTable 1. Search terms ... 2

eTable 2. Data extraction sheet ... 2

Data extraction ... 2

Quality assessment (according to instrument developed by Evers et al[1]) ... 2

eTable 3. Checklist for the quality appraisal of included papers (from Evers et al[1]) ... 2

eFigure 1. Flow diagram shows the study selection process, following the PRISMA guidelines.[21] ... 4

eTable 4. Excluded articles* ... 5

eFigure 2. Cost model ... 6

Preterm birth ... 7

ROP screening ... 7

Lifetime (treatment and follow-up) ... 7

References ... 8

For peer review only

eTable 1. Search strategy[a]

Database	Search string
Pubmed	(((((((Retinopathy) AND Prematur*) OR ((Terry) AND Syndrom*) OR ("ROP"[Title/Abstract]) OR "Retinopathy of Prematurity"[Mesh])) AND (("Economics"[Mesh]) OR ((economic*[Title/Abstract] OR cost[Title/Abstract] OR costs[Title/Abstract] OR costly[Title/Abstract] OR costing[Title/Abstract] OR price[Title/Abstract] OR prices[Title/Abstract] OR pricing[Title/Abstract] OR pharmacoeconomic*[Title/Abstract]))))))
Scopus	(TITLE-ABS-KEY ("Retinopath*") AND TITLE-ABS-KEY ("Prematur*")) OR (TITLE-ABS-KEY ("Retrolental") AND TITLE-ABS-KEY ("Fibroplas*")) OR (TITLE-ABS-KEY ("Terry") AND TITLE-ABS-KEY ("Syndrom*")) AND (TITLE-ABS-KEY (economic* OR cost OR cos OR costly OR costing OR price OR prices OR pricing OR pharmacoeconomic*))

[a] No filters or limitations were used in the searches of databases.

eTable 2. Data extraction sheet

Data extraction

- Reviewer
- Reference (APA)
- Aim/Objective
- Study design
- When was it conducted
- Setting including country and hospital name/database
- How is ROP severity defined
- Total study participants
- Patients with ROP (N)
- Patient group description
- Controls (N)
- Control group description
- Average cost of screening (total per infant/per visit/per eye)
- What costs are measured
- How are the costs measured
- Average Cost for infants with diagnosed sight-threatening ROP
- What costs are measured
- How are the costs measured
- Costs from which year (if adjusted, which year)
- Perspective: cost analysis
- Time horizon of cost analysis
- Funding
- Limitations: Confounders and biases reported
- Conclusions (by author)

Quality assessment (according to instrument developed by Evers et al[1])

1. Is the study population clearly described?
2. Are competing alternatives clearly described?
3. Is a well-defined research question posed in answerable form?
4. Is the economic study design appropriate to the stated objective?
5. Is the chosen time horizon appropriate in order to include relevant costs and consequences?
6. Is the actual perspective chosen appropriate?
7. Are all important and relevant costs for each alternative identified?
8. Are all costs measured appropriately in physical units?
9. Are costs valued appropriately?
10. Are all important and relevant outcomes for each alternative identified?
11. Are all outcomes measured appropriately?
12. Are outcomes valued appropriately?
13. Is an incremental analysis of costs and outcomes of alternatives performed?
14. Are all future costs and outcomes discounted appropriately?
15. Are all important variables, whose values are uncertain, appropriately subjected to sensitivity analysis?
16. Do the conclusions follow from the data reported?
17. Does the study discuss the generalizability of the results to other settings and patient/client groups?
18. Does the article indicate that there is no potential conflict of interest of study researcher(s) and funder(s)?
19. Are ethical and distributional issues discussed appropriately

For peer review only

eTable 3. Checklist for the quality appraisal of included papers (from Evers et al[1])

First authors / Checklist items[a]	Black[2]	Brown[3]	Castillo-Requilme[4]; Javitt[5]; Lee[6]; Rothchild[7]; Wongwai[8]	Dave[9]	Dunbar[10]	Isaac[11]	Kamholz[12]; Jackson[13]	Kelkar (2017a)[14]; Kelkar (2017b)[15]	Mohammadi[16]	Moitry[17]	Van den Akker-van Merle[18]	Yanowitch[19]	Zin[20]	**Total**
1	+	+	+	+	+	+	-	+	-	+	+	+	+	**16**
2	+	+	+	+	+	+	+	+	+	+	+	+	+	**19**
3	+	+	+	+	+	+	+	+	+	+	+	+	+	**19**
4	+	+	+	+	+	+	+	+	+	+	+	+	+	**19**
5	+	+	+	+	+	+	+	+	+	+	+	+	+	**19**
6	+	+	+	+	+	+	+	+	-	+	+	+	+	**18**
7	+	+	+	+	+	+	+	+	+	+	+	+	+	**19**
8	+	+	+	+	+	+	+	+	+	+	+	+	+	**19**
9	+	+	+	+	+	+	+	+	+	+	+	+	+	**19**
10	+	+	+	+	+	+	+	+	+	+	+	+	+	**18**
11	+	+	+	+	+	+	+	+	+	+	+	+	+	**19**
12	+	+	+	+	+	+	+	+	+	+	+	+	+	**19**
13	+	-	+	+	+	+	+	-	-	-	+	+	+	**14**
14	-	-	+	-	+	-	+	-	-	+	+	+	-	**11**
15	+	-	+	-	-	-	+	-	-	+	-	-	+	**10**
16	+	+	+	+	+	+	+	+	+	+	+	+	+	**19**
17	+	+	+	+	+	+	+	+	+	+	+	+	+	**19**
18	+	+	+	+	-	+	+	-	+	+	-	+	+	**15**

For peer review only

19	+	+	+	+	+	+	+	-	+	+	+	+	+	**17**
**Total**	**18**	**16**	**19**	**17**	**17**	**17**	**18**	**14**	**14**	**18**	**17**	**17**	**18**	

[a] Item numbering (also in eTable 2): 1. Is the study population clearly described?; 2. Are competing alternatives clearly described?; 3. Is a well-defined research question posed in answerable form?; 4. Is the economic study design appropriate to the stated objective?; 5. Is the chosen time horizon appropriate in order to include relevant costs and consequences?; 6. Is the actual perspective chosen appropriate?; 7. Are all important and relevant costs for each alternative identified?; 8. Are all costs measured appropriately in physical units?; 9. Are costs valued appropriately?; 10. Are all important and relevant outcomes for each alternative identified?; 11. Are all outcomes measured appropriately?; 12. Are outcomes valued appropriately?; 13. Is an incremental analysis of costs and outcomes of alternatives performed?; 14. Are all future costs and outcomes discounted appropriately?; 15. Are all important variables, whose values are uncertain, appropriately subjected to sensitivity analysis?; 16. Do the conclusions follow from the data reported?; 17. Does the study discuss the generalizability of the results to other settings and patient/client groups?; 18. Does the article indicate that there is no potential conflict of interest of study researcher(s) and funder(s)?; 19. Are ethical and distributional issues discussed appropriately?

For peer review only

eFigure 1. Flow diagram shows the study selection process, following the PRISMA guidelines.[21]

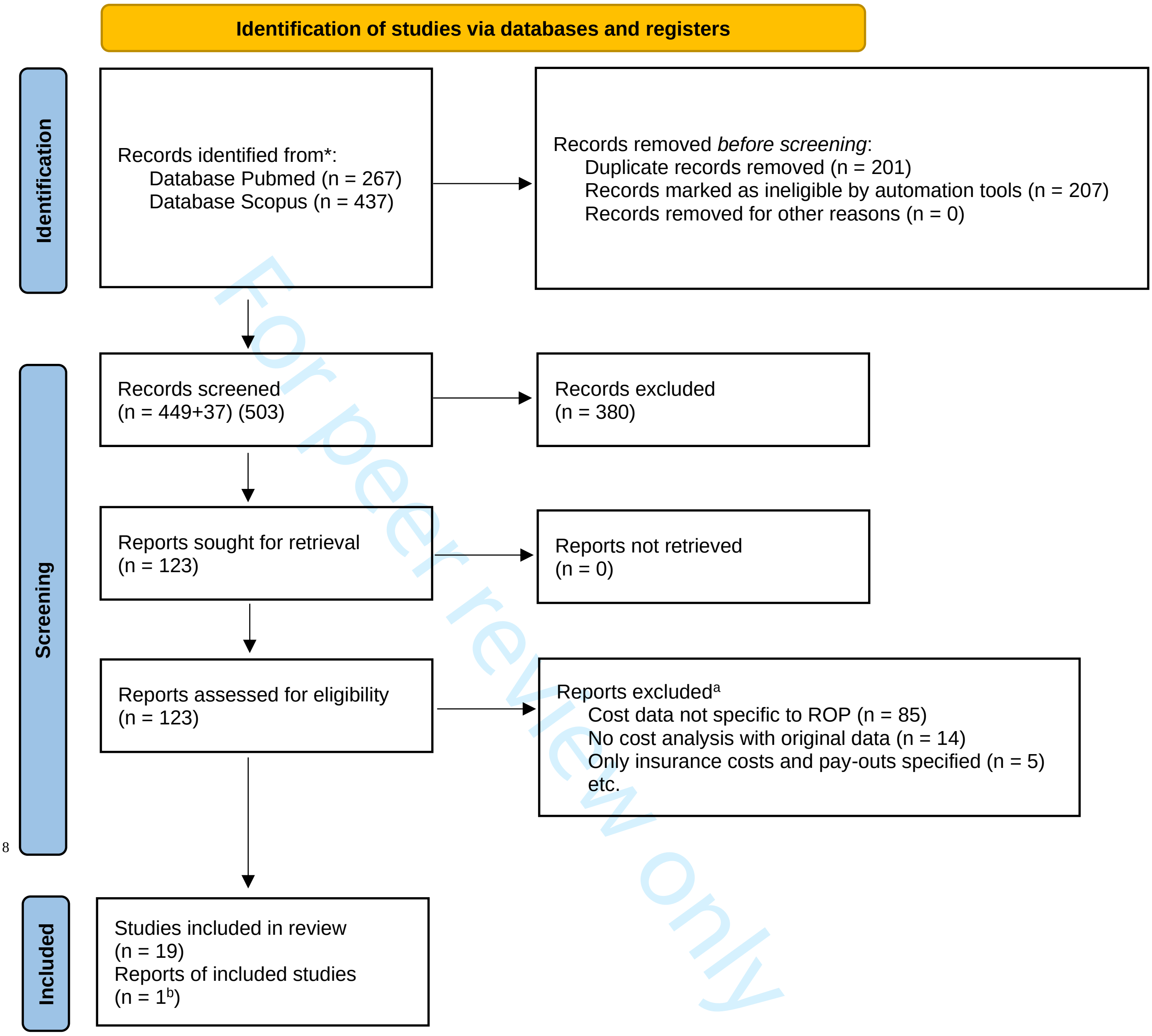

For peer review only

[a] For detailed reasons for exclusion of studies that might appear to meet the inclusion criteria, but which were excluded, see also eTable 4.

[b] One author[8] was contacted and clarified the currency of reported results. Another author[16] was unsuccessfully contacted to clarify cost perspective.

Abbreviations: ROP = Retinopathy of prematurity.

eTable 4. Excluded articles[a]

Study	Reason for exclusion
Cross 1973. Cost of preventing retrolental fibroplasia?[22]	No original cost data.
Boncz et al., 2013. [Health-economic analysis of diseases related to disturbed neonatal adaptation: A cost of illness study].[23]	Only insurance payouts.
Yo et al., 2018. Retinopathy of prematurity: the high cost of screening regional and remote infants.[24]	Transport costs but no screening costs.
Scholz and Greiner, 2019. An exclusive human milk diet for very low birth weight newborns-A cost-effectiveness and EVPI study for Germany.[25]	No ROP specific costs.
Zupancic et al., 2020. Evaluation of the economic impact of modified screening criteria for retinopathy of prematurity from the Postnatal Growth and ROP (G-ROP) study.[26]	No original cost data.

[a] In this table are listed studies that might appear to meet the inclusion criteria, but which were excluded, and why they were excluded.

Abbreviations: ROP = Retinopathy of prematurity.

For peer review only

eFigure 2. Cost model

This figure presents our preliminary suggestions for a conceptual model for costs associated with retinopathy of prematurity (ROP), with some additional comments we believe are relevant. Abbreviations: GA=gestational age; ROP=retinopathy of prematurity; VEGF=vascular endothelial growth factor.

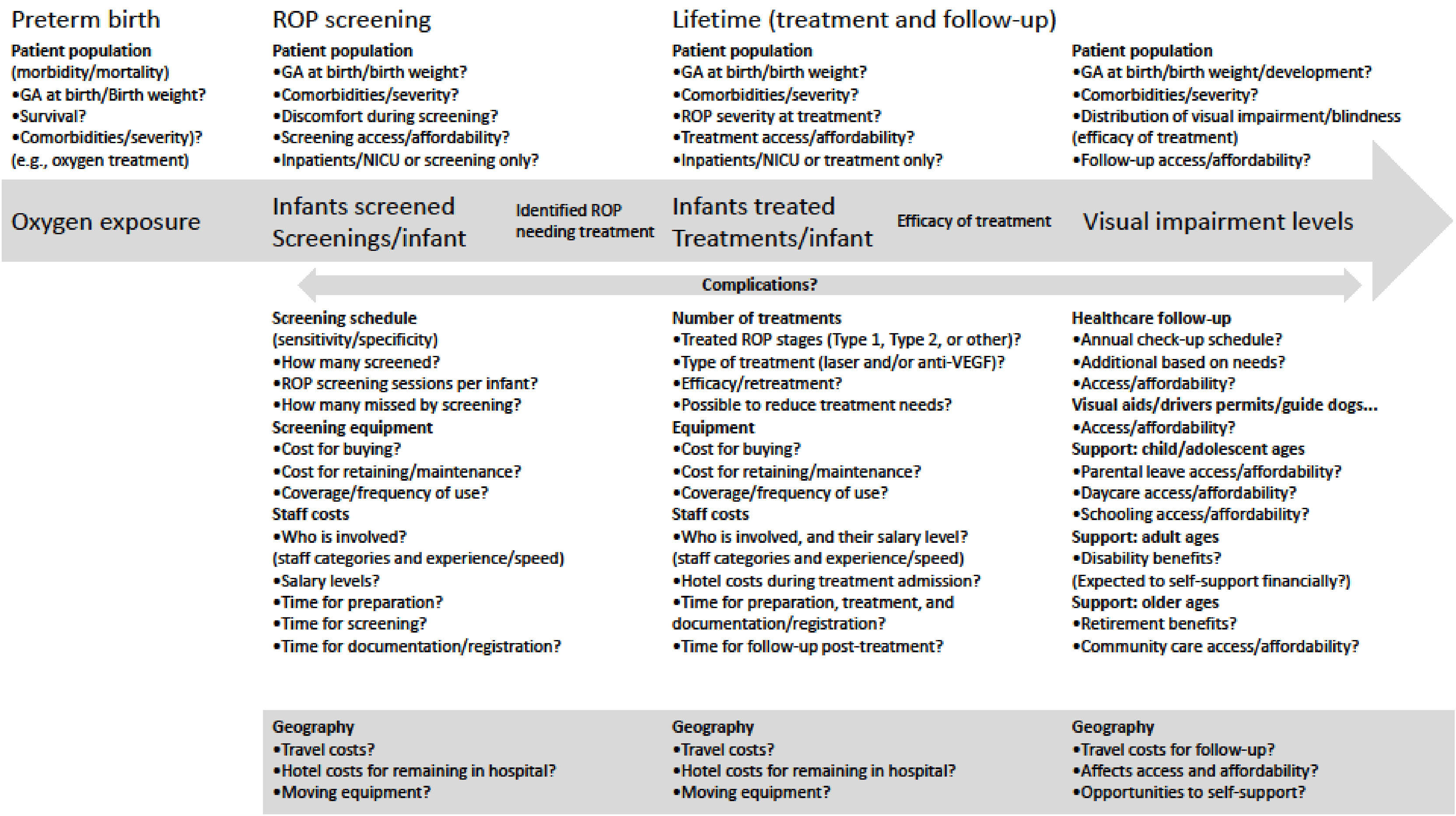

Preterm birth

It should be noted that these costs are part of a larger picture of understanding the economic impact of prematurity, which is essential knowledge in predicting the costs and consequences of introducing new interventions that affect gestational age at birth or morbidity and mortality among preterm infants. Thus, the model here is only one part and should be complemented by factors related to, e.g., bronchopulmonary dysplasia and other lung diseases, as well as other neuropsychiatric conditions. The listed items add to the previously published compartmental model of the global burden of ROP,[27] which also accounts for e.g., availability and coverage of screening programs.

ROP screening

Some evidence suggests that screening can be reduced even as infants are still identified with high sensitivity and specificity.[5] Reduced screening can be achieved through either changing the frequency of screening or limiting who is actually screened. Based on register findings in Sweden, infants born after gestational week 30 are no longer routinely screened for ROP.[28] Similarly, a study from the Netherlands found no severe ROP among infants born ≥30 gestational weeks.[29] This pattern differs from the situation in many other parts of the world. However, infants born at lower gestational age are more likely to develop ROP and severe ROP.[30]

Costs for screening in the studies included staff salaries/time, equipment and maintenance, supplies, and staff training. Although the identified studies do not detail the cost components and their associated costs, it can be expected that the reported costs of screening are to some extent underestimated. In time-and-motion studies conducted in our local hospital during a process of developing services (unpublished results), the times spent for preparatory work and documentation of screening results were 7–15 minutes and 7–12 minutes, respectively. This range included the time needed to identify infants who should be screened from those born at the facility, but excluded the time used for the actual screening. The figures can be compared to numbers provided in, e.g., Wongwai et al.,[8] citing 10 minutes used for screening by the ophthalmologist and 60 minutes for the nurse. According to Jackson et al.,[13] an average five examinations were necessary for determining if one infant would require treatment for ROP, which is in line with experiences in our hospital.

Regardless of the setting, there will also be transportation costs associated with screening. In this review, we excluded transportation costs, which are highly specific to each setting. For example, an Australian study reported flights for ROP screening to average 36–75 minutes depending on the healthcare center.[24] Transportation can thus include the time and expenses to the families coming into the hospital (or to visit a telemedicine center), or moving within the hospital if the infant remains hospitalized, but they can also reflect the cost of a specialized physician and assistant nurse or other staff category moving within or between hospitals to conduct screening. In addition to being an important cost component to consider in evaluations, the transportation aspect and hotel costs for staying in the hospital can directly affect screening. Our group has clinical experience of parents selecting not to attend planned screening visits after leaving the hospital, so that travel costs also become an issue related to increasing screening adherence and motivating attendance.

Lifetime (treatment and follow-up)

Treatment costs in individual studies included, e.g., staff salaries/time, equipment and maintenance, supplies, and staff training. Few studies reported detailed data on cost components, but Wongwai et al.,[8] for example, reported post-screening resource use of 60 minutes for an expert ophthalmologist, which we interpret to be the cost for treatment. Although case-mix and survival of extremely preterm infants were not detailed in the included studies, it can be expected that these factors will affect how many infants need treatment for ROP. For example, among infants born ≤30 gestational weeks in Sweden, 32% had any stage ROP and 6% were treated for ROP,[28] but among infants born at <24 gestational weeks, the corresponding figures were 92% and 43%.[31] Moreover, the available treatment options would affect costs, with studies suggesting, e.g., more retreatments with the more recent anti-vascular endothelial growth factor (VEGF) therapy.[28] Surgical intervention, or vitrectomy, could also apply to more severe cases,[32] in particular in countries with low access to screening. Although the costs of vitrectomy itself appear to be low,[33] there are likely other costs associated with these severe ROP cases, such as those linked to follow-up and complications.[34]

The argument regarding transportation costs is highly relevant for the treatment of ROP. The clinical reality of many countries is that patients

For peer review only

must be flown to the treatment site, or undergo multiple relocations by ambulance between local hospitals and specialized units providing the treatment.

At least in countries with high access to healthcare, it can be expected that children with ROP, and particularly those with severe forms requiring treatment, will have multiple follow-ups during childhood, adolescence, and possibly into adulthood. The low number of healthcare visits for follow-up indicated in the included articles differs considerably from the national guidelines in Sweden, recommending annual follow-up of ROP until adulthood and, after that, according to need. In a recent publication reporting on a model for predicting visual outcomes after ROP treatment,[35] follow-up every 6 months was even indicated for some patient groups.

Although costs for blindness can be expected to be similar regardless of the cause of blindness, data are available on approximate cost levels for different levels of visual impairment.[36] Thus, tapping into models for measuring costs of visual impairment can add to understanding of the long-term consequences of ROP.

References

1. Evers S, Goossens M, de Vet H, van Tulder M, Ament A. Criteria list for assessment of methodological quality of economic evaluations: Consensus on Health Economic Criteria. *Int J Technol Assess Health Care*. 2005;21(2):240-245.

2. Black L, Hulsey T, Lee K, Parks DC, Ebeling MD. Incremental Hospital Costs Associated With Comorbidities of Prematurity. *Manag Care*. 2015;24(12):54-60.

3. Brown GC, Brown MM, Sharma S, Tasman W, Brown HC. Cost-effectiveness of treatment for threshold retinopathy of prematurity. *Pediatrics*. 1999;104(4):e47.

4. Castillo-Riquelme MC, Lord J, Moseley MJ, Fielder AR, Haines L. Cost-effectiveness of digital photographic screening for retinopathy of prematurity in the United Kingdom. *Int J Technol Assess Health Care*. 2004;20(2):201-213.

5. Javitt J, Cas RD, Chiang YP. Cost-effectiveness of screening and cryotherapy for threshold retinopathy of prematurity. *Pediatrics*. 1993;91(5):859-866.

6. Lee SK, Norm, C., et al. Evidence for changing guidelines for routine screening for retinopathy of prematurity. *Arch Pediatr Adolesc Med*. 2001;155(3):387-395.

7. Rothschild MI, Russ R, Brennan KA, et al. The Economic Model of Retinopathy of Prematurity (EcROP) Screening and Treatment: Mexico and the United States. *Am J Ophthalmol*. 2016;168:110-121.

8. Wongwai P, Kingkaew P, Asawaphureekorn S, Kolatat T. A store-and-forward telemedicine for retinopathy of prematurity screen: Is it cost-effective in Thailand? *Asian Biomedicine*. 2015;9(5):665-673.

9. Dave HB, Gordillo L, Yang Z, Zhang MS, Hubbard GB 3rd, Olsen TW. The societal burden of blindness secondary to retinopathy of prematurity in Lima, Peru. *Am J Ophthalmol*. 2012;154(4):750-755.

10. Dunbar JA, Hsu V, Christensen M, Black B, Williams P, Beauchamp G. Cost-utility analysis of screening and laser treatment of retinopathy of prematurity. *Journal of AAPOS*. 2009;13(2):186-190.

11. Isaac M, Isaranuwatchai W, Tehrani N. Cost analysis of remote telemedicine screening for retinopathy of prematurity. *Can J Ophthalmol*. 2018;53(2):162-167.

12. Kamholz KL, Cole CH, Gray JE, Zupancic JA. Cost-effectiveness of early treatment for retinopathy of prematurity. *Pediatrics*. 2009;123(1):262-269.

13. Jackson KM, Scott KE, Graff Zivin J, et al. Cost-utility analysis of telemedicine and ophthalmoscopy for retinopathy of prematurity management. *Arch Ophthalmol*. 2008;126(4):493-499.

For peer review only

14. Kelkar J, Agashe S, Kelkar A, Kh, ekar R. Mobile unit for retinopathy of prematurity screening and management at urban Neonatal Intensive Care Units: Outcomes and impact assessment. *Oman J Ophthalmol*. 2017;10(1):13-16.

15. Kelkar J, Kelkar A, Sharma S, Dewani J. A mobile team for screening of retinopathy of prematurity in India: Cost - effectiveness, outcomes, and impact assessment. *Taiwan J Ophthalmol*. 2017;7(3):155-159.

16. Mohammadi S-F, Rahban A, Darabeigi S, et al. Cost-effectiveness analysis of tele-retinopathy of prematurity screening in Iran. *Int J Ophthalmol*. 2021;14(4):560-566. doi:10.18240/ijo.2021.04.13

17. Moitry M, Zarca K, Granier M, et al. Effectiveness and efficiency of tele-expertise for improving access to retinopathy screening among 351 neonates in a secondary care center: An observational, controlled before-after study. *PLoS One*. 2018;13(10):e0206375.

18. van den Akker-van Marle ME, van Sorge AJ, Schalij-Delfos NE. Cost and effects of risk factor guided screening strategies for retinopathy of prematurity for different treatment strategies. *Acta Ophthalmol*. 2015;93(8):706-712.

19. Yanovitch TL, Siatkowski RM, McCaffree M, Corff KE. Retinopathy of prematurity in infants with birth weight>or=1250 grams-incidence, severity, and screening guideline cost-analysis. *J aapos*. 2006;10(2):128-134.

20. Zin AA, Magluta C, Pinto MF, et al. Retinopathy of prematurity screening and treatment cost in Brazil. *Rev Panam Salud Publica*. 2014;36(1):37-43.

21. Page MJ, McKenzie JE, Bossuyt PM, et al. The PRISMA 2020 statement: an updated guideline for reporting systematic reviews. *BMJ*. 2021;372:n71. doi:10.1136/bmj.n71

22. Cross KW. Cost of preventing retrolental fibroplasia? *Lancet*. 1973;2(7835):954-956. doi:10.1016/s0140-6736(73)92610-x

23. Boncz I, Kovács LG, Ertl T, Ágoston I, Molics B, Bódis J. Health-economic analysis of diseases related to disturbed neonatal adaptation: A cost of illness study. *Lege Artis Medicinae*. 2013;23(3-4):193-197.

24. Yu T-Y, Donovan T, Armfield N, Gole GA. Retinopathy of prematurity: the high cost of screening regional and remote infants. *Clin Exp Ophthalmol*. 2018;46(6):645-651. doi:10.1111/ceo.13160

25. Scholz SM, Greiner W. An exclusive human milk diet for very low birth weight newborns-A cost-effectiveness and EVPI study for Germany. *PLoS One*. 2019;14(12):e0226496. doi:10.1371/journal.pone.0226496

26. Zupancic JAF, Ying G-S, de Alba Campomanes A, Tomlinson LA, Binenbaum G, G-ROP Study Group. Evaluation of the economic impact of modified screening criteria for retinopathy of prematurity from the Postnatal Growth and ROP (G-ROP) study. *J Perinatol*. 2020;40(7):1100-1108. doi:10.1038/s41372-020-0605-5

27. Blencowe H, Lawn JE, Vazquez T, Fielder A, Gilbert C. Preterm-associated visual impairment and estimates of retinopathy of prematurity at regional and global levels for 2010. *Pediatr Res*. 2013;74(Suppl 1):35-49. doi:10.1038/pr.2013.205

28. Holmström G, Hellström A, Gränse L, et al. New modifications of Swedish ROP guidelines based on 10-year data from the SWEDROP register. *Br J Ophthalmol*. 2020;104(7):943-949. doi:10.1136/bjophthalmol-2019-314874

29. Trzcionkowska K, Groenendaal F, Andriessen P, et al. Risk Factors for Retinopathy of Prematurity in the Netherlands: A Comparison of Two Cohorts. *Neonatology*. 2021;118(4):462-469. doi:10.1159/000517247

30. Isaza G, Arora S. Incidence and severity of retinopathy of prematurity in extremely premature infants. *Canadian Journal of Ophthalmology*. 2012;47(3):296-300. doi:10.1016/j.jcjo.2012.03.027

For peer review only

31. Lundgren P, Jacobson L, Hård A-L, et al. High rate and large intercentre variability in retreatment of retinopathy of prematurity in infants born <24 gestational weeks. *BMJ Open Ophthalmol*. 2021;6(1):e000695. doi:10.1136/bmjophth-2020-000695

32. Rajan RP, Kannan NB, Sen S, et al. Clinico-demographic profile and outcomes of 25-gauge vitrectomy in advanced stage 5 retinopathy of prematurity. *Graefe's Archive for Clinical and Experimental Ophthalmology*. 2021;259(7):1695-1701. doi:10.1007/s00417-020-05063-2

33. Nicod E, Jackson TL, Grimaccia F, et al. Direct cost of pars plana vitrectomy for the treatment of macular hole, epiretinal membrane and vitreomacular traction: a bottom-up approach. *European Journal of Health Economics*. 2016;17(8):991-999. doi:10.1007/s10198-015-0741-6

34. Zhao D-Y, Zhang Y-J, He Z-J, et al. Clinical analysis of apnea after operation for retinopathy of prematurity. *Journal of Shanghai Jiaotong University (Medical Science)*. 2010;30(2):132-134.

35. Huang C-Y, Kuo R-J, Li C-H, et al. Prediction of visual outcomes by an artificial neural network following intravitreal injection and laser therapy for retinopathy of prematurity. *British Journal of Ophthalmology*. 2020;104(9):1277-1282. doi:10.1136/bjophthalmol-2019-314860

36. Köberlein J, Beifus K, Schaffert C, Finger RP. The economic burden of visual impairment and blindness: a systematic review. *BMJ Open*. 2013;3(11):e003471. doi:10.1136/bmjopen-2013-003471

For peer review only

PRISMA checklist

Section and Topic	Item #	Checklist item	Location where item is reported
**TITLE**			
Title	1	Identify the report as a systematic review.	Title
**ABSTRACT**			
Abstract	2	See the PRISMA 2020 for Abstracts checklist.	[See below]
**INTRODUCTION**			
Rationale	3	Describe the rationale for the review in the context of existing knowledge.	Page 3
Objectives	4	Provide an explicit statement of the objective(s) or question(s) the review addresses.	Page 3
**METHODS**			
Eligibility criteria	5	Specify the inclusion and exclusion criteria for the review and how studies were grouped for the syntheses.	Page 3
Information sources	6	Specify all databases, registers, websites, organisations, reference lists and other sources searched or consulted to identify studies. Specify the date when each source was last searched or consulted.	Page 3
Search strategy	7	Present the full search strategies for all databases, registers and websites, including any filters and limits used.	eTable 1
Selection process	8	Specify the methods used to decide whether a study met the inclusion criteria of the review, including how many reviewers screened each record and each report retrieved, whether they worked independently, and if applicable, details of automation tools used in the process.	Page 3
Data collection process	9	Specify the methods used to collect data from reports, including how many reviewers collected data from each report, whether they worked independently, any processes for obtaining or confirming data from study investigators, and if applicable, details of automation tools used in the process.	Page 3
Data items	10a	List and define all outcomes for which data were sought. Specify whether all results that were compatible with each outcome domain in each study were sought (e.g. for all measures, time points, analyses), and if not, the methods used to decide which results to collect.	eTable 2
	10b	List and define all other variables for which data were sought (e.g. participant and intervention characteristics, funding sources). Describe any assumptions made about any missing or unclear information.	eTable 2
Study risk of bias assessment	11	Specify the methods used to assess risk of bias in the included studies, including details of the tool(s) used, how many reviewers assessed each study and whether they worked independently, and if applicable, details of automation tools used in the process.	Page 3
Effect measures	12	Specify for each outcome the effect measure(s) (e.g. risk ratio, mean difference) used in the synthesis or presentation of results.	Page 3
Synthesis methods	13a	Describe the processes used to decide which studies were eligible for each synthesis (e.g. tabulating the study intervention characteristics and comparing against the planned groups for each synthesis (item #5)).	Page 3
	13b	Describe any methods required to prepare the data for presentation or synthesis, such as handling of missing summary statistics, or data conversions.	Page 3
	13c	Describe any methods used to tabulate or visually display results of individual studies and syntheses.	Page 3

For peer review only

Section and Topic	Item #	Checklist item	Location where item is reported
	13d	Describe any methods used to synthesize results and provide a rationale for the choice(s). If meta-analysis was performed, describe the model(s), method(s) to identify the presence and extent of statistical heterogeneity, and software package(s) used.	Page 3
	13e	Describe any methods used to explore possible causes of heterogeneity among study results (e.g. subgroup analysis, meta-regression).	Page 3
	13f	Describe any sensitivity analyses conducted to assess robustness of the synthesized results.	Page 3
Reporting bias assessment	14	Describe any methods used to assess risk of bias due to missing results in a synthesis (arising from reporting biases).	Not possible
Certainty assessment	15	Describe any methods used to assess certainty (or confidence) in the body of evidence for an outcome.	Page 4
**RESULTS**			
Study selection	16a	Describe the results of the search and selection process, from the number of records identified in the search to the number of studies included in the review, ideally using a flow diagram.	eFigure 1
	16b	Cite studies that might appear to meet the inclusion criteria, but which were excluded, and explain why they were excluded.	eTable 4
Study characteristics	17	Cite each included study and present its characteristics.	Page 3
Risk of bias in studies	18	Present assessments of risk of bias for each included study.	eTable 3
Results of individual studies	19	For all outcomes, present, for each study: (a) summary statistics for each group (where appropriate) and (b) an effect estimate and its precision (e.g. confidence/credible interval), ideally using structured tables or plots.	Page 4
Results of syntheses	20a	For each synthesis, briefly summarise the characteristics and risk of bias among contributing studies.	Page 4-5
	20b	Present results of all statistical syntheses conducted. If meta-analysis was done, present for each the summary estimate and its precision (e.g. confidence/credible interval) and measures of statistical heterogeneity. If comparing groups, describe the direction of the effect.	Figure 3
	20c	Present results of all investigations of possible causes of heterogeneity among study results.	Figures 1-3
	20d	Present results of all sensitivity analyses conducted to assess the robustness of the synthesized results.	Not applicable
Reporting biases	21	Present assessments of risk of bias due to missing results (arising from reporting biases) for each synthesis assessed.	Not possible
Certainty of evidence	22	Present assessments of certainty (or confidence) in the body of evidence for each outcome assessed.	Table 2 and Figure 3
**DISCUSSION**			
Discussion	23a	Provide a general interpretation of the results in the context of other evidence.	Page 5-6
	23b	Discuss any limitations of the evidence included in the review.	Page 5-6

For peer review only

Section and Topic	Item #	Checklist item	Location where item is reported
	23c	Discuss any limitations of the review processes used.	Page 5
	23d	Discuss implications of the results for practice, policy, and future research.	Page 6
**OTHER INFORMATION**			
Registration and protocol	24a	Provide registration information for the review, including register name and registration number, or state that the review was not registered.	Page 2
	24b	Indicate where the review protocol can be accessed, or state that a protocol was not prepared.	Page 3
	24c	Describe and explain any amendments to information provided at registration or in the protocol.	Not applicable
Support	25	Describe sources of financial or non-financial support for the review, and the role of the funders or sponsors in the review.	Page 7
Competing interests	26	Declare any competing interests of review authors.	Page 7
Availability of data, code and other materials	27	Report which of the following are publicly available and where they can be found: template data collection forms; data extracted from included studies; data used for all analyses; analytic code; any other materials used in the review.	Page 7

For peer review only

PRISMA abstract checklist

Section and Topic	Item #	Checklist item	Reported (Yes/No)
**TITLE**			
Title	1	Identify the report as a systematic review.	Title
**BACKGROUND**			
Objectives	2	Provide an explicit statement of the main objective(s) or question(s) the review addresses.	Objective
**METHODS**			
Eligibility criteria	3	Specify the inclusion and exclusion criteria for the review.	Study selection
Information sources	4	Specify the information sources (e.g. databases, registers) used to identify studies and the date when each was last searched.	Data sources
Risk of bias	5	Specify the methods used to assess risk of bias in the included studies.	Data Extraction and Synthesis
Synthesis of results	6	Specify the methods used to present and synthesise results.	Data Extraction and Synthesis
**RESULTS**			
Included studies	7	Give the total number of included studies and participants and summarise relevant characteristics of studies.	Results
Synthesis of results	8	Present results for main outcomes, preferably indicating the number of included studies and participants for each. If meta-analysis was done, report the summary estimate and confidence/credible interval. If comparing groups, indicate the direction of the effect (i.e. which group is favoured).	Results
**DISCUSSION**			
Limitations of evidence	9	Provide a brief summary of the limitations of the evidence included in the review (e.g. study risk of bias, inconsistency and imprecision).	Conclusions and Relevance
Interpretation	10	Provide a general interpretation of the results and important implications.	Conclusions and Relevance
**OTHER**			
Funding	11	Specify the primary source of funding for the review.	[In funding statement]
Registration	12	Provide the register name and registration number.	Registration

For peer review only

Section and Topic	Item #	Checklist item	Reported (Yes/No)
			number in PROSPERO

For peer review only
